# Dissecting Sex Chromosome and Hormonal Contributions to Urethane-Induced Lung Tumorigenesis Using the Four Core Genotypes Mouse Model

**DOI:** 10.3390/cancers18071172

**Published:** 2026-04-05

**Authors:** Maksat Babayev, Omar A. Borges-Sosa, Carolyn D. Ekpruke, Erik Parker, Dustin Rousselle, Lyidia Dinwiddie, Rachel Alford, Shikha Sharma, Praveen Chirumamilla, Michelle C. Boulos, Aakash Parekh, Matthew L. Retzner, Catherine R. Sears, James E. Klaunig, Sarah Commodore, Patricia Silveyra

**Affiliations:** 1 Department of Environmental and Occupational Health, School of Public Health-Bloomington, Indiana University, Bloomington, IN 47405, USA; mbabayev@iu.edu (M.B.); oborges@iu.edu (O.A.B.-S.); cekpruke@iu.edu (C.D.E.); drousse@iu.edu (D.R.); didinwi@iu.edu (L.D.); rachalfo@iu.edu (R.A.); prchiru@iu.edu (P.C.); mboulos@iu.edu (M.C.B.); aakparek@iu.edu (A.P.); matretzn@iu.edu (M.L.R.); jklauni@iu.edu (J.E.K.); scommod@iu.edu (S.C.); 2 Biostatistics Consulting Center, Department of Epidemiology and Biostatistics, School of Public Health-Bloomington, Indiana University, Bloomington, IN 47405, USA; erikpark@iu.edu; 3 Department of Forensic Science, Henry C. Lee College of Criminal Justice and Forensic Sciences, University of New Haven, West Haven, CT 06516, USA; ssharma@newhaven.edu; 4 Pulmonary and Critical Care Section, Department of Medicine, Richard L. Roudebush VA Medical Center, Indianapolis, IN 46202, USA; crufatto@iu.edu; 5 Division of Pulmonary, Critical Care, Sleep and Occupational Medicine, Indiana University School of Medicine, Indianapolis, IN 46202, USA; 6 Indiana University Melvin and Bren Simon Comprehensive Cancer Center, Indiana University School of Medicine, Indianapolis, IN 46202, USA

**Keywords:** four core genotypes, lung tumorigenesis, sex differences, mouse models, urethane, KRAS

## Abstract

Background/Objectives: Sex differences in lung cancer incidence and outcomes are well recognized, yet the relative contributions of sex chromosomes and gonadal sex remain incompletely defined. We aimed to disentangle chromosomal complement and hormonal sex in urethane-induced lung tumorigenesis using the Four Core Genotypes mouse model. Methods: Mice (6–8 weeks old) with independently varied chromosomal complement (XX vs. XY) and gonadal sex received urethane (1 g/kg body weight) weekly for 10 weeks and were evaluated after a 20-week latency period. Tumor multiplicity, tumor area, normalized tumor burden, and Ki-67 proliferation indices were quantified histologically. Hepatic *Cyp2e1* expression was measured to assess carcinogen bioactivation. Tumor mutations were analyzed by Sanger sequencing. RAS Q61R immunoreactivity and ERK phosphorylation were evaluated to assess oncogenic signaling. Bronchoalveolar lavage fluid cellularity was analyzed. Survival was monitored. Statistical analyses tested the main effects of chromosomal complement, gonadal sex, and their interaction. Results: Tumor multiplicity (*p* = 0.0729), tumor area (*p* = 0.5302), normalized tumor burden (*p* = 0.5316), and *Ki-67* indices (*p* = 0.6551) did not differ among genotypes. Hepatic *Cyp2e1* expression was comparable across groups (genotype *p* = 0.076; treatment *p* = 0.445). Sanger sequencing confirmed canonical Kras Q61R mutations. Anti-RAS (Q61R) immunohistochemistry revealed a significant genotype effect on mutant RAS expression (*F*(3,23) = 3.48, *p* = 0.032), with the highest H-scores observed in XYF mice compared with male gonadal genotypes; ERK phosphorylation did not differ. Bronchoalveolar lavage fluid analysis revealed increased lymphocytes after urethane exposure without genotype-dependent effects. Survival differed significantly, with XX females demonstrating prolonged survival relative to XY males. Conclusions: Sex influenced survival independently of tumor burden, indicating that sex-associated differences in lung cancer outcomes are likely driven by systemic or microenvironmental factors rather than tumor-intrinsic growth mechanisms.

## Simple Summary

Lung cancer affects men and women differently, but the biological reasons for these differences are not fully understood. Most research has focused on sex hormones, while the contribution of sex chromosomes has been more difficult to study. In this project, we used a specialized mouse model that separates the effects of sex chromosomes from those of gonadal hormones to better understand how each factor influences lung tumor development. By inducing lung tumors and analyzing tumor growth, tissue features, and immune responses, we examined whether chromosomal sex, hormonal sex, or both drives differences in disease progression. We found that sex influenced survival without affecting tumor burden, suggesting that sex differences in lung cancer outcomes may arise from systemic or microenvironmental factors rather than differences in tumor growth itself. These findings provide new insight into the biological basis of sex differences in lung cancer and may support the development of more personalized, sex-informed approaches to cancer prevention and treatment.

## 1. Introduction

Lung cancer remains the leading cause of cancer-related mortality in the United States, with non–small cell lung cancer (NSCLC) accounting for approximately 85% of newly diagnosed cases [1]. Although both overall lung cancer incidence and mortality have declined over the past decade, these trends differ substantially by sex [2,3]. Recent U.S. data reveal divergent patterns in incidence, progression, and survival between males and females, underscoring persistent sex-associated disparities in lung cancer outcomes [1]. Despite these well-established epidemiologic differences, the biological mechanisms driving sex-specific variation in lung cancer—particularly during tumor initiation and progression—remain poorly defined [2].

While tobacco smoking is the primary risk factor for lung cancer, a substantial proportion of cases occur in never-smokers, disproportionately affecting women [4,5]. Tumors arising in never-smokers often display distinct molecular characteristics and therapeutic responses, implicating sex-linked biological modifiers beyond tobacco exposure alone [6]. These observations highlight the need to identify intrinsic mechanisms that contribute to sex differences in lung tumorigenesis.

Experimental models have provided partial insight into these processes. In urethane-induced lung carcinogenesis, ovariectomy accelerates tumor growth and proliferation in susceptible mouse strains, whereas castration produces more variable effects, indicating a role for gonadal hormones in modulating tumor development. However, these effects are highly dependent on genetic background [7,8,9]. In relatively resistant strains such as C57BL/6, sex differences in tumor multiplicity and progression are modest or absent, suggesting that additional factors beyond circulating hormones may constrain malignant progression [10].

A major limitation of prior studies is that gonadal sex and sex chromosome complement are inherently confounded, preventing clear separation of endocrine effects from those mediated by XX versus XY gene dosage or their interaction. As a result, the relative contributions of sex chromosomes and gonadal sex to lung tumor initiation and progression remain unresolved. The Four Core Genotypes (FCG) mouse model overcomes this limitation by decoupling chromosomal sex from gonadal sex, enabling independent and factorial interrogation of these variables within a controlled genetic background. The FCG mouse model is generated by relocating the Sry gene (the primary testis-determining factor) from the Y chromosome to an autosome via transgenic insertion. This modification allows the gonadal phenotype (ovarian vs. testicular) to be determined by the presence or absence of the Sry transgene rather than by sex chromosomal complement, producing four distinct genotypes: XX mice with ovaries (XXF), XX mice with testes (XXM), XY mice with ovaries (XYF), and XY mice with testes (XYM). This design enables independent and factorial manipulation of chromosomal sex and gonadal sex within a single, controlled genetic background, allowing the attribution of phenotypic differences specifically to sex chromosome gene dosage, gonadal hormone exposure, or their interaction [11]. The FCG mouse model has been widely applied to dissect the independent contributions across diverse disease contexts, including pulmonary and systemic conditions such as allergic airway inflammation and asthma, immune and autoimmune disorders, as well as metabolic and cardiovascular diseases, where sex chromosome effects independently modulate physiology and disease susceptibility [12,13,14,15,16].

In this study, we used the FCG model combined with urethane-induced lung carcinogenesis to define the independent and interactive roles of sex chromosome complement and gonadal sex in lung tumor development and progression. We assessed survival, tumor burden, histopathology, proliferative activity, airway inflammation, oncogenic mutation status, MAPK pathway activation, and hepatic urethane bioactivation capacity across genotypes. Through this integrated approach, we investigated whether sex-associated differences in lung tumorigenesis arise from intrinsic chromosomal effects, gonadal hormone–dependent mechanisms, or downstream biological pathways that operate independently of inflammation, canonical MAPK signaling, or urethane bioactivation capacity.

## 2. Materials and Methods

### 2.1. Animals

Wild-type (WT) C57BL/6J (JAX, Strain# 000664, Bar Harbor, ME, USA) and FCG mice (JAX, Strain #010905, C57BL/6J background, Bar Harbor, ME, USA) were purchased from Jackson Laboratory (JAX) (Bar Harbor, ME), USA. Breeding pairs of female C57BL/6J with male FCG mouse (XYM) were created in our university vivarium. Pairs produced offspring that belong to one of the four genotypes (XXF, XXM, XYF, XYM). Genotypes were confirmed by genomic PCR, using the primers and protocol available at the JAX website for this strain, and further divided into control and experimental groups. Mice were housed in a 12 h light/dark cycle with standard chow diet and water ad libitum. Animals with ambiguous or failed genotyping results were excluded from all experimental groups. Criteria for exclusion from survival analyses are described in Section 2.3. Treatments were performed in accordance with the National Institute of Health guidelines on animal care and use of laboratory animals with prior protocol (# 23-006) approval from Indiana University Bloomington Institutional Animal Care and Use Committee (BIACUC). Sample size was determined a priori using a balanced 2 × 2 factorial design (*n* = 9 per genotype group), which estimated ≥90% power to detect a standardized effect size of Cohen’s d ≥ 0.8 on sex chromosome complement effects, based on data from pilot studies in wild-type C57BL/6 mice (α = 0.05).

### 2.2. Urethane-Induced LC Model

Urethane (Ethyl carbamate, Sigma-Aldrich, Cat# U2500, St. Louis, MO, USA) was dissolved in sterile PBS (100 mg/mL) and passed through a 0.22 µm sterile filter before administration. Age-matched (6–8 weeks) FCG mice were subjected to intraperitoneal urethane injections (1 mg/g BW) at a frequency of 1 injection per week for 10 consecutive weeks, followed by 20 weeks of tumor latency period as previously described [17]. Thirty weeks after the first injection and 72 h after the last injection, mice were sedated with an i.p. injection of a combination of ketamine (100 mg/kg of BW) and xylazine (10 mg/kg of BW) in 0.9% NaCl, followed by euthanasia via exsanguination. Mouse blood, lungs, liver, and bronchoalveolar lavage fluid (BALF) were collected. One batch of mouse lungs was snap frozen in liquid nitrogen and stored at −80 °C, while a separate group of lungs was fixed in 4% buffered formalin for 24 h, followed by a transfer into 70% histological grade ethanol.

### 2.3. Survival Analysis

Mice were monitored throughout the 30-week protocol for signs of morbidity in accordance with institutional animal care guidelines. Animals euthanized for reasons unrelated to urethane exposure (e.g., trauma, dermatologic lesions, dental abnormalities) were excluded from survival and cause-of-death analyses. Mice euthanized due to respiratory distress or mass lesions, as well as mice found dead, were considered mortality events. Gross examination was performed on mice that were euthanized prior to protocol endpoint, and deaths were classified as associated with pulmonary tumors and/or lymphoma-associated based on histological confirmation after tissue harvest. Mice found dead without confirmatory histology were treated as having an unknown cause of death. Survival analyses were performed using Kaplan–Meier methods with censoring at week 30, and genotype differences were assessed by the log-rank test [17].

### 2.4. BALF Analysis

Bronchoalveolar lavage fluid (BALF) was collected by performing a tracheotomy, cannulating the mouse through the incision, and inflating the mouse lungs with 1 mL of 1 mM EDTA/PBS solution (2.5 mL per mouse total). Mouse lungs were weighed after the BALF collection prior to snap-freezing. Collected BALF was spun at 1300 rpm for 10 min at 4 °C, and the supernatant was removed. The cell pellet was resuspended in 500 µL of 1 mM PBS/EDTA mix, and a 100 µL aliquot of the suspension was used for differential cell analysis. Total cell count was obtained by mixing 10 µL of resuspended cell pellet with 10 µL of 0.4% Trypan Blue stain (Invitrogen, Thermo Fisher Scientific, Watham, MA, USA), loading 10 µL of the mix on a Countess cell counting chamber slide, and running in an automated cell counter (Countess 3, Invitrogen, Thermo Fisher Scientific, Watham, MA, USA). A total of 5 × 10^5^ cells were suspended in 100 µL PBS, centrifuged onto slides in Cytospin tubes, air-dried, and subsequently stained with Hema 3 (Fisherbrand, Thermo Fisher Scientific, Watham, MA, USA). Twenty-four hours later, slides were mounted with cover glass using Cytoseal 60 (Epredia, Kalamzoo, MI, USA) mounting medium. Slides were scanned using the Motic EasyScan (Hong Kong, China) slide scanner and analyzed using QuPath v. 0.5.1 [18]. Differential cell counts were performed by manually analyzing at least 200 cells per sample as previously described [19].

### 2.5. Histological Analysis

Lung tissue stored in 70% ethanol was sent to Indiana University School of Medicine Histology Lab Service Core for paraffin embedding and sectioning. Fixed lungs were cut into 5 μm sections, mounted on microscopy slides, and stained with hematoxylin (Thermo Fisher Scientific, Cat# 7221, Watham, MA, USA) and eosin (Thermo Fisher Scientific, Cat# 7111, Watham, MA, USA) (H&E). Lung tissues were scanned with the Motic EasyScan slide scanner (Hong Kong, China), and tumor analysis was performed on QuPath v. 0.5.1. Histopathologic evaluation of lung lesions was on a representative hematoxylin and eosin (H&E)-stained section per lung, while quantitative tumor counting was conducted across five serial step sections per lung spaced at 200 μm intervals. Lesions were classified according to established criteria based on architectural and cytologic features. Adenomas were defined as well-demarcated, expansile lesions composed of relatively uniform epithelial cells lining preserved or mildly distorted alveolar structures, with minimal cytologic atypia and no evidence of invasion [20,21]. In contrast, adenocarcinomas were identified by loss of normal alveolar architecture, increased cellular pleomorphism and nuclear atypia, and evidence of invasive growth into surrounding lung parenchyma, including stromal, vascular, or pleural involvement when present. Histopathologic classification was performed in a blinded manner by three independent investigators. For tumor multiplicity assessment, lesions were enumerated across all five step sections. To avoid duplicate counting of the same lesion appearing in adjacent sections, tumors were matched based on anatomical location within the lung, maximal cross-sectional diameter, and centroid position, and only unique lesions were included in final counts. Tumor areas were estimated by annotating the lesion area in QuPath and were reported as a ratio of tumor area to the whole lung cross-sectional area.

### 2.6. Immunohistochemistry Staining and Analysis

Formalin-fixed, paraffin-embedded mouse lung sections were processed for immunohistochemistry (IHC) using a standardized protocol optimized for Ki-67 (Cell Signaling Technology, Cat# 12202, Danvers, MA, USA), anti-RAS (Q61R) (Abcam, Cat# ab227658, Cambridge, UK), and phospho-ERK (Invitrogen, Cat# 44-680G, Waltham, MA, USA). Slides (5 µm thickness) were heated on a shaker for 20 min at 65 °C, followed by deparaffinization in Histo-Clear (Electron Microscopy Sciences, Cat# 64110-04, Hatfield, PA, USA) and rehydration in graded ethanol solutions (100%, 90%, 80%) and water. The next step involved a heat-induced antigen retrieval in Tris-EDTA buffer (pH 9.0) for 25 min at 95 °C using microwave heating. Endogenous peroxidase activity was quenched with 3% hydrogen peroxide (Sigma Aldrich, Cat# 1072090250, St. Louis, MO, USA), and tissue permeability was enhanced with 0.3% Triton X-100 solution (Abcam, Cat# ab286840, Cambridge, UK) (15 min) prior to blocking with normal goat serum-based (Abcam, Cat# ab7481, Cambridge, UK) blocking solution for 30 min. Sections were incubated overnight (12 h) at 4 °C with primary antibody against anti-RAS (Q61R) (1:60), Ki-67(1:300), and p-ERK (1:300) diluted in antibody diluent (Abcam, Cat# ab64211, Cambridge, UK). Following washes in Tris-buffered saline containing Tween-20 solution (Abcam, Cat# ab286841, Cambridge, UK), sections were incubated with horseradish peroxidase–conjugated secondary antibody, goat anti-rabbit (1:500 dilution, Abcam, Cat# ab6721, Cambridge, UK), and immunoreactivity was visualized using 3,3′-diaminobenzidine (DAB) substrate (Abcam, Cat# ab64238, Cambridge, UK). Slides were counterstained with Mayer’s hematoxylin (Abcam, Cat# ab220365, Cambridge, UK), dehydrated in ethanol of increasing percentage (80%, 90%, 100%) and xylene, cleared, and permanently mounted with Cytoseal 60 (Epredia, USA, Kalamazoo, MI, USA). All incubation times, reagent volumes, and stain development times were kept consistent across experimental groups to ensure comparability.

Quantitative analysis of IHC staining was performed using QuPath image analysis software v.0.5.1. For anti-RAS (Q61R) and p-ERK staining, immunoreactivity was quantified using an H-score–based approach, integrating both staining intensity and the proportion of positively stained cells within annotated tumor regions. Tumor areas were manually delineated on H&E-matched sections, and staining intensity thresholds were visually validated on representative slides and then applied uniformly across all samples to classify cells as negative, weak, moderate, or strong. H-scores were calculated as the sum of the percentage of cells at each intensity level multiplied by the corresponding intensity score (H-score = [% Cells_1_ × 1] + [% Cells_2_ × 2] + [% Cells_3_ × 3], range 0–300), yielding a composite measure of protein expression [22]. Automated nuclear detection was performed in QuPath using consistent detection parameters, and Ki-67–positive cells were expressed as counts normalized to the analyzed tumor area (cells/mm^2^) to quantify proliferative activity. All image analyses were conducted using identical thresholding and detection settings across experimental groups and were performed blinded to genotype and treatment to ensure consistency and minimize bias.

### 2.7. qPCR Analysis

Tissue was collected and mechanically homogenized prior to total RNA isolation using the Direct-zol RNA Miniprep kit (Zymo Research Corporation, Cat# R2052, Irvine, CA, USA). The RNA yield and purity were assessed spectrophotometrically using a NanoDrop ND-2000 instrument (Thermo Scientific, Wilmington, DE, USA, Waltham, MA, USA). A set of liver RNA samples was used to perform reverse transcription and qPCR (*n* = 6) using a mouse *Cyp2e1* Taqman probe (Thermo Fisher Scientific, Cat# 4331182, Assay ID Mm00491127_m1, Waltham, MA, USA) to estimate the expression levels of liver enzyme *Cyp2e1*. The *18s* (TaqMan Assay ID: Mm03928990_g1, Waltham, MA, USA) was used as the endogenous reference gene for normalization. Relative *Cyp2e1* expression was calculated using the 2^−^^ΔΔCt^ method The amplification reaction was run on QuantStudio 5 (Thermo Fisher Scientific, Waltham, MA, USA), and the fold changes were calculated from Ct values using the Delta-Delta Ct method (2^−ΔΔCt^) [23].

### 2.8. Sanger Sequencing

Tumor and healthy lung tissue were collected, mechanically homogenized with consequent DNA isolation using the Quick-DNA Miniprep Plus kit (Zymo Research Corporation, Cat# D4069, Irvine, CA, USA). *Kras* primers were designed using the online tool Primer-BLAST (https://www.ncbi.nlm.nih.gov/tools/primer-blast/, accessed on 12 November 2025) by the National Center for Biotechnology Information (NCBI). Designed primers (forward primer: GACTCCTACAGGAAACAAGTAGTAA, reverse primer: GAAAGCCCTCCCCAGTTC) were ordered from Thermo Fisher Scientific (Cat# 10336022, Waltham, MA, USA). The PCR using EZ PCR Master mix (MiniPCR, Cat# RG-1000-01, Cambridge, MA, USA) was run on the tumor and lung tissue DNA as described in Supplementary Methods S1, and the reaction product was sent to a third-party laboratory (Eurofins Genomics Services, Louisville, KY, USA) for Sanger sequencing to confirm the mutations on codon 61 in the *Kras* gene. The sequencing data were visualized and analyzed using SnapGene software v. 8.2.2 (Siemens, Boston, MA, USA).

### 2.9. Statistical Analysis

Survival outcomes were analyzed using Kaplan–Meier curves with censoring at week 30, and differences between groups were assessed by the log-rank test. For continuous outcomes, including normalized lung weight, tumor multiplicity, tumor area ratios (total tumor area normalized to total lung area), BALF cellular endpoints, and quantitative immunohistochemical measures, linear models were used to evaluate the effects of treatment (urethane vs. PBS), sex chromosome complement (XX vs. XY), gonadal sex (female vs. male), and their interactions. Tumor multiplicity was analyzed in much the same way, but using Poisson instead of linear regression, as it is a count variable and so not normally distributed. Longitudinal body weight measurements were analyzed using linear mixed models with a random intercept for subject ID to account for repeated measurements within individuals and the factorial experimental design. Body weight was modeled as a function of time, treatment (urethane vs. PBS), sex chromosome complement (XX vs. XY), gonadal sex (female vs. male), and all possible interactions. This model was also adjusted for body weight at baseline. Type three sum of squares using Satterthwaite’s degrees of freedom estimate were used to assess the statistical significance of the main effects and interactions between time, treatment, chromosomal sex, and gonadal sex, followed by model-based estimated marginal means (EMMs) and 95% confidence intervals to facilitate interpretation of inter-group differences. Pairwise contrasts were performed on EMMs with adjustment for multiple comparisons. Data are presented as mean ± SEM unless otherwise indicated. All statistical tests were two-sided, and statistical significance was defined as *p* < 0.05. Statistical annotations in figures reflect model-based, adjusted analyses; nonparametric descriptive tests are reported in text only. Statistical analyses were performed in consultation with the Indiana University Biostatistics Consulting Center, and the complete modeling workflow is documented in the accompanying analysis report (Supplementary Data File S1).

## 3. Results

Sex chromosome complement and gonadal sex influence survival and body weight following urethane exposure.

**Figure 1:**
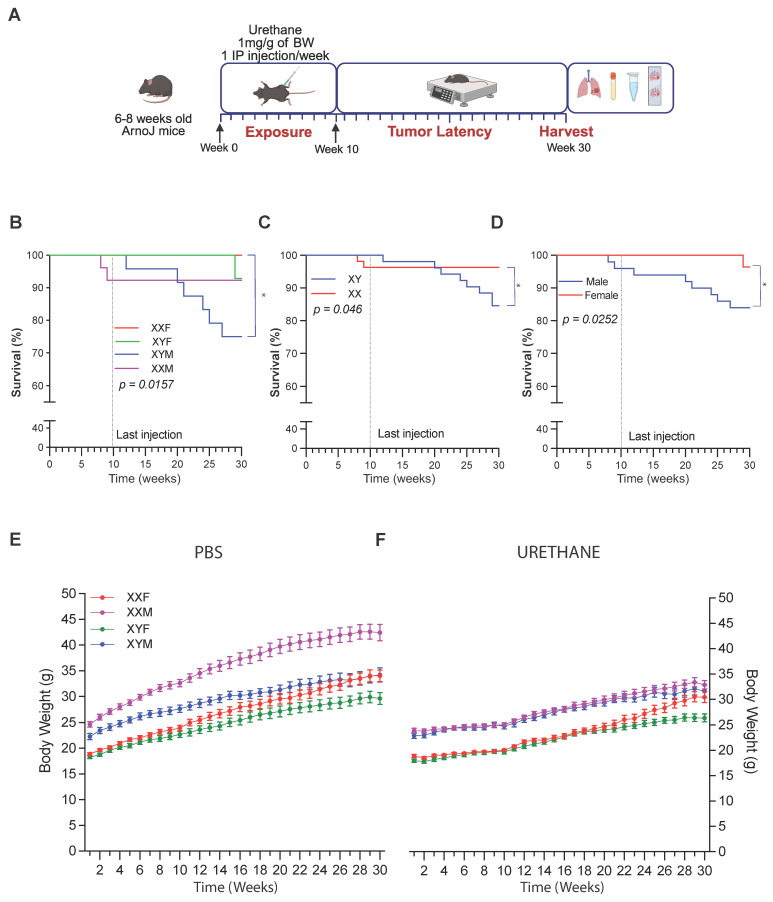
Mouse survival and body weights. (**A**) Schematic overview of the experimental protocol. The FCG mice (*n* = 9–10 per group) were injected intraperitoneally with urethane (1 mg/g body weight) or PBS (vehicle) once weekly for 10 weeks, followed by a 20-week tumor latency period and tissue harvest at week 30. Created in BioRender (Maksat Babayev, 2026). https://BioRender.com/euqd81y, (accessed on 15 February 2026). (**B**–**D**) Kaplan–Meier survival curves for urethane-treated mice stratified by genotype. (**B**), sex chromosome complement (XX vs. XY; (**C**)), and gonadal sex (female vs. male; (**D**)). Survival differences were assessed using log-rank tests, with *p* values indicated on each panel. Animals were censored at week 30 if alive at study termination. (**E**,**F**) Longitudinal body weight trajectories for PBS-treated (**E**) and urethane-treated (**F**) mice over the 30-week protocol. Points represent observed group means ± SEM. Statistical comparisons are based on model-derived estimated marginal means (Supplementary Data File S1). (* *p* < 0.05).

Kaplan–Meier analyses demonstrated significant differences in survival among urethane-treated FCG mice when stratified by genotype, sex chromosome complement, and gonadal sex (Figure 1B–D). When grouped by genotype and analyzed, XYM mice exhibited significantly reduced survival compared with XXF mice (log-rank *p* = 0.0157), indicating increased mortality associated with the combined presence of the Y chromosome and male gonads (Figure 1A). Stratification by chromosomal sex revealed poorer survival in XY compared with XX mice (log-rank *p* = 0.046; Figure 1C), while grouping by gonadal sex showed significantly reduced survival in gonadal males relative to gonadal females (log-rank *p* = 0.0252; Figure 1D). These findings indicate that both sex chromosome complement and gonadal sex independently contribute to survival outcomes following urethane-induced lung tumorigenesis, with the most pronounced vulnerability observed in XYM mice.

Because urethane is a multisite carcinogen, survival outcomes reflect combined pulmonary and extrapulmonary disease burden. A subset of mice required euthanasia due to respiratory distress. Euthanasia, followed by gross morphological examination of the thoracic cavity, revealed the presence of lymphoid masses in addition to pulmonary tumors. Histological analysis of masses was performed, and representative images are available (Supplementary Figure S1). These cases were included in overall survival analyses.

Body weight trajectories were analyzed using a linear mixed-effects model with fixed effects of week, genotype, treatment, and baseline weight. As expected, body weight increased over time in all groups (week effect, *p* < 0.0001). Urethane-treated mice exhibited significantly lower body weights compared with PBS controls across the study period (treatment effect, *p* < 0.0001). Genotype influenced weight trajectories (*p* = 0.011), and genotype-by-treatment interactions were observed (*p* = 0.0002), indicating modest divergence in weight gain patterns among genotypes. However, no genotype demonstrated marked weight loss or cachexia, and overall growth patterns remained consistent with expected age-related increases (Figure 1E,F).

### 3.1. Genotype-Dependent Differences in Lung Tumor Burden and Histopathologic Features

**Figure 2:**
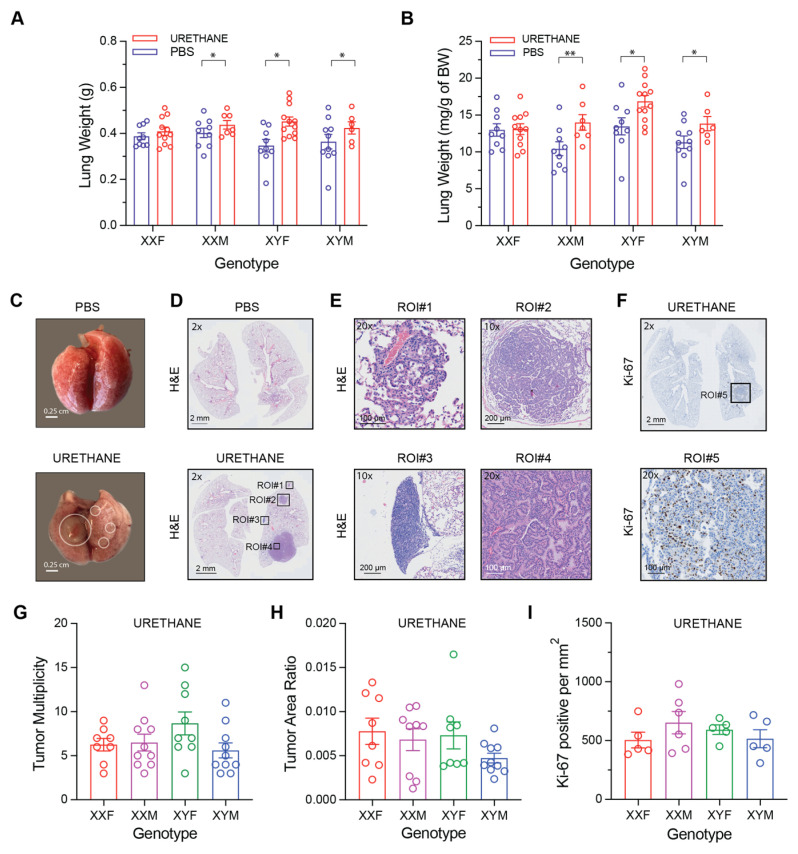
Tumor phenotype and burden in urethane-induced lung carcinogenesis in the FCG mouse model. (**A**) Lung weights measured at harvest. (**B**) Lung weights normalized to body weight (BW). Urethane significantly increased lung weight (two-way ANOVA main effect of treatment). (**C**) Representative whole-lung images (dorsal view) from PBS- and urethane-treated mice. Urethane-treated lungs exhibit multiple surface nodules consistent with tumor formation; tumors are highlighted with white circles. Scale bars, 0.25 cm. (**D**) Representative hematoxylin and eosin (H&E)–stained lung sections from PBS- and urethane-treated mice illustrating normal lung architecture in controls and neoplastic lesions following urethane exposure. Scale bars, 2 mm (2× magnification). (**E**) Enlarged regions of interest (ROIs) highlighting histopathologic features of urethane-induced lung lesions: focal adenomatous (alveolar) hyperplasia (ROI#1, scale bar, 100 μm, 20× magnification), papillary/solid adenoma (ROI#2, scale bar, 200 μm, 10× magnification), mononuclear inflammatory aggregate (ROI#3, scale bar, 200 μm, 10× magnification), and papillary adenocarcinoma (ROI#4, scale bar, 100 μm 20× magnification). (**F**) Representative immunohistochemical staining for Ki-67 in urethane-induced lung adenocarcinoma. The top panel shows a whole-lung section (scale bar, 2 mm, 2× magnification); the bottom panel shows a magnified ROI (ROI#5; scale bar, 100 μm, 20× magnification). (**G**) Tumor multiplicity, defined as the number of discrete lung lesions per mouse, was quantified from serial H&E-stained sections. (**H**) Tumor area ratios, calculated as the summed lesion surface area per mouse normalized to the total lung cross-sectional area. (**I**) Ki-67 proliferation index expressed as Ki-67–positive cells per mm^2^ of tumor area. One value per mouse was obtained by dividing the total number of Ki-67–positive cells by the summed tumor area. For (**G**–**I**), statistical analyses were performed using linear modeling approaches with model-based estimated marginal means and contrasts, as detailed in the accompanying statistical analysis report. Data are presented as mean ± SEM unless otherwise indicated. (* *p*< 0.05, ** *p* < 0.01).

Lung weight was analyzed using a two-way linear model with genotype, treatment, and their interaction as fixed effects. For unnormalized lung weight (g), there was no significant genotype × treatment interaction (*F*(3,67) = 0.91, *p* = 0.44) and no main effect of genotype (*F*(3,67) = 1.14, *p* = 0.34). However, urethane treatment significantly increased lung weight overall (*F*(1,67) = 13.80, *p* = 0.0004). Within-genotype comparisons indicated higher lung weights in urethane-treated mice relative to PBS controls in XXM (*p* = 0.0457), XYF (*p* = 0.0116), and XYM (*p* = 0.0235), whereas the increase in XXF did not reach statistical significance (*p* = 0.5983) (Figure 2A).

Analysis of normalized lung weight (mg/g body weight) similarly demonstrated no significant genotype × treatment interaction and no main effect of genotype. A significant treatment effect was observed, with urethane increasing normalized lung weight. Within-genotype contrasts showed significant increases in XXM (*p* = 0.0031), XYF (*p* = 0.0325), and XYM (*p* = 0.0100), but not in XXF (*p* = 0.9430) (Figure 2B).

Together, these findings indicate that urethane exposure increased lung mass independent of sex chromosome complement, with treatment effects observed across most genotypes and no evidence of genotype-specific modulation.

Gross examination of lungs at harvest revealed marked differences between PBS- and urethane-treated mice. Lungs from PBS-treated animals appeared uniformly aerated with smooth pleural surfaces and no visible lesions (Figure 2C top), whereas urethane-treated mice exhibited multiple surface nodules of variable size distributed throughout the lung lobes, consistent with extensive tumor burden (Figure 2C bottom). These gross changes reflect tumor-associated tissue expansion and parenchymal remodeling across genotypes. Histologic evaluation of lungs revealed that urethane exposure resulted in 100% tumor incidence across all genotypes, whereas no tumors were observed in PBS-treated controls. All urethane-treated mice developed lung adenomas, indicating that tumor initiation following urethane exposure was not influenced by sex chromosome complement or gonadal sex. In contrast, progression to adenocarcinoma occurred in only a subset of animals and varied by genotype. Adenocarcinoma incidence was lowest in XXM (10.0%, 1/10) and XXF (12.5%, 1/8) mice, intermediate in XYM mice (20.0%, 2/10), and highest in XYF mice (33.3%, 3/9) (Table 1).

Histologic evaluation of lung sections revealed discrete, well-demarcated nodular lesions in urethane-treated mice consistent with pulmonary adenomas (Figure 2D,E). On H&E staining, these lesions were characterized by expansile growth patterns with crowding of epithelial cells and distortion of normal alveolar architecture. Higher-magnification images demonstrated increased cellular density with relatively uniform tumor cells forming solid and papillary structures. Histologic examination confirmed the presence of urethane-induced proliferative lung lesions, including focal adenomatous hyperplasia, pulmonary adenomas, and adenocarcinomas (Figure 2E). In contrast, lungs from PBS-treated controls maintained preserved alveolar architecture without focal proliferative lesions.

Tumor cell proliferative activity was assessed by quantifying Ki-67–positive cells normalized to tumor area (Ki-67^+^ cells per mm^2^ of tumor). Ki-67 indices were analyzed using a linear model with genotype (XXF, XXM, XYF, XYM) as the predictor, and statistical significance was evaluated by Type II ANOVA. Genotype did not significantly influence Ki-67 staining, indicating no detectable differences in tumor cell proliferation among the Four Core Genotypes at the study endpoint. Model-based estimated marginal means showed overlapping Ki-67 indices across genotypes, and no adjusted pairwise comparisons reached statistical significance. These findings suggest that genotype-dependent differences in tumor burden or survival observed in this model are unlikely to be explained by large differences in tumor proliferative activity as measured by Ki-67 (Figure 2F,I,J).

Tumor multiplicity was analyzed using a Poisson regression model with genotype as the predictor, and overall genotype effects were evaluated using a Type II analysis of deviance. Genotype did not significantly influence tumor multiplicity (LR χ^2^ = 6.97, df = 3, *p* = 0.073), indicating no statistically significant genotype-dependent differences in the number of urethane-induced lung lesions per mouse (Figure 2G). Model-based estimated marginal means revealed broadly comparable tumor counts across genotypes; however, XYF mice exhibited numerically higher lesion counts than XYM mice, a difference that did not reach statistical significance in adjusted pairwise comparisons. Together, these findings indicate that urethane-induced tumor initiation is largely similar across the four core genotypes, with any genotype-related effects on tumor number being modest and hypothesis-generating rather than definitive.

Overall tumor burden was assessed by calculating the tumor area ratio, defined as the summed tumor area per mouse normalized to total lung area (Figure 2H). Tumor area ratios were analyzed using a linear model with genotype as the predictor, and statistical significance was evaluated by Type II ANOVA. Genotype did not significantly influence tumor area ratio, indicating no strong evidence for genotype-dependent differences in total tumor burden. Model-based estimated marginal means were comparable across genotypes, and adjusted pairwise comparisons did not reveal statistically significant differences. These findings indicate that, although urethane exposure induces substantial tumor burden, gross differences in tumor area are not driven by genotype in the Four Core Genotypes model.

Urethane induces pulmonary inflammation without genotype-specific modulation of airway immune cellularity.

**Figure 3:**
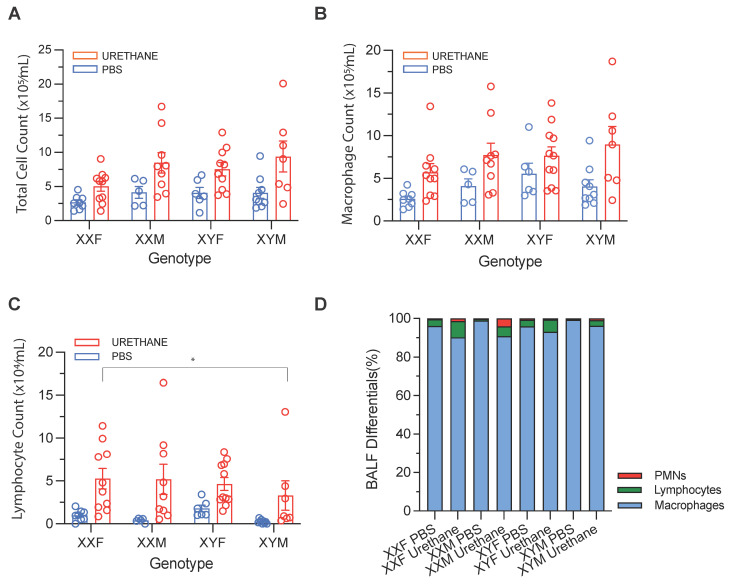
BALF cellularity following urethane exposure. (**A**) Total BALF cell counts (×10^5^ cells/mL) in PBS- and urethane-treated mice across Four Core Genotypes (XXF, XXM, XYF, XYM). (**B**) Absolute BALF macrophage counts (×10^5^ cells/mL) shown by genotype and treatment. (**C**) Absolute BALF lymphocyte counts (×10^4^ cells/mL) shown by genotype and treatment; (**D**) Relative composition of BALF immune-cell populations expressed as percentages of total BALF cells, including macrophages, lymphocytes, and polymorphonuclear leukocytes (PMNs; neutrophils + eosinophils). Statistical analyses were performed using linear models as described in Methods; no genotype × treatment interaction was detected for BALF endpoints. Results indicate that urethane exposure induces a robust pulmonary inflammatory response characterized by increased BALF cellularity and lymphocyte accumulation, with no evidence of genotype-specific modulation of the treatment response. For (**A**–**C**), each symbol represents an individual mouse; bars indicate observed means ± SEM. (* *p* < 0.05).

Bronchoalveolar lavage fluid (BALF) analyses demonstrated that urethane treatment significantly increased total BALF cellularity. In stratified nonparametric comparisons, total cells/mL were elevated in urethane-treated XXF (*p* = 0.006) and XYM (*p* = 0.042) mice, with similar directional increases observed in other genotypes (Figure 3A). Absolute macrophage counts were likewise increased in urethane-treated XXF (*p* = 0.009) and XYM (*p* = 0.042) animals, although no overall genotype effect was detected in descriptive comparisons (Figure 3B). In linear models adjusting for total BALF cellularity, urethane was associated with reduced total macrophage counts conditional on total cells/mL (treatment effect *p* = 0.011), with no significant genotype main effect (*p* = 0.091) and no genotype × treatment interaction (*p* = 0.399 (Figure 3D)). These findings indicate that although total macrophage counts increased in absolute terms following urethane exposure, their relative representation decreased when accounting for overall inflammatory burden. Lymphocyte counts demonstrated significant genotype (*p* = 0.0449) and treatment (*p* = 0.0256) main effects in adjusted models, without evidence of interaction (*p* = 0.546). Tukey-adjusted comparisons revealed reduced lymphocyte counts in XYM relative to XXF mice (*p* = 0.023) (Figure 3C). No consistent genotype-dependent differences were observed for polymorphonuclear leukocytes (PMNs).

These findings indicate that urethane exposure promotes lymphocyte accumulation in the airways and that modest genotype-associated differences in lymphocyte levels exist at baseline; however, the absence of interaction effects suggests that genotype does not differentially modify the lymphocytic response to urethane. Collectively, the BALF data support a model in which urethane induces a sustained inflammatory milieu, while genotype-related differences in tumor phenotype are unlikely to be driven by large, treatment-specific changes in airway immune-cell composition.

### 3.2. Mutant RAS Immunoreactivity

**Figure 4:**
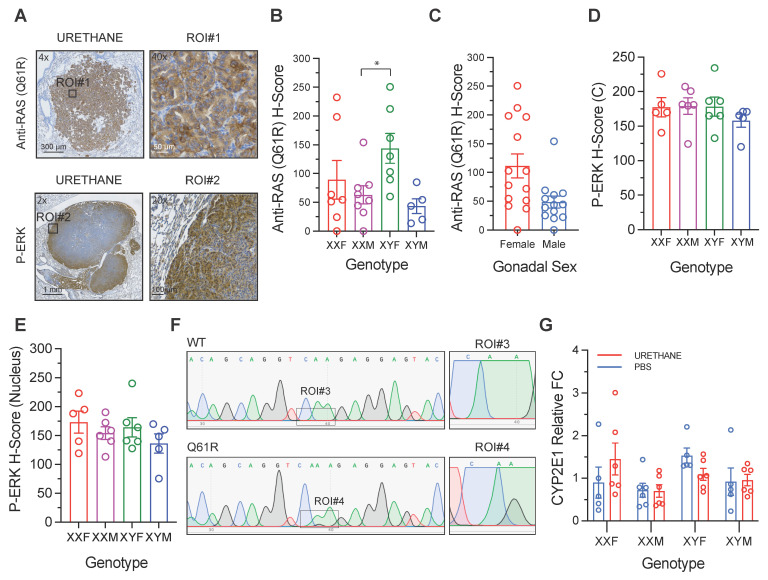
Mutant RAS expression, MAPK pathway activation, and hepatic urethane bioactivation capacity in the FCG model. (**A**) Representative immunohistochemical staining of urethane-induced lung tumor for mutant RAS (Q61R) (top) and phosphorylated ERK (p-ERK) (bottom). Top left, anti-RAS (Q61R) staining; top right, magnified region of interest (ROI#1) from the anti-RAS–stained tumor. Bottom left, p-ERK staining; bottom right, magnified region of interest (ROI#2) from the p-ERK–stained tumor. Scale bars, 300 μm (4× magnification), 50 μm (anti-RAS ROI#1, 40× magnification), 1 mm (2× magnification), and 100 μm (p-ERK ROI#2, 20× magnification). (**B**) Anti-RAS (Q61R) H-scores grouped by genotype (XXF, XXM, XYF, XYM). H-scores were calculated as the sum of the percentage of tumor cells at each staining intensity multiplied by the corresponding intensity score (1× weak, 2× moderate, 3× strong). (**C**) Anti-RAS (Q61R) H-scores grouped by gonadal sex (Female vs. Male), shown to illustrate sex-associated differences in mutant RAS immunoreactivity independent of genotype. (**D**) Cytoplasmic p-ERK H-scores across genotypes. (**E**) Nuclear p-ERK H-scores across genotypes, reflecting transcriptionally active MAPK signaling. (**F**) Representative Sanger sequencing chromatograms confirming the presence of *Kras* codon 61 mutations in urethane-induced lung tumors. (**G**) Hepatic *Cyp2e1* mRNA expression measured by quantitative PCR, shown by treatment and genotype as an indicator of urethane bioactivation capacity (FC: Fold Change). For (**B**–**E**) each symbol represents an individual mouse; bars denote observed means ± SEM. (* *p* < 0.05).

Quantitative analysis of anti-RAS (Q61R) immunohistochemistry(Figure 4A) revealed a significant overall effect of genotype on mutant RAS protein expression ((3,23)_3_ = 3.48, *p* = 0.032). Examination of the estimated marginal means indicates a clear pattern in which the genotype containing XY chromosomes with female gonads (XYF) displayed the highest mutant RAS expression (EMM = 139.9 ± 22.9), whereas genotypes with male gonads exhibited lower levels overall. Specifically, the XXM (EMM = 52.6 ± 21.4) and XYM (EMM = 41.9 ± 27.1) groups showed the lowest mean H-scores, while XXF mice (EMM = 82.9 ± 22.9) demonstrated intermediate expression (Figure 4B).

Post hoc comparisons indicated that mutant RAS expression was significantly higher in XYF mice compared with XXM mice (Difference = −87.3 ± 31.4, (23)_3_ = −2.78, *p* = 0.048). A similar magnitude difference was observed between XYF and XYM animals (Difference = 98.8 ± 35.5), although this comparison narrowly missed statistical significance after Tukey adjustment (*p* = 0.051). No other genotype comparisons were statistically significant, including the comparison between the two male gonadal groups (XXM vs. XYM; *p* = 0.99).

### 3.3. MAPK Pathway Activation

Quantitative analysis of phosphorylated ERK (P-ERK) immunohistochemistry did not reveal genotype-dependent differences in either cytoplasmic or nuclear staining. For cytoplasmic P-ERK H-scores, linear model analysis showed no significant effect of genotype ((3,18)_8_ = 0.6178, *p* = 0.6124). Estimated marginal means were comparable across genotypes, with values of 177 ± 13.2 for XXF (95% CI: 150–205), 179 ± 12.0 for XXM (95% CI: 154–204), 178 ± 12.0 for XYF (95% CI: 153–204), and 158 ± 13.2 for XYM (95% CI: 130–186). Tukey-adjusted pairwise comparisons confirmed the absence of significant differences between genotypes (all *p* ≥ 0.6457) (Figure 4D). Similarly, nuclear P-ERK H-scores did not differ significantly among groups ((3,18)_8_ = 0.9285, *p* = 0.4472). Estimated marginal means were 173 ± 16.6 for XXF (95% CI: 138–208), 154 ± 15.1 for XXM (95% CI: 122–186), 164 ± 15.1 for XYF (95% CI: 132–196), and 136 ± 16.6 for XYM (95% CI: 101–171). Post hoc comparisons using Tukey adjustment likewise showed no statistically significant pairwise differences (all *p* ≥ 0.4157) (Figure 4E). Collectively, these findings indicate that ERK pathway activation, assessed in both cytoplasmic and nuclear compartments, is not significantly modulated by sex chromosome complement or gonadal sex in urethane-induced lung adenomas.

### 3.4. Hepatic Cyp2e11 Expression and Urethane Bioactivation Capacity

Liver *Cyp2e1* expression was evaluated by quantitative PCR, and statistical analyses were performed on ΔCt values to satisfy model assumptions. Two-way ANOVA revealed no significant interaction between genotype and treatment (F(3,37) = 1.61, *p* = 0.203), and no significant main effect of treatment (F(1,37) = 0.60, *p* = 0.445). Although the main effect of genotype approached statistical significance in the full model (F(3,37) = 2.48, *p* = 0.076), this trend did not reach conventional significance thresholds and remained non-significant in the reduced model (*p* = 0.085) (Figure 4G). These findings indicate that hepatic *Cyp2e1* mRNA expression does not differ significantly as a function of sex chromosome complement or urethane treatment in this cohort. Collectively, the data suggest that baseline hepatic metabolism of urethane via CYP2E1 1 is not sexually dimorphic in this model and is unlikely to account for downstream sex-associated differences in tumor development or signaling outcomes.

## 4. Discussion

Lung cancer remains the leading cause of cancer-related mortality worldwide, and accumulating clinical evidence demonstrates that biological sex significantly influences disease risk, tumor characteristics, and therapeutic outcomes. Epidemiologic studies have reported differences between men and women in incidence patterns, susceptibility to tobacco carcinogens, prevalence of specific driver mutations, immune responses, and survival following targeted and immunotherapies [2,24,25,26]. Despite these well-documented disparities, the biological basis of sex differences in lung cancer remains incompletely understood. In human populations, chromosomal sex and lifelong exposure to sex hormones are inherently linked, making it difficult to determine whether observed differences arise from intrinsic sex chromosome effects, hormonal regulation, or their interaction. Disentangling these mechanisms is clinically important, as it may refine risk assessment, improve interpretation of molecular biomarkers, and inform sex-informed therapeutic strategies. Experimental systems that separate chromosomal and hormonal influences are therefore essential to define the causal drivers of sex-associated variation in lung tumorigenesis.

Using the FCG mouse model in the context of urethane-induced lung carcinogenesis, we sought to disentangle the independent contributions of sex chromosome complement and gonadal sex to tumor development and progression. In this study, separation of sex chromosome complement from gonadal sex using the FCG model revealed no meaningful differences in tumor multiplicity, tumor size, normalized tumor burden, or proliferative index among genotypes. Although numerical variation was present, overall tumor burden was comparable across genotypes. These findings indicate that, within the urethane-induced lung cancer model, neither chromosomal sex nor gonadal sex exerts a dominant influence on tumor growth or proliferation. Thus, any sex-associated differences observed in downstream molecular signaling or survival outcomes are unlikely to be driven by differences in baseline tumor burden. Instead, these results suggest that sex effects appear to operate through qualitative alterations in tumor biology rather than through changes in tumor quantity or growth rate.

Survival analysis revealed a significant advantage in the XXF group compared with the XYM group, indicating that biological sex influences overall disease trajectory in this model. However, this difference in survival occurred in the absence of measurable differences in lung tumor multiplicity, normalized tumor burden, or proliferative index across genotypes. Notably, lymphomas were observed in a subset of mice euthanized prior to protocol completion, indicating that urethane exposure in this cohort was not limited to pulmonary tumorigenesis. Analysis of bronchoalveolar lavage fluid demonstrated treatment-associated increases in lymphocyte counts without strong genotype-dependent shifts in proportional composition. Although XXF mice had significantly higher absolute counts of lymphocytes, compared to XYM, with a significant genotype effect overall. Sanger sequencing confirmed that urethane-induced lung tumors harbored the canonical Kras Q61R mutation, the initiating event, validating the molecular fidelity of the model. Although genotype showed to be a significant source of variation in mutant RAS (Q61R) immunoreactivity, with XYF tumors exhibiting the highest H-scores and a significant difference relative to XXM tumors, downstream MAPK pathway activation, assessed by cytoplasmic and nuclear P-ERK staining, did not differ between groups. Hepatic expression of Cyp2e1, the primary enzyme responsible for urethane bioactivation, was comparable across genotypes, suggesting that differential carcinogen metabolism does not account for the survival disparity. Collectively, these findings indicate that sex-associated differences in survival occur independently of lung tumor burden, canonical ERK pathway activation, or hepatic carcinogen metabolism and may reflect broader systemic effects of carcinogen exposure.

Mechanistically, these data suggest that sex influences disease progression through qualitative differences in tumor biology and host response rather than differences in lung tumor quantity alone. The absence of genotype effects on tumor burden implies that tumor initiation and early growth are not strongly sex-dependent in this system. Instead, survival differences may arise from sex-specific modulation of tumor–host interactions, immune regulation, or non-canonical KRAS signaling pathways. The dissociation between mutant RAS immunoreactivity and ERK activation further supports the possibility that sex affects signaling network dynamics downstream or parallel to KRAS, rather than simply altering proliferative output. The occurrence of lymphomas in early euthanized animals raises the additional possibility that systemic hematologic malignancies or altered immune homeostasis contributed to survival outcomes, potentially in a sex-dependent manner. Together, these findings underscore the complexity of sex-associated influences in carcinogen-induced models and highlight the need to consider both tumor-intrinsic and systemic host factors when interpreting survival differences.

Repeated urethane administration in C57BL/6 mice induces robust pulmonary inflammation that precedes overt tumor formation. Xu et al. reported that ten weekly injections (1 g/kg) produced 100% tumor incidence by week 28 and were associated with increased BALF macrophages, neutrophils, lymphocytes, elevated pro-inflammatory cytokines (e.g., IL-6, RANTES), and progressive NF-κB activation [27]. In the urethane model, tumor initiation is driven by activating Kras Q61R mutations that signal through the MAPK pathway, whereas NF-κB primarily functions as an inflammatory promoter linking chronic airway inflammation to tumor survival and progression [28,29]. Our observed airway inflammatory changes are consistent with this established framework. By incorporating the Four Core Genotypes model, the present study extends these findings by testing whether sex chromosome complement and gonadal sex modify either the oncogenic KRAS–MAPK axis or the inflammatory NF-κB–associated microenvironment in urethane-induced lung tumorigenesis. Sex effects in urethane-induced lung tumorigenesis appear to be highly strain- and exposure-dependent; in CC57BR/Mv mice, males exhibited substantially greater urethane-induced lung adenoma sensitivity than females after single-dose urethane, and this difference was strongly modified by early-life androgen exposure, suggesting an important role for developmental hormone effects rather than adult hormone levels alone [8]. Evidence for sex effects on tumor progression markers in urethane models is mixed and can be highly dependent on strain and exposure protocol. In BALB/c mice treated with urethane (10 mg/mouse IP twice weekly × 5; total 50 mg) and followed for 6 months with histologic separation of adenomas versus adenocarcinomas, Ki-67 proliferation indices differed by sex and gonadal status, despite limited sex differences in tumor incidence/count outcomes in that system. This contrasts with our C57BL/6-background FCG protocol (1 g/kg IP once weekly × 10; 20-week latency), where Ki-67 density did not differ across genotypes, suggesting that sex-linked proliferative programs may be contingent on genetic background, dosing schedule, and/or the degree of malignant progression captured at endpoint [7].

Increasing evidence indicates that sex hormones contribute to lung carcinogenesis through receptor-mediated signaling pathways that intersect with oncogenic drivers and inflammatory cascades [30,31,32]. Estrogen signaling has been shown to causally modulate KRAS-driven lung tumor progression in vivo. In a conditional Kras-driven lung adenocarcinoma model with concurrent p53 loss, estrogen promoted tumor growth, and endocrine manipulation (ovariectomy or estradiol supplementation) significantly altered tumor burden, demonstrating that estrogen can enhance KRAS-initiated tumor progression [33]. Beyond tumor cell–intrinsic proliferation, estrogen signaling has also been linked to KRAS-associated inflammatory pathways, including STAT3 and NF-κB, influencing tumor–immune interactions within the microenvironment [34]. Together, these studies indicate that estrogen does not initiate KRAS mutation but can potentiate KRAS-mutant lung cancer progression through coordinated modulation of MAPK and inflammatory signaling axes.

In contrast, our urethane-driven model on a C57BL/6 background did not demonstrate genotype-associated differences in tumor burden or MAPK activation despite confirmation of KRAS Q61R mutation. Several factors may explain this divergence. First, genetically engineered Kras/p53 models produce sustained, high-level oncogene activation, potentially rendering tumors more sensitive to endocrine modulation than carcinogen-induced adenomas arising under repeated mutational stress. Second, strain background and exposure protocol differ substantially; C57BL/6 mice require repeated urethane dosing and predominantly develop adenomas at the 20-week latency examined here, whereas estrogen-dependent effects may emerge more prominently in later-stage adenocarcinomas. In addition, because regions of interest for IHC staining and scoring were restricted to tumor epithelium and no differences were observed in tumor burden, cell proliferation, or ERK activation, these findings may reflect genotype-associated variation in mutant RAS protein abundance or tumor cell signaling state rather than differences in tumor growth, although the biological basis of this pattern requires further investigation. It is possible that by dissociating chromosomal and gonadal sex using the FCG system, our model may reveal that survival differences are mediated through systemic or inflammatory mechanisms rather than direct amplification of the KRAS–MAPK axis by estrogen signaling. Given the higher lymphocyte counts in XXF genotype airways compared to XYM, further investigating the tumor immune profile may potentially clarify the survival discrepancy between XXF and XYM genotypes in our model. The recent preprint report on genotype-dependent alterations in lymphocyte subsets in FCG mice suggests that unappreciated baseline immune differences might influence oncogenic processes, thus survival, in addition to effects attributable to urethane or gonadal hormones. This would support and justify further investigation of the behavior of tumor-infiltrating lymphocytes in the urethane-treated FCG model [35].

A major strength of the FCG model is its ability to dissociate sex chromosome complement from gonadal sex, thereby enabling causal testing of chromosomal versus hormonal contributions to disease phenotypes [11]. Unlike human cohort studies—where sex differences are confounded by variability in smoking history, occupational exposures, hormone replacement therapy, comorbidities, and genetic heterogeneity—the FCG system permits experimental control of chromosomal sex, endocrine status, and environmental exposure within a genetically homogeneous background (C57BL/6). In the context of urethane-induced lung tumorigenesis, this design allows uniform carcinogen dosing and timing, eliminating exposure variability that complicates epidemiologic inference. Because urethane reproducibly induces KRAS-driven lung adenomas in inbred strains with high penetrance, differences observed across genotypes can be interpreted mechanistically rather than correlationally. Thus, the FCG model overcomes key limitations of human observational studies—namely residual confounding, population stratification, and exposure heterogeneity—by enabling controlled, causal dissection of sex chromosome and hormonal influences on tumor initiation and progression.

The following limitations should be considered collectively when interpreting these findings: (1) constraints inherent to the FCG model, (2) the restricted malignant progression of urethane-induced tumors in C57BL/6 mice, (3) the use of a single carcinogen, (4) technical limitations of semi-quantitative IHC, (5) lymphoma-related survival confounding, and (6) the cross-sectional study design. A limitation inherent to the FCG model itself warrants transparent discussion. A recent study reported that the Y^Sry–^ chromosome used in a widely applied FCG line harbors a segment of duplicated genes from the X chromosome, resulting from a previously unrecognized X–Y translocation [36]. This translocation involves nine genes that could alter gene dose beyond the simple presence of XX versus XY complement and has prompted re-evaluation of phenotypes previously attributed solely to sex chromosome complement. Although the authors of that study note that many published FCG findings are unlikely to be substantially affected by this translocation, the presence of additional X-linked gene copies could nevertheless influence traits under investigation, particularly those with known X-chromosome gene involvement, including immune regulation and cancer susceptibility pathways. In the context of our lung carcinogenesis model, this translocation may affect processes such as tumor–immune crosstalk and non-cell-autonomous modifiers of tumor initiation in ways that cannot be fully dissected without additional experimental controls. Forward-looking studies will therefore benefit from explicit characterization of translocation status in FCG breeding colonies, evaluation of translocated gene expression in relevant tissues, and, where feasible, replication of key findings using alternative models or updated FCG lines with corrected chromosomal configurations.

Urethane exposure in C57BL/6 mice predominantly yields lung adenomas, and while these lesions model early KRAS-driven tumorigenesis, progression to invasive adenocarcinoma is limited, restricting conclusions regarding later-stage disease biology. In addition, the use of a single chemical carcinogen constrains generalizability to other etiologic contexts, such as tobacco smoke or genetically engineered models. Additionally, while animals were maintained under standardized husbandry conditions, individual biological variability, including differences in stress response, microbiome composition, or metabolic rate, cannot be fully excluded as a source of residual confounding. These factors were not directly measured in the current study, and future investigations incorporating controlled dietary interventions or physiological monitoring would help further isolate the contributions of sex chromosome complement and gonadal sex from environmental sources of variation. Immunohistochemical quantification relied on semi-quantitative H-score assessment, which, although widely used, does not provide absolute signaling intensity or cell-specific resolution. Because the anti-RAS (Q61R) antibody does not distinguish among RAS isoforms and is validated for human but not mouse tissue, staining is interpreted as relative RAS Q61R–associated immunoreactivity rather than absolute KRAS quantification. Given the predominance of Kras Q61 mutations in urethane-induced mouse lung tumors, the signal likely reflects mutant KRAS, although contributions from other RAS Q61R isoforms cannot be excluded [37].

Lymphoma-related morbidity is a potential confounder in long-term survival analyses of urethane-treated mice. Urethane is a multisite carcinogen capable of inducing lymphoid malignancies in addition to lung tumors [38]. In our study, some animals required euthanasia due to lymphoma rather than pulmonary tumor burden, limiting the interpretation of overall survival as a lung cancer–specific endpoint. Survival outcomes should therefore be considered alongside tumor multiplicity and histopathologic burden rather than in isolation. Finally, our analyses were performed at a single terminal time point, limiting the resolution of dynamic processes such as tumor initiation, clonal expansion, and immune evolution. Longitudinal studies incorporating earlier time points, lineage tracing, and molecular profiling will be essential to pinpoint the stages at which sex chromosome complement and gonadal sex exert their strongest effects. Future studies should also prioritize replication across additional carcinogen models and genetically engineered systems to assess the generalizability of the survival phenotype observed here. Immune profiling at the single-cell level, combined with transcriptomic characterization of tumor and stromal compartments stratified by FCG genotype, would help identify the specific systemic or microenvironmental pathways through which sex chromosome complement and gonadal sex modulate outcomes independently of tumor burden.

## 5. Conclusions

In summary, this study demonstrates that sex chromosome complement and gonadal sex each contribute to survival outcomes in urethane-induced lung carcinogenesis, operating through mechanisms that are independent of tumor burden, airway inflammation, MAPK signaling amplitude, and hepatic urethane bioactivation capacity. Across all four FCG genotypes, tumor multiplicity, tumor area, normalized tumor burden, and Ki-67 proliferation indices were comparable, as were BALF cellularity and ERK phosphorylation, indicating that the observed survival differences are not driven by differences in tumor growth or local immune response. The significant genotype effect on mutant RAS immunoreactivity, with the highest H-scores in XYF mice, points to a potential chromosomal influence on oncogenic signaling that warrants further mechanistic investigation. These findings highlight that sex shapes lung cancer biology through pathways beyond tumor-intrinsic proliferation, and reinforce the importance of incorporating sex as a biological variable in preclinical cancer models. The FCG system, by permitting independent manipulation of chromosomal and hormonal sex, provides a uniquely controlled framework for generating causal insights that can ultimately inform sex-informed approaches to lung cancer risk stratification, biomarker development, and therapeutic strategy.

## Figures and Tables

**Table 1:** Tumor incidence and histopathologic classification in urethane-treated FCG mice. Values represent the percentage of mice within each group that developed lung tumors, adenomas, or adenocarcinomas at the 30-week study endpoint. All urethane-treated mice developed lung tumors, whereas no tumors were observed in PBS-treated control animals. Percentages are reported relative to the total number of mice in each group (*n*). Results are presented for descriptive purposes.

Genotype	Treatment	Gonadal Sex	Tumor (%)	Adenoma (%)	Adenocarcinoma (%)	Number (*n*)
XXF	PBS	Female	0	0	0.0	9
	Urethane		100	100	12.5	8
XXM	PBS	Male	0	0	0	9
	Urethane		100	100	10.0	10
XYF	PBS	Female	0	0	0	8
	Urethane		100	100	33.3	9
XYM	PBS	Male	0	0	0	10
	Urethane		100	100	20	10

## Data Availability

Data available upon request.

## Supplementary Materials

The following supporting information can be downloaded at: https://www.mdpi.com/article/10.3390/cancers18071172/s1, Supplementary Data File S1: BCC Data Analysis Report; Supplementary Figure S1: Lymphoid Mass Histology; Supplementary Methods S1: KRAS (Q61R) PCR Workflow.

## Abbreviations

The following abbreviations are used in this manuscript:

AC	adenocarcinoma
AD	adenoma
BALF	bronchoalveolar lavage fluid
BIACUC	Bloomington Institutional Animal Care and Use Committee
BW	body weight
CI	confidence interval
Ct	cycle threshold
DAB	3,3′-diaminobenzidine
DNA	deoxyribonucleic acid
EDTA	ethylenediaminetetraacetic acid
EMM	estimated marginal mean
ERK	extracellular signal-regulated kinase
FCG	Four Core Genotypes
H&E	hematoxylin and eosin
HRP	horseradish peroxidase
IHC	immunohistochemistry
IL-6	interleukin 6
IP (i.p.)	intraperitoneal
JAX	The Jackson Laboratory
KRAS/Kras	Kirsten rat sarcoma viral oncogene homolog
LC	lung cancer
LR	likelihood ratio
MAPK	mitogen-activated protein kinase
mRNA	messenger ribonucleic acid
NaCl	sodium chloride
NCBI	National Center for Biotechnology Information
NF-κB	nuclear factor kappa B
NSCLC	non-small cell lung cancer
PBS	phosphate-buffered saline
PCR	polymerase chain reaction
p-ERK	phosphorylated extracellular signal-regulated kinase
PMN	polymorphonuclear leukocyte
qPCR	quantitative polymerase chain reaction
RAS	rat sarcoma
RANTES	regulated upon activation, normal T cell expressed and secreted
RNA	ribonucleic acid
ROI	region of interest
SEM	standard error of the mean
STAT3	signal transducer and activator of transcription 3
Tris	tris(hydroxymethyl)aminomethane
Tween-20	polysorbate 20
WT	wild-type
ΔCt	delta cycle threshold
2^−ΔΔCt^	comparative delta-delta cycle threshold method
