# Supplementary material for: Dissecting Sex Chromosome and Hormonal Contributions to Urethane-Induced Lung Tumorigenesis Using the Four Core Genotypes Mouse Model
Source: Cancers (Basel). 2026 Apr 5;18(7):1172. doi: 10.3390/cancers18071172 (PMC13072358; doi:10.3390/cancers18071172)
Supplement: Supplementary file 1 [file cancers-18-01172-s001.zip › Supplementary Data File S1. BCC Data Analysis Report.html]

Analyzing Lung tumor data from FCG mice, second dataset


Code 

- Show All Code
- Hide All Code

# Analyzing Lung tumor data from FCG mice, second dataset

#### Erik Parker for the Biostatistics Consulting Center (BCC)

#### 02 February 2026

NOTE: all post-hoc, pairwise comparisons from estimated marginal
means are adjusted for multiple comparisons using Tukey’s method.

# Mouse bodyweights

## Descriptive plots

Mean and 95% confidence intervals of weight over time for each
genotype and treatment.

```
weight_data %>%
    ggplot(aes(x = factor(week), y = weight, color = genotype)) +
    stat_summary(fun = mean,
                 geom = "point",
                 size = 3,
                 position = position_dodge(width = 0.3)) +
    stat_summary(fun.data = mean_cl_normal,
                 geom = "errorbar",
                 width = 0.1,
                 position = position_dodge(width = 0.3)) +
    geom_point(alpha = 0.2, 
               position = position_dodge(width = 0.3)) +
    facet_grid(~ treatment) +
    theme_minimal() +
    labs(x = "Week", y = "Weight", color = "Genotype")
```

## Linear mixed model analysis

Below fitting a linear mixed model looking for changes in weight over
time by week, genotype, and treatment - with a three-way interaction
between the variables. The animal IDs are used as a random intercept to
account for correlations within and between subjects over time.

We are also adjusting the model by the baseline weight for each
animal, this allows us to account for the fact that different animals
have different starting weights and so their weight change trajectories
may not be even. This is an alternative approach to modeling weight
change % which allows for fairer comparisons between groups by directly
accounting for differences in starting weight. It also makes better use
of the full information in the data, because baseline weight is strongly
related to weight over time. In contrast, percent change can be
misleading when subjects begin at very different weights (i.e. one mouse
going from 10 to 20 and another going from 20 to 40 would be the same %
increase, but a very different magnitude increase) and can introduce
extra variability, which reduces the clarity and reliability of the
results

The type 3 anova for the model (which we use to test for the
significance of the variables and interactions) shows that the three-way
interaction is significant, meaning that the combinations of treatment
and genotype groups show different weight trajectories over time
(mirroring what we saw in the descriptive plot above). Additionally, we
also see that all other two-way interactions and the main effects are
significant as well.

Next we will use some plots and the estimated marginal means to look
for differences between groups over time.

```
model <- weight_data %>%
  group_by(id) %>%
  mutate(baseline_weight = weight[week == 1]) %>% # calculate baseline weight for each subject, to adjust analysis by this
  ungroup() %>%
  lmer(weight ~ factor(week) * genotype * treatment + baseline_weight + (1 | id), data = .)

print(anova(model))
```

```
## Type III Analysis of Variance Table with Satterthwaite's method
##                                 Sum Sq Mean Sq NumDF DenDF  F value    Pr(>F)
## factor(week)                     65002 2241.46    29  4582 669.9382 < 2.2e-16
## genotype                            39   12.84     3   157   3.8383  0.010955
## treatment                          304  304.30     1   157  90.9512 < 2.2e-16
## baseline_weight                    311  310.63     1   157  92.8431 < 2.2e-16
## factor(week):genotype             1740   20.00    87  4582   5.9763 < 2.2e-16
## factor(week):treatment            1898   65.46    29  4582  19.5653 < 2.2e-16
## genotype:treatment                  70   23.18     3   157   6.9296  0.000207
## factor(week):genotype:treatment    807    9.28    87  4582   2.7736 7.061e-16
##                                    
## factor(week)                    ***
## genotype                        *  
## treatment                       ***
## baseline_weight                 ***
## factor(week):genotype           ***
## factor(week):treatment          ***
## genotype:treatment              ***
## factor(week):genotype:treatment ***
## ---
## Signif. codes:  0 '***' 0.001 '**' 0.01 '*' 0.05 '.' 0.1 ' ' 1
```

### Estimated marginal means

#### Comparing genotypes within treatment

```
summary(emmeans(model, pairwise ~ genotype | treatment * week))
```

```
## $emmeans
## treatment = PBS, week =  1:
##  genotype emmean    SE  df asymp.LCL asymp.UCL
##  XXF        20.8 0.643 Inf      19.5      22.0
##  XXM        20.8 0.790 Inf      19.2      22.3
##  XYF        20.8 0.729 Inf      19.4      22.2
##  XYM        20.8 0.773 Inf      19.3      22.3
## 
## treatment = Urethane, week =  1:
##  genotype emmean    SE  df asymp.LCL asymp.UCL
##  XXF        20.8 0.632 Inf      19.5      22.0
##  XXM        20.8 0.752 Inf      19.3      22.3
##  XYF        20.8 0.792 Inf      19.2      22.3
##  XYM        20.8 0.836 Inf      19.2      22.4
## 
## treatment = PBS, week =  2:
##  genotype emmean    SE  df asymp.LCL asymp.UCL
##  XXF        21.6 0.643 Inf      20.3      22.8
##  XXM        22.1 0.790 Inf      20.6      23.7
##  XYF        21.3 0.729 Inf      19.8      22.7
##  XYM        21.9 0.773 Inf      20.4      23.5
## 
## treatment = Urethane, week =  2:
##  genotype emmean    SE  df asymp.LCL asymp.UCL
##  XXF        20.4 0.632 Inf      19.2      21.7
##  XXM        20.7 0.752 Inf      19.3      22.2
##  XYF        20.6 0.792 Inf      19.1      22.2
##  XYM        20.9 0.836 Inf      19.2      22.5
## 
## treatment = PBS, week =  3:
##  genotype emmean    SE  df asymp.LCL asymp.UCL
##  XXF        22.1 0.643 Inf      20.8      23.3
##  XXM        23.2 0.790 Inf      21.7      24.8
##  XYF        22.1 0.729 Inf      20.7      23.6
##  XYM        22.6 0.773 Inf      21.1      24.1
## 
## treatment = Urethane, week =  3:
##  genotype emmean    SE  df asymp.LCL asymp.UCL
##  XXF        20.9 0.632 Inf      19.7      22.1
##  XXM        21.1 0.752 Inf      19.6      22.6
##  XYF        21.0 0.792 Inf      19.5      22.6
##  XYM        21.4 0.836 Inf      19.7      23.0
## 
## treatment = PBS, week =  4:
##  genotype emmean    SE  df asymp.LCL asymp.UCL
##  XXF        22.8 0.643 Inf      21.6      24.1
##  XXM        24.1 0.790 Inf      22.6      25.7
##  XYF        22.7 0.729 Inf      21.2      24.1
##  XYM        23.3 0.773 Inf      21.8      24.8
## 
## treatment = Urethane, week =  4:
##  genotype emmean    SE  df asymp.LCL asymp.UCL
##  XXF        21.0 0.632 Inf      19.8      22.2
##  XXM        21.2 0.752 Inf      19.7      22.6
##  XYF        21.3 0.792 Inf      19.8      22.9
##  XYM        21.9 0.836 Inf      20.3      23.6
## 
## treatment = PBS, week =  5:
##  genotype emmean    SE  df asymp.LCL asymp.UCL
##  XXF        23.6 0.643 Inf      22.3      24.8
##  XXM        25.0 0.790 Inf      23.4      26.5
##  XYF        23.0 0.729 Inf      21.6      24.5
##  XYM        24.0 0.773 Inf      22.5      25.5
## 
## treatment = Urethane, week =  5:
##  genotype emmean    SE  df asymp.LCL asymp.UCL
##  XXF        21.3 0.632 Inf      20.0      22.5
##  XXM        21.4 0.752 Inf      19.9      22.8
##  XYF        21.7 0.792 Inf      20.1      23.3
##  XYM        22.3 0.836 Inf      20.6      23.9
## 
## treatment = PBS, week =  6:
##  genotype emmean    SE  df asymp.LCL asymp.UCL
##  XXF        24.0 0.643 Inf      22.7      25.2
##  XXM        26.0 0.790 Inf      24.5      27.6
##  XYF        23.7 0.729 Inf      22.3      25.2
##  XYM        24.6 0.773 Inf      23.1      26.2
## 
## treatment = Urethane, week =  6:
##  genotype emmean    SE  df asymp.LCL asymp.UCL
##  XXF        21.3 0.632 Inf      20.1      22.5
##  XXM        21.6 0.752 Inf      20.2      23.1
##  XYF        21.9 0.792 Inf      20.4      23.5
##  XYM        22.3 0.836 Inf      20.7      24.0
## 
## treatment = PBS, week =  7:
##  genotype emmean    SE  df asymp.LCL asymp.UCL
##  XXF        24.5 0.643 Inf      23.2      25.7
##  XXM        27.0 0.790 Inf      25.4      28.5
##  XYF        24.2 0.729 Inf      22.8      25.6
##  XYM        25.1 0.773 Inf      23.6      26.6
## 
## treatment = Urethane, week =  7:
##  genotype emmean    SE  df asymp.LCL asymp.UCL
##  XXF        21.6 0.632 Inf      20.3      22.8
##  XXM        21.7 0.752 Inf      20.3      23.2
##  XYF        22.3 0.792 Inf      20.8      23.9
##  XYM        22.4 0.836 Inf      20.7      24.0
## 
## treatment = PBS, week =  8:
##  genotype emmean    SE  df asymp.LCL asymp.UCL
##  XXF        25.1 0.643 Inf      23.8      26.4
##  XXM        27.8 0.790 Inf      26.2      29.3
##  XYF        24.4 0.729 Inf      23.0      25.8
##  XYM        25.4 0.773 Inf      23.9      26.9
## 
## treatment = Urethane, week =  8:
##  genotype emmean    SE  df asymp.LCL asymp.UCL
##  XXF        21.6 0.632 Inf      20.4      22.9
##  XXM        21.8 0.752 Inf      20.3      23.3
##  XYF        22.4 0.792 Inf      20.8      23.9
##  XYM        22.4 0.836 Inf      20.8      24.1
## 
## treatment = PBS, week =  9:
##  genotype emmean    SE  df asymp.LCL asymp.UCL
##  XXF        25.4 0.643 Inf      24.2      26.7
##  XXM        28.3 0.790 Inf      26.8      29.9
##  XYF        24.8 0.729 Inf      23.3      26.2
##  XYM        25.7 0.773 Inf      24.2      27.2
## 
## treatment = Urethane, week =  9:
##  genotype emmean    SE  df asymp.LCL asymp.UCL
##  XXF        21.7 0.632 Inf      20.5      23.0
##  XXM        21.9 0.752 Inf      20.5      23.4
##  XYF        22.6 0.792 Inf      21.1      24.2
##  XYM        22.9 0.836 Inf      21.2      24.5
## 
## treatment = PBS, week = 10:
##  genotype emmean    SE  df asymp.LCL asymp.UCL
##  XXF        25.8 0.643 Inf      24.6      27.1
##  XXM        28.8 0.790 Inf      27.2      30.3
##  XYF        25.2 0.729 Inf      23.8      26.6
##  XYM        26.1 0.773 Inf      24.6      27.6
## 
## treatment = Urethane, week = 10:
##  genotype emmean    SE  df asymp.LCL asymp.UCL
##  XXF        21.9 0.632 Inf      20.7      23.2
##  XXM        22.0 0.752 Inf      20.6      23.5
##  XYF        22.5 0.792 Inf      21.0      24.1
##  XYM        22.7 0.836 Inf      21.1      24.3
## 
## treatment = PBS, week = 11:
##  genotype emmean    SE  df asymp.LCL asymp.UCL
##  XXF        26.9 0.643 Inf      25.6      28.2
##  XXM        29.7 0.790 Inf      28.2      31.3
##  XYF        25.6 0.729 Inf      24.2      27.0
##  XYM        26.6 0.773 Inf      25.1      28.1
## 
## treatment = Urethane, week = 11:
##  genotype emmean    SE  df asymp.LCL asymp.UCL
##  XXF        22.7 0.632 Inf      21.4      23.9
##  XXM        22.7 0.752 Inf      21.3      24.2
##  XYF        23.1 0.792 Inf      21.6      24.7
##  XYM        23.2 0.836 Inf      21.5      24.8
## 
## treatment = PBS, week = 12:
##  genotype emmean    SE  df asymp.LCL asymp.UCL
##  XXF        27.4 0.643 Inf      26.1      28.7
##  XXM        30.7 0.790 Inf      29.1      32.2
##  XYF        26.1 0.729 Inf      24.7      27.5
##  XYM        27.2 0.773 Inf      25.6      28.7
## 
## treatment = Urethane, week = 12:
##  genotype emmean    SE  df asymp.LCL asymp.UCL
##  XXF        23.6 0.632 Inf      22.4      24.9
##  XXM        23.5 0.752 Inf      22.0      24.9
##  XYF        23.6 0.792 Inf      22.1      25.2
##  XYM        24.0 0.836 Inf      22.4      25.6
## 
## treatment = PBS, week = 13:
##  genotype emmean    SE  df asymp.LCL asymp.UCL
##  XXF        28.1 0.643 Inf      26.8      29.3
##  XXM        31.5 0.790 Inf      30.0      33.1
##  XYF        26.5 0.729 Inf      25.1      28.0
##  XYM        27.6 0.773 Inf      26.1      29.1
## 
## treatment = Urethane, week = 13:
##  genotype emmean    SE  df asymp.LCL asymp.UCL
##  XXF        23.9 0.632 Inf      22.7      25.2
##  XXM        24.1 0.752 Inf      22.6      25.5
##  XYF        24.1 0.792 Inf      22.5      25.6
##  XYM        24.3 0.836 Inf      22.7      26.0
## 
## treatment = PBS, week = 14:
##  genotype emmean    SE  df asymp.LCL asymp.UCL
##  XXF        28.6 0.643 Inf      27.3      29.8
##  XXM        32.1 0.790 Inf      30.6      33.7
##  XYF        26.8 0.729 Inf      25.4      28.2
##  XYM        28.1 0.773 Inf      26.6      29.6
## 
## treatment = Urethane, week = 14:
##  genotype emmean    SE  df asymp.LCL asymp.UCL
##  XXF        23.9 0.632 Inf      22.7      25.1
##  XXM        24.4 0.752 Inf      23.0      25.9
##  XYF        24.5 0.792 Inf      22.9      26.0
##  XYM        24.8 0.836 Inf      23.2      26.5
## 
## treatment = PBS, week = 15:
##  genotype emmean    SE  df asymp.LCL asymp.UCL
##  XXF        29.1 0.643 Inf      27.8      30.3
##  XXM        32.8 0.790 Inf      31.2      34.3
##  XYF        27.5 0.729 Inf      26.1      29.0
##  XYM        28.7 0.773 Inf      27.2      30.2
## 
## treatment = Urethane, week = 15:
##  genotype emmean    SE  df asymp.LCL asymp.UCL
##  XXF        24.4 0.632 Inf      23.2      25.7
##  XXM        24.8 0.752 Inf      23.3      26.3
##  XYF        24.9 0.792 Inf      23.4      26.5
##  XYM        25.4 0.836 Inf      23.7      27.0
## 
## treatment = PBS, week = 16:
##  genotype emmean    SE  df asymp.LCL asymp.UCL
##  XXF        29.8 0.643 Inf      28.6      31.1
##  XXM        33.5 0.790 Inf      31.9      35.0
##  XYF        28.0 0.729 Inf      26.5      29.4
##  XYM        28.7 0.773 Inf      27.2      30.2
## 
## treatment = Urethane, week = 16:
##  genotype emmean    SE  df asymp.LCL asymp.UCL
##  XXF        24.7 0.632 Inf      23.4      25.9
##  XXM        25.0 0.752 Inf      23.5      26.5
##  XYF        25.4 0.792 Inf      23.9      27.0
##  XYM        25.8 0.836 Inf      24.2      27.5
## 
## treatment = PBS, week = 17:
##  genotype emmean    SE  df asymp.LCL asymp.UCL
##  XXF        30.0 0.643 Inf      28.8      31.3
##  XXM        33.9 0.790 Inf      32.3      35.4
##  XYF        28.5 0.729 Inf      27.1      30.0
##  XYM        28.9 0.773 Inf      27.4      30.4
## 
## treatment = Urethane, week = 17:
##  genotype emmean    SE  df asymp.LCL asymp.UCL
##  XXF        25.4 0.632 Inf      24.2      26.7
##  XXM        25.7 0.752 Inf      24.3      27.2
##  XYF        26.1 0.792 Inf      24.5      27.6
##  XYM        26.2 0.836 Inf      24.5      27.8
## 
## treatment = PBS, week = 18:
##  genotype emmean    SE  df asymp.LCL asymp.UCL
##  XXF        30.4 0.643 Inf      29.2      31.7
##  XXM        34.5 0.790 Inf      32.9      36.0
##  XYF        29.0 0.729 Inf      27.6      30.5
##  XYM        29.3 0.773 Inf      27.8      30.8
## 
## treatment = Urethane, week = 18:
##  genotype emmean    SE  df asymp.LCL asymp.UCL
##  XXF        25.8 0.632 Inf      24.5      27.0
##  XXM        26.0 0.752 Inf      24.5      27.4
##  XYF        26.5 0.792 Inf      24.9      28.0
##  XYM        26.4 0.836 Inf      24.8      28.1
## 
## treatment = PBS, week = 19:
##  genotype emmean    SE  df asymp.LCL asymp.UCL
##  XXF        31.0 0.643 Inf      29.8      32.3
##  XXM        35.2 0.790 Inf      33.7      36.8
##  XYF        29.3 0.729 Inf      27.8      30.7
##  XYM        29.4 0.773 Inf      27.9      30.9
## 
## treatment = Urethane, week = 19:
##  genotype emmean    SE  df asymp.LCL asymp.UCL
##  XXF        26.1 0.632 Inf      24.9      27.4
##  XXM        26.4 0.752 Inf      25.0      27.9
##  XYF        26.7 0.792 Inf      25.1      28.3
##  XYM        27.1 0.836 Inf      25.4      28.7
## 
## treatment = PBS, week = 20:
##  genotype emmean    SE  df asymp.LCL asymp.UCL
##  XXF        31.5 0.643 Inf      30.2      32.7
##  XXM        35.9 0.790 Inf      34.4      37.5
##  XYF        29.6 0.729 Inf      28.2      31.0
##  XYM        29.8 0.773 Inf      28.3      31.3
## 
## treatment = Urethane, week = 20:
##  genotype emmean    SE  df asymp.LCL asymp.UCL
##  XXF        26.6 0.632 Inf      25.4      27.8
##  XXM        26.9 0.752 Inf      25.4      28.3
##  XYF        27.0 0.792 Inf      25.4      28.5
##  XYM        27.2 0.836 Inf      25.6      28.9
## 
## treatment = PBS, week = 21:
##  genotype emmean    SE  df asymp.LCL asymp.UCL
##  XXF        31.6 0.643 Inf      30.4      32.9
##  XXM        36.3 0.790 Inf      34.8      37.9
##  XYF        30.0 0.729 Inf      28.6      31.5
##  XYM        30.3 0.773 Inf      28.8      31.8
## 
## treatment = Urethane, week = 21:
##  genotype emmean    SE  df asymp.LCL asymp.UCL
##  XXF        27.1 0.632 Inf      25.9      28.3
##  XXM        27.2 0.752 Inf      25.8      28.7
##  XYF        27.1 0.792 Inf      25.5      28.6
##  XYM        27.9 0.836 Inf      26.3      29.5
## 
## treatment = PBS, week = 22:
##  genotype emmean    SE  df asymp.LCL asymp.UCL
##  XXF        32.2 0.643 Inf      30.9      33.5
##  XXM        36.7 0.790 Inf      35.2      38.3
##  XYF        30.3 0.729 Inf      28.9      31.7
##  XYM        30.8 0.773 Inf      29.3      32.3
## 
## treatment = Urethane, week = 22:
##  genotype emmean    SE  df asymp.LCL asymp.UCL
##  XXF        28.0 0.632 Inf      26.7      29.2
##  XXM        27.7 0.752 Inf      26.2      29.2
##  XYF        27.5 0.792 Inf      25.9      29.0
##  XYM        28.2 0.836 Inf      26.6      29.8
## 
## treatment = PBS, week = 23:
##  genotype emmean    SE  df asymp.LCL asymp.UCL
##  XXF        32.6 0.643 Inf      31.3      33.8
##  XXM        37.0 0.790 Inf      35.5      38.6
##  XYF        30.6 0.729 Inf      29.2      32.0
##  XYM        30.9 0.773 Inf      29.4      32.4
## 
## treatment = Urethane, week = 23:
##  genotype emmean    SE  df asymp.LCL asymp.UCL
##  XXF        28.2 0.632 Inf      26.9      29.4
##  XXM        28.1 0.752 Inf      26.7      29.6
##  XYF        27.7 0.792 Inf      26.2      29.3
##  XYM        28.1 0.836 Inf      26.5      29.7
## 
## treatment = PBS, week = 24:
##  genotype emmean    SE  df asymp.LCL asymp.UCL
##  XXF        33.3 0.643 Inf      32.1      34.6
##  XXM        37.3 0.790 Inf      35.7      38.8
##  XYF        30.8 0.729 Inf      29.4      32.3
##  XYM        31.3 0.773 Inf      29.8      32.8
## 
## treatment = Urethane, week = 24:
##  genotype emmean    SE  df asymp.LCL asymp.UCL
##  XXF        29.0 0.632 Inf      27.8      30.3
##  XXM        28.5 0.752 Inf      27.0      30.0
##  XYF        28.1 0.792 Inf      26.6      29.7
##  XYM        28.6 0.836 Inf      27.0      30.2
## 
## treatment = PBS, week = 25:
##  genotype emmean    SE  df asymp.LCL asymp.UCL
##  XXF        33.8 0.643 Inf      32.6      35.1
##  XXM        37.7 0.790 Inf      36.1      39.2
##  XYF        31.2 0.729 Inf      29.7      32.6
##  XYM        31.6 0.773 Inf      30.1      33.1
## 
## treatment = Urethane, week = 25:
##  genotype emmean    SE  df asymp.LCL asymp.UCL
##  XXF        29.8 0.632 Inf      28.6      31.1
##  XXM        28.9 0.752 Inf      27.4      30.4
##  XYF        28.4 0.792 Inf      26.8      29.9
##  XYM        29.2 0.836 Inf      27.6      30.9
## 
## treatment = PBS, week = 26:
##  genotype emmean    SE  df asymp.LCL asymp.UCL
##  XXF        34.2 0.643 Inf      32.9      35.5
##  XXM        38.1 0.790 Inf      36.5      39.6
##  XYF        31.3 0.729 Inf      29.8      32.7
##  XYM        31.8 0.773 Inf      30.3      33.3
## 
## treatment = Urethane, week = 26:
##  genotype emmean    SE  df asymp.LCL asymp.UCL
##  XXF        30.2 0.632 Inf      29.0      31.4
##  XXM        29.1 0.752 Inf      27.6      30.5
##  XYF        28.7 0.792 Inf      27.2      30.3
##  XYM        29.0 0.836 Inf      27.3      30.6
## 
## treatment = PBS, week = 27:
##  genotype emmean    SE  df asymp.LCL asymp.UCL
##  XXF        35.0 0.643 Inf      33.8      36.3
##  XXM        38.2 0.790 Inf      36.7      39.8
##  XYF        31.6 0.729 Inf      30.2      33.1
##  XYM        31.7 0.773 Inf      30.2      33.2
## 
## treatment = Urethane, week = 27:
##  genotype emmean    SE  df asymp.LCL asymp.UCL
##  XXF        31.1 0.632 Inf      29.9      32.4
##  XXM        29.4 0.752 Inf      28.0      30.9
##  XYF        28.8 0.792 Inf      27.3      30.4
##  XYM        28.9 0.836 Inf      27.2      30.5
## 
## treatment = PBS, week = 28:
##  genotype emmean    SE  df asymp.LCL asymp.UCL
##  XXF        35.3 0.643 Inf      34.1      36.6
##  XXM        38.7 0.790 Inf      37.2      40.3
##  XYF        32.1 0.729 Inf      30.7      33.6
##  XYM        32.0 0.773 Inf      30.5      33.6
## 
## treatment = Urethane, week = 28:
##  genotype emmean    SE  df asymp.LCL asymp.UCL
##  XXF        31.6 0.632 Inf      30.4      32.9
##  XXM        29.9 0.752 Inf      28.5      31.4
##  XYF        29.3 0.792 Inf      27.7      30.8
##  XYM        29.5 0.836 Inf      27.8      31.1
## 
## treatment = PBS, week = 29:
##  genotype emmean    SE  df asymp.LCL asymp.UCL
##  XXF        35.9 0.643 Inf      34.6      37.2
##  XXM        38.8 0.790 Inf      37.2      40.3
##  XYF        32.4 0.729 Inf      31.0      33.8
##  XYM        32.4 0.773 Inf      30.9      34.0
## 
## treatment = Urethane, week = 29:
##  genotype emmean    SE  df asymp.LCL asymp.UCL
##  XXF        32.4 0.632 Inf      31.2      33.6
##  XXM        30.3 0.752 Inf      28.8      31.8
##  XYF        29.3 0.792 Inf      27.7      30.8
##  XYM        30.0 0.836 Inf      28.4      31.6
## 
## treatment = PBS, week = 30:
##  genotype emmean    SE  df asymp.LCL asymp.UCL
##  XXF        35.9 0.643 Inf      34.6      37.2
##  XXM        38.6 0.790 Inf      37.0      40.1
##  XYF        32.1 0.729 Inf      30.7      33.6
##  XYM        32.7 0.773 Inf      31.2      34.2
## 
## treatment = Urethane, week = 30:
##  genotype emmean    SE  df asymp.LCL asymp.UCL
##  XXF        32.3 0.632 Inf      31.1      33.6
##  XXM        29.8 0.752 Inf      28.3      31.3
##  XYF        29.2 0.792 Inf      27.6      30.7
##  XYM        29.5 0.836 Inf      27.8      31.1
## 
## Degrees-of-freedom method: asymptotic 
## Confidence level used: 0.95 
## 
## $contrasts
## treatment = PBS, week =  1:
##  contrast   estimate    SE  df z.ratio p.value
##  XXF - XXM -0.008679 1.090 Inf  -0.008  1.0000
##  XXF - XYF  0.000935 0.918 Inf   0.001  1.0000
##  XXF - XYM -0.005115 1.030 Inf  -0.005  1.0000
##  XXM - XYF  0.009614 1.170 Inf   0.008  1.0000
##  XXM - XYM  0.003564 1.050 Inf   0.003  1.0000
##  XYF - XYM -0.006050 1.100 Inf  -0.006  1.0000
## 
## treatment = Urethane, week =  1:
##  contrast   estimate    SE  df z.ratio p.value
##  XXF - XXM -0.007480 1.040 Inf  -0.007  1.0000
##  XXF - XYF  0.001415 0.955 Inf   0.001  1.0000
##  XXF - XYM -0.006083 1.090 Inf  -0.006  1.0000
##  XXM - XYF  0.008895 1.170 Inf   0.008  1.0000
##  XXM - XYM  0.001397 1.060 Inf   0.001  1.0000
##  XYF - XYM -0.007498 1.210 Inf  -0.006  1.0000
## 
## treatment = PBS, week =  2:
##  contrast   estimate    SE  df z.ratio p.value
##  XXF - XXM -0.577671 1.090 Inf  -0.529  0.9522
##  XXF - XYF  0.306704 0.918 Inf   0.334  0.9871
##  XXF - XYM -0.366404 1.030 Inf  -0.354  0.9848
##  XXM - XYF  0.884376 1.170 Inf   0.757  0.8736
##  XXM - XYM  0.211267 1.050 Inf   0.202  0.9971
##  XYF - XYM -0.673109 1.100 Inf  -0.612  0.9283
## 
## treatment = Urethane, week =  2:
##  contrast   estimate    SE  df z.ratio p.value
##  XXF - XXM -0.327903 1.040 Inf  -0.314  0.9893
##  XXF - XYF -0.202104 0.955 Inf  -0.212  0.9966
##  XXF - XYM -0.455935 1.090 Inf  -0.419  0.9753
##  XXM - XYF  0.125800 1.170 Inf   0.107  0.9996
##  XXM - XYM -0.128031 1.060 Inf  -0.121  0.9994
##  XYF - XYM -0.253831 1.210 Inf  -0.210  0.9967
## 
## treatment = PBS, week =  3:
##  contrast   estimate    SE  df z.ratio p.value
##  XXF - XXM -1.132800 1.090 Inf  -1.037  0.7279
##  XXF - XYF -0.063662 0.918 Inf  -0.069  0.9999
##  XXF - XYM -0.541336 1.030 Inf  -0.523  0.9536
##  XXM - XYF  1.069138 1.170 Inf   0.915  0.7966
##  XXM - XYM  0.591463 1.050 Inf   0.565  0.9425
##  XYF - XYM -0.477674 1.100 Inf  -0.434  0.9726
## 
## treatment = Urethane, week =  3:
##  contrast   estimate    SE  df z.ratio p.value
##  XXF - XXM -0.215205 1.040 Inf  -0.206  0.9969
##  XXF - XYF -0.130437 0.955 Inf  -0.137  0.9991
##  XXF - XYM -0.449046 1.090 Inf  -0.413  0.9763
##  XXM - XYF  0.084768 1.170 Inf   0.072  0.9999
##  XXM - XYM -0.233841 1.060 Inf  -0.221  0.9962
##  XYF - XYM -0.318609 1.210 Inf  -0.264  0.9936
## 
## treatment = PBS, week =  4:
##  contrast   estimate    SE  df z.ratio p.value
##  XXF - XXM -1.292598 1.090 Inf  -1.183  0.6377
##  XXF - XYF  0.191778 0.918 Inf   0.209  0.9968
##  XXF - XYM -0.456653 1.030 Inf  -0.441  0.9713
##  XXM - XYF  1.484376 1.170 Inf   1.271  0.5815
##  XXM - XYM  0.835945 1.050 Inf   0.798  0.8552
##  XYF - XYM -0.648431 1.100 Inf  -0.590  0.9353
## 
## treatment = Urethane, week =  4:
##  contrast   estimate    SE  df z.ratio p.value
##  XXF - XXM -0.165787 1.040 Inf  -0.159  0.9986
##  XXF - XYF -0.340067 0.955 Inf  -0.356  0.9845
##  XXF - XYM -0.922009 1.090 Inf  -0.847  0.8319
##  XXM - XYF -0.174280 1.170 Inf  -0.148  0.9988
##  XXM - XYM -0.756222 1.060 Inf  -0.714  0.8917
##  XYF - XYM -0.581942 1.210 Inf  -0.482  0.9630
## 
## treatment = PBS, week =  5:
##  contrast   estimate    SE  df z.ratio p.value
##  XXF - XXM -1.384723 1.090 Inf  -1.267  0.5839
##  XXF - XYF  0.532510 0.918 Inf   0.580  0.9381
##  XXF - XYM -0.402671 1.030 Inf  -0.389  0.9800
##  XXM - XYF  1.917233 1.170 Inf   1.642  0.3552
##  XXM - XYM  0.982051 1.050 Inf   0.938  0.7845
##  XYF - XYM -0.935181 1.100 Inf  -0.850  0.8303
## 
## treatment = Urethane, week =  5:
##  contrast   estimate    SE  df z.ratio p.value
##  XXF - XXM -0.086739 1.040 Inf  -0.083  0.9998
##  XXF - XYF -0.428400 0.955 Inf  -0.449  0.9699
##  XXF - XYM -0.992009 1.090 Inf  -0.911  0.7988
##  XXM - XYF -0.341660 1.170 Inf  -0.291  0.9914
##  XXM - XYM -0.905270 1.060 Inf  -0.855  0.8282
##  XYF - XYM -0.563609 1.210 Inf  -0.467  0.9662
## 
## treatment = PBS, week =  6:
##  contrast   estimate    SE  df z.ratio p.value
##  XXF - XXM -2.040437 1.090 Inf  -1.867  0.2422
##  XXF - XYF  0.233939 0.918 Inf   0.255  0.9942
##  XXF - XYM -0.687965 1.030 Inf  -0.665  0.9103
##  XXM - XYF  2.274376 1.170 Inf   1.947  0.2083
##  XXM - XYM  1.352472 1.050 Inf   1.292  0.5683
##  XYF - XYM -0.921904 1.100 Inf  -0.838  0.8362
## 
## treatment = Urethane, week =  6:
##  contrast   estimate    SE  df z.ratio p.value
##  XXF - XXM -0.342771 1.040 Inf  -0.329  0.9878
##  XXF - XYF -0.643955 0.955 Inf  -0.675  0.9067
##  XXF - XYM -1.046898 1.090 Inf  -0.962  0.7712
##  XXM - XYF -0.301184 1.170 Inf  -0.257  0.9941
##  XXM - XYM -0.704127 1.060 Inf  -0.665  0.9104
##  XYF - XYM -0.402942 1.210 Inf  -0.334  0.9872
## 
## treatment = PBS, week =  7:
##  contrast   estimate    SE  df z.ratio p.value
##  XXF - XXM -2.502232 1.090 Inf  -2.290  0.1003
##  XXF - XYF  0.293572 0.918 Inf   0.320  0.9887
##  XXF - XYM -0.647015 1.030 Inf  -0.625  0.9240
##  XXM - XYF  2.795804 1.170 Inf   2.394  0.0782
##  XXM - XYM  1.855217 1.050 Inf   1.772  0.2871
##  XYF - XYM -0.940588 1.100 Inf  -0.855  0.8278
## 
## treatment = Urethane, week =  7:
##  contrast   estimate    SE  df z.ratio p.value
##  XXF - XXM -0.156951 1.040 Inf  -0.150  0.9988
##  XXF - XYF -0.734881 0.955 Inf  -0.770  0.8681
##  XXF - XYM -0.766602 1.090 Inf  -0.704  0.8954
##  XXM - XYF -0.577930 1.170 Inf  -0.492  0.9608
##  XXM - XYM -0.609650 1.060 Inf  -0.575  0.9394
##  XYF - XYM -0.031720 1.210 Inf  -0.026  1.0000
## 
## treatment = PBS, week =  8:
##  contrast   estimate    SE  df z.ratio p.value
##  XXF - XXM -2.698038 1.090 Inf  -2.469  0.0648
##  XXF - XYF  0.693481 0.918 Inf   0.756  0.8743
##  XXF - XYM -0.327219 1.030 Inf  -0.316  0.9891
##  XXM - XYF  3.391518 1.170 Inf   2.904  0.0193
##  XXM - XYM  2.370819 1.050 Inf   2.264  0.1066
##  XYF - XYM -1.020700 1.100 Inf  -0.928  0.7898
## 
## treatment = Urethane, week =  8:
##  contrast   estimate    SE  df z.ratio p.value
##  XXF - XXM -0.138856 1.040 Inf  -0.133  0.9992
##  XXF - XYF -0.720437 0.955 Inf  -0.755  0.8747
##  XXF - XYM -0.798602 1.090 Inf  -0.734  0.8836
##  XXM - XYF -0.581581 1.170 Inf  -0.495  0.9601
##  XXM - XYM -0.659746 1.060 Inf  -0.623  0.9248
##  XYF - XYM -0.078165 1.210 Inf  -0.065  0.9999
## 
## treatment = PBS, week =  9:
##  contrast   estimate    SE  df z.ratio p.value
##  XXF - XXM -2.909063 1.090 Inf  -2.662  0.0389
##  XXF - XYF  0.662455 0.918 Inf   0.722  0.8885
##  XXF - XYM -0.304911 1.030 Inf  -0.295  0.9911
##  XXM - XYF  3.571518 1.170 Inf   3.058  0.0120
##  XXM - XYM  2.604152 1.050 Inf   2.487  0.0620
##  XYF - XYM -0.967366 1.100 Inf  -0.880  0.8155
## 
## treatment = Urethane, week =  9:
##  contrast   estimate    SE  df z.ratio p.value
##  XXF - XXM -0.180761 1.040 Inf  -0.173  0.9982
##  XXF - XYF -0.875437 0.955 Inf  -0.917  0.7957
##  XXF - XYM -1.126602 1.090 Inf  -1.035  0.7288
##  XXM - XYF -0.694676 1.170 Inf  -0.592  0.9346
##  XXM - XYM -0.945841 1.060 Inf  -0.893  0.8086
##  XYF - XYM -0.251165 1.210 Inf  -0.208  0.9968
## 
## treatment = PBS, week = 10:
##  contrast   estimate    SE  df z.ratio p.value
##  XXF - XXM -2.944961 1.090 Inf  -2.695  0.0355
##  XXF - XYF  0.619415 0.918 Inf   0.675  0.9067
##  XXF - XYM -0.280612 1.030 Inf  -0.271  0.9930
##  XXM - XYF  3.564376 1.170 Inf   3.052  0.0122
##  XXM - XYM  2.664348 1.050 Inf   2.544  0.0534
##  XYF - XYM -0.900027 1.100 Inf  -0.818  0.8458
## 
## treatment = Urethane, week = 10:
##  contrast   estimate    SE  df z.ratio p.value
##  XXF - XXM -0.093300 1.040 Inf  -0.089  0.9997
##  XXF - XYF -0.593770 0.955 Inf  -0.622  0.9251
##  XXF - XYM -0.751046 1.090 Inf  -0.690  0.9010
##  XXM - XYF -0.500470 1.170 Inf  -0.426  0.9740
##  XXM - XYM -0.657746 1.060 Inf  -0.621  0.9254
##  XYF - XYM -0.157276 1.210 Inf  -0.130  0.9992
## 
## treatment = PBS, week = 11:
##  contrast   estimate    SE  df z.ratio p.value
##  XXF - XXM -2.841444 1.090 Inf  -2.600  0.0460
##  XXF - XYF  1.292455 0.918 Inf   1.408  0.4942
##  XXF - XYM  0.267442 1.030 Inf   0.258  0.9940
##  XXM - XYF  4.133899 1.170 Inf   3.540  0.0023
##  XXM - XYM  3.108886 1.050 Inf   2.969  0.0158
##  XYF - XYM -1.025013 1.100 Inf  -0.932  0.7876
## 
## treatment = Urethane, week = 11:
##  contrast   estimate    SE  df z.ratio p.value
##  XXF - XXM -0.053777 1.040 Inf  -0.052  1.0000
##  XXF - XYF -0.464326 0.955 Inf  -0.486  0.9621
##  XXF - XYM -0.489046 1.090 Inf  -0.449  0.9698
##  XXM - XYF -0.410549 1.170 Inf  -0.350  0.9853
##  XXM - XYM -0.435270 1.060 Inf  -0.411  0.9766
##  XYF - XYM -0.024720 1.210 Inf  -0.020  1.0000
## 
## treatment = PBS, week = 12:
##  contrast   estimate    SE  df z.ratio p.value
##  XXF - XXM -3.260016 1.090 Inf  -2.983  0.0151
##  XXF - XYF  1.274169 0.918 Inf   1.388  0.5067
##  XXF - XYM  0.235677 1.030 Inf   0.228  0.9958
##  XXM - XYF  4.534185 1.170 Inf   3.882  0.0006
##  XXM - XYM  3.495693 1.050 Inf   3.338  0.0047
##  XYF - XYM -1.038492 1.100 Inf  -0.944  0.7809
## 
## treatment = Urethane, week = 12:
##  contrast   estimate    SE  df z.ratio p.value
##  XXF - XXM  0.144054 1.040 Inf   0.138  0.9991
##  XXF - XYF -0.033955 0.955 Inf  -0.036  1.0000
##  XXF - XYM -0.388453 1.090 Inf  -0.357  0.9844
##  XXM - XYF -0.178010 1.170 Inf  -0.152  0.9988
##  XXM - XYM -0.532508 1.060 Inf  -0.503  0.9585
##  XYF - XYM -0.354498 1.210 Inf  -0.294  0.9912
## 
## treatment = PBS, week = 13:
##  contrast   estimate    SE  df z.ratio p.value
##  XXF - XXM -3.466609 1.090 Inf  -3.172  0.0082
##  XXF - XYF  1.518243 0.918 Inf   1.654  0.3483
##  XXF - XYM  0.418075 1.030 Inf   0.404  0.9777
##  XXM - XYF  4.984852 1.170 Inf   4.268  0.0001
##  XXM - XYM  3.884684 1.050 Inf   3.710  0.0012
##  XYF - XYM -1.100167 1.100 Inf  -1.000  0.7492
## 
## treatment = Urethane, week = 13:
##  contrast   estimate    SE  df z.ratio p.value
##  XXF - XXM -0.151660 1.040 Inf  -0.145  0.9989
##  XXF - XYF -0.150067 0.955 Inf  -0.157  0.9986
##  XXF - XYM -0.406453 1.090 Inf  -0.373  0.9822
##  XXM - XYF  0.001593 1.170 Inf   0.001  1.0000
##  XXM - XYM -0.254793 1.060 Inf  -0.241  0.9951
##  XYF - XYM -0.256387 1.210 Inf  -0.212  0.9966
## 
## treatment = PBS, week = 14:
##  contrast   estimate    SE  df z.ratio p.value
##  XXF - XXM -3.535757 1.090 Inf  -3.236  0.0067
##  XXF - XYF  1.766713 0.918 Inf   1.925  0.2175
##  XXF - XYM  0.481028 1.030 Inf   0.465  0.9667
##  XXM - XYF  5.302471 1.170 Inf   4.540  <.0001
##  XXM - XYM  4.016785 1.050 Inf   3.836  0.0007
##  XYF - XYM -1.285686 1.100 Inf  -1.169  0.6464
## 
## treatment = Urethane, week = 14:
##  contrast   estimate    SE  df z.ratio p.value
##  XXF - XXM -0.529650 1.040 Inf  -0.508  0.9573
##  XXF - XYF -0.569881 0.955 Inf  -0.597  0.9330
##  XXF - XYM -0.908824 1.090 Inf  -0.835  0.8378
##  XXM - XYF -0.040232 1.170 Inf  -0.034  1.0000
##  XXM - XYM -0.379174 1.060 Inf  -0.358  0.9843
##  XYF - XYM -0.338942 1.210 Inf  -0.281  0.9923
## 
## treatment = PBS, week = 15:
##  contrast   estimate    SE  df z.ratio p.value
##  XXF - XXM -3.702268 1.090 Inf  -3.388  0.0039
##  XXF - XYF  1.513536 0.918 Inf   1.649  0.3511
##  XXF - XYM  0.353256 1.030 Inf   0.341  0.9863
##  XXM - XYF  5.215804 1.170 Inf   4.466  <.0001
##  XXM - XYM  4.055525 1.050 Inf   3.873  0.0006
##  XYF - XYM -1.160279 1.100 Inf  -1.055  0.7169
## 
## treatment = Urethane, week = 15:
##  contrast   estimate    SE  df z.ratio p.value
##  XXF - XXM -0.371396 1.040 Inf  -0.356  0.9846
##  XXF - XYF -0.525437 0.955 Inf  -0.550  0.9465
##  XXF - XYM -0.952379 1.090 Inf  -0.875  0.8178
##  XXM - XYF -0.154041 1.170 Inf  -0.131  0.9992
##  XXM - XYM -0.580984 1.060 Inf  -0.548  0.9470
##  XYF - XYM -0.426942 1.210 Inf  -0.354  0.9848
## 
## treatment = PBS, week = 16:
##  contrast   estimate    SE  df z.ratio p.value
##  XXF - XXM -3.652305 1.090 Inf  -3.342  0.0046
##  XXF - XYF  1.855404 0.918 Inf   2.021  0.1800
##  XXF - XYM  1.089998 1.030 Inf   1.053  0.7180
##  XXM - XYF  5.507709 1.170 Inf   4.716  <.0001
##  XXM - XYM  4.742304 1.050 Inf   4.529  <.0001
##  XYF - XYM -0.765405 1.100 Inf  -0.696  0.8987
## 
## treatment = Urethane, week = 16:
##  contrast   estimate    SE  df z.ratio p.value
##  XXF - XXM -0.310549 1.040 Inf  -0.298  0.9908
##  XXF - XYF -0.745622 0.955 Inf  -0.781  0.8631
##  XXF - XYM -1.151342 1.090 Inf  -1.058  0.7152
##  XXM - XYF -0.435073 1.170 Inf  -0.371  0.9826
##  XXM - XYM -0.840793 1.060 Inf  -0.794  0.8574
##  XYF - XYM -0.405720 1.210 Inf  -0.336  0.9869
## 
## treatment = PBS, week = 17:
##  contrast   estimate    SE  df z.ratio p.value
##  XXF - XXM -3.835528 1.090 Inf  -3.510  0.0025
##  XXF - XYF  1.509800 0.918 Inf   1.645  0.3534
##  XXF - XYM  1.136831 1.030 Inf   1.098  0.6905
##  XXM - XYF  5.345328 1.170 Inf   4.577  <.0001
##  XXM - XYM  4.972360 1.050 Inf   4.748  <.0001
##  XYF - XYM -0.372968 1.100 Inf  -0.339  0.9866
## 
## treatment = Urethane, week = 17:
##  contrast   estimate    SE  df z.ratio p.value
##  XXF - XXM -0.319120 1.040 Inf  -0.306  0.9901
##  XXF - XYF -0.668955 0.955 Inf  -0.701  0.8968
##  XXF - XYM -0.744009 1.090 Inf  -0.684  0.9034
##  XXM - XYF -0.349835 1.170 Inf  -0.298  0.9908
##  XXM - XYM -0.424889 1.060 Inf  -0.401  0.9782
##  XYF - XYM -0.075053 1.210 Inf  -0.062  0.9999
## 
## treatment = PBS, week = 18:
##  contrast   estimate    SE  df z.ratio p.value
##  XXF - XXM -4.009430 1.090 Inf  -3.669  0.0014
##  XXF - XYF  1.413517 0.918 Inf   1.540  0.4135
##  XXF - XYM  1.162922 1.030 Inf   1.124  0.6749
##  XXM - XYF  5.422947 1.170 Inf   4.643  <.0001
##  XXM - XYM  5.172351 1.050 Inf   4.939  <.0001
##  XYF - XYM -0.250596 1.100 Inf  -0.228  0.9958
## 
## treatment = Urethane, week = 18:
##  contrast   estimate    SE  df z.ratio p.value
##  XXF - XXM -0.183618 1.040 Inf  -0.176  0.9981
##  XXF - XYF -0.703770 0.955 Inf  -0.737  0.8821
##  XXF - XYM -0.659268 1.090 Inf  -0.606  0.9303
##  XXM - XYF -0.520153 1.170 Inf  -0.443  0.9710
##  XXM - XYM -0.475650 1.060 Inf  -0.449  0.9698
##  XYF - XYM  0.044502 1.210 Inf   0.037  1.0000
## 
## treatment = PBS, week = 19:
##  contrast   estimate    SE  df z.ratio p.value
##  XXF - XXM -4.187360 1.090 Inf  -3.832  0.0007
##  XXF - XYF  1.770349 0.918 Inf   1.929  0.2159
##  XXF - XYM  1.597465 1.030 Inf   1.544  0.4114
##  XXM - XYF  5.957709 1.170 Inf   5.101  <.0001
##  XXM - XYM  5.784825 1.050 Inf   5.524  <.0001
##  XYF - XYM -0.172884 1.100 Inf  -0.157  0.9986
## 
## treatment = Urethane, week = 19:
##  contrast   estimate    SE  df z.ratio p.value
##  XXF - XXM -0.282919 1.040 Inf  -0.271  0.9930
##  XXF - XYF -0.556326 0.955 Inf  -0.583  0.9373
##  XXF - XYM -0.941046 1.090 Inf  -0.865  0.8231
##  XXM - XYF -0.273407 1.170 Inf  -0.233  0.9956
##  XXM - XYM -0.658127 1.060 Inf  -0.621  0.9253
##  XYF - XYM -0.384720 1.210 Inf  -0.319  0.9888
## 
## treatment = PBS, week = 20:
##  contrast   estimate    SE  df z.ratio p.value
##  XXF - XXM -4.450510 1.090 Inf  -4.073  0.0003
##  XXF - XYF  1.846723 0.918 Inf   2.012  0.1835
##  XXF - XYM  1.640813 1.030 Inf   1.585  0.3869
##  XXM - XYF  6.297233 1.170 Inf   5.392  <.0001
##  XXM - XYM  6.091323 1.050 Inf   5.817  <.0001
##  XYF - XYM -0.205910 1.100 Inf  -0.187  0.9977
## 
## treatment = Urethane, week = 20:
##  contrast   estimate    SE  df z.ratio p.value
##  XXF - XXM -0.266475 1.040 Inf  -0.255  0.9942
##  XXF - XYF -0.362659 0.955 Inf  -0.380  0.9813
##  XXF - XYM -0.633935 1.090 Inf  -0.582  0.9374
##  XXM - XYF -0.096184 1.170 Inf  -0.082  0.9998
##  XXM - XYM -0.367460 1.060 Inf  -0.347  0.9857
##  XYF - XYM -0.271276 1.210 Inf  -0.225  0.9960
## 
## treatment = PBS, week = 21:
##  contrast   estimate    SE  df z.ratio p.value
##  XXF - XXM -4.715730 1.090 Inf  -4.316  0.0001
##  XXF - XYF  1.590074 0.918 Inf   1.732  0.3068
##  XXF - XYM  1.294501 1.030 Inf   1.251  0.5944
##  XXM - XYF  6.305804 1.170 Inf   5.399  <.0001
##  XXM - XYM  6.010231 1.050 Inf   5.739  <.0001
##  XYF - XYM -0.295574 1.100 Inf  -0.269  0.9932
## 
## treatment = Urethane, week = 21:
##  contrast   estimate    SE  df z.ratio p.value
##  XXF - XXM -0.148380 1.040 Inf  -0.142  0.9990
##  XXF - XYF  0.036785 0.955 Inf   0.039  1.0000
##  XXF - XYM -0.799535 1.090 Inf  -0.735  0.8832
##  XXM - XYF  0.185165 1.170 Inf   0.158  0.9986
##  XXM - XYM -0.651155 1.060 Inf  -0.615  0.9275
##  XYF - XYM -0.836320 1.210 Inf  -0.693  0.8998
## 
## treatment = PBS, week = 22:
##  contrast   estimate    SE  df z.ratio p.value
##  XXF - XXM -4.533917 1.090 Inf  -4.149  0.0002
##  XXF - XYF  1.875221 0.918 Inf   2.043  0.1723
##  XXF - XYM  1.394297 1.030 Inf   1.347  0.5327
##  XXM - XYF  6.409138 1.170 Inf   5.488  <.0001
##  XXM - XYM  5.928214 1.050 Inf   5.661  <.0001
##  XYF - XYM -0.480924 1.100 Inf  -0.437  0.9720
## 
## treatment = Urethane, week = 22:
##  contrast   estimate    SE  df z.ratio p.value
##  XXF - XXM  0.277758 1.040 Inf   0.266  0.9934
##  XXF - XYF  0.480304 0.955 Inf   0.503  0.9584
##  XXF - XYM -0.222750 1.090 Inf  -0.205  0.9970
##  XXM - XYF  0.202546 1.170 Inf   0.173  0.9982
##  XXM - XYM -0.500508 1.060 Inf  -0.472  0.9651
##  XYF - XYM -0.703053 1.210 Inf  -0.583  0.9373
## 
## treatment = PBS, week = 23:
##  contrast   estimate    SE  df z.ratio p.value
##  XXF - XXM -4.490364 1.090 Inf  -4.109  0.0002
##  XXF - XYF  1.974488 0.918 Inf   2.151  0.1372
##  XXF - XYM  1.647374 1.030 Inf   1.592  0.3833
##  XXM - XYF  6.464852 1.170 Inf   5.535  <.0001
##  XXM - XYM  6.137738 1.050 Inf   5.861  <.0001
##  XYF - XYM -0.327114 1.100 Inf  -0.297  0.9909
## 
## treatment = Urethane, week = 23:
##  contrast   estimate    SE  df z.ratio p.value
##  XXF - XXM  0.019239 1.040 Inf   0.018  1.0000
##  XXF - XYF  0.426785 0.955 Inf   0.447  0.9702
##  XXF - XYM  0.053398 1.090 Inf   0.049  1.0000
##  XXM - XYF  0.407546 1.170 Inf   0.347  0.9856
##  XXM - XYM  0.034159 1.060 Inf   0.032  1.0000
##  XYF - XYM -0.373387 1.210 Inf  -0.309  0.9897
## 
## treatment = PBS, week = 24:
##  contrast   estimate    SE  df z.ratio p.value
##  XXF - XXM -3.949448 1.090 Inf  -3.614  0.0017
##  XXF - XYF  2.483499 0.918 Inf   2.706  0.0344
##  XXF - XYM  1.987057 1.030 Inf   1.920  0.2195
##  XXM - XYF  6.432947 1.170 Inf   5.508  <.0001
##  XXM - XYM  5.936505 1.050 Inf   5.669  <.0001
##  XYF - XYM -0.496442 1.100 Inf  -0.451  0.9694
## 
## treatment = Urethane, week = 24:
##  contrast   estimate    SE  df z.ratio p.value
##  XXF - XXM  0.561779 1.040 Inf   0.538  0.9497
##  XXF - XYF  0.931396 0.955 Inf   0.976  0.7633
##  XXF - XYM  0.449176 1.090 Inf   0.413  0.9763
##  XXM - XYF  0.369617 1.170 Inf   0.315  0.9892
##  XXM - XYM -0.112603 1.060 Inf  -0.106  0.9996
##  XYF - XYM -0.482220 1.210 Inf  -0.400  0.9784
## 
## treatment = PBS, week = 25:
##  contrast   estimate    SE  df z.ratio p.value
##  XXF - XXM -3.855602 1.090 Inf  -3.528  0.0024
##  XXF - XYF  2.687821 0.918 Inf   2.928  0.0179
##  XXF - XYM  2.240903 1.030 Inf   2.165  0.1330
##  XXM - XYF  6.543423 1.170 Inf   5.603  <.0001
##  XXM - XYM  6.096505 1.050 Inf   5.822  <.0001
##  XYF - XYM -0.446918 1.100 Inf  -0.406  0.9773
## 
## treatment = Urethane, week = 25:
##  contrast   estimate    SE  df z.ratio p.value
##  XXF - XXM  0.935430 1.040 Inf   0.896  0.8067
##  XXF - XYF  1.459563 0.955 Inf   1.529  0.4200
##  XXF - XYM  0.569398 1.090 Inf   0.523  0.9535
##  XXM - XYF  0.524133 1.170 Inf   0.446  0.9703
##  XXM - XYM -0.366031 1.060 Inf  -0.346  0.9858
##  XYF - XYM -0.890165 1.210 Inf  -0.738  0.8819
## 
## treatment = PBS, week = 26:
##  contrast   estimate    SE  df z.ratio p.value
##  XXF - XXM -3.873642 1.090 Inf  -3.545  0.0022
##  XXF - XYF  2.933591 0.918 Inf   3.196  0.0076
##  XXF - XYM  2.399614 1.030 Inf   2.319  0.0938
##  XXM - XYF  6.807233 1.170 Inf   5.828  <.0001
##  XXM - XYM  6.273256 1.050 Inf   5.990  <.0001
##  XYF - XYM -0.533977 1.100 Inf  -0.486  0.9623
## 
## treatment = Urethane, week = 26:
##  contrast   estimate    SE  df z.ratio p.value
##  XXF - XXM  1.119927 1.040 Inf   1.073  0.7058
##  XXF - XYF  1.455489 0.955 Inf   1.525  0.4226
##  XXF - XYM  1.215324 1.090 Inf   1.117  0.6793
##  XXM - XYF  0.335562 1.170 Inf   0.286  0.9919
##  XXM - XYM  0.095397 1.060 Inf   0.090  0.9997
##  XYF - XYM -0.240165 1.210 Inf  -0.199  0.9972
## 
## treatment = PBS, week = 27:
##  contrast   estimate    SE  df z.ratio p.value
##  XXF - XXM -3.212195 1.090 Inf  -2.940  0.0173
##  XXF - XYF  3.387228 0.918 Inf   3.690  0.0013
##  XXF - XYM  3.368596 1.030 Inf   3.255  0.0062
##  XXM - XYF  6.599423 1.170 Inf   5.651  <.0001
##  XXM - XYM  6.580791 1.050 Inf   6.284  <.0001
##  XYF - XYM -0.018632 1.100 Inf  -0.017  1.0000
## 
## treatment = Urethane, week = 27:
##  contrast   estimate    SE  df z.ratio p.value
##  XXF - XXM  1.688710 1.040 Inf   1.618  0.3681
##  XXF - XYF  2.295304 0.955 Inf   2.405  0.0762
##  XXF - XYM  2.257250 1.090 Inf   2.074  0.1617
##  XXM - XYF  0.606593 1.170 Inf   0.517  0.9551
##  XXM - XYM  0.568540 1.060 Inf   0.537  0.9501
##  XYF - XYM -0.038053 1.210 Inf  -0.032  1.0000
## 
## treatment = PBS, week = 28:
##  contrast   estimate    SE  df z.ratio p.value
##  XXF - XXM -3.373276 1.090 Inf  -3.087  0.0109
##  XXF - XYF  3.220624 0.918 Inf   3.509  0.0025
##  XXF - XYM  3.295722 1.030 Inf   3.185  0.0079
##  XXM - XYF  6.593899 1.170 Inf   5.646  <.0001
##  XXM - XYM  6.668998 1.050 Inf   6.368  <.0001
##  XYF - XYM  0.075099 1.100 Inf   0.068  0.9999
## 
## treatment = Urethane, week = 28:
##  contrast   estimate    SE  df z.ratio p.value
##  XXF - XXM  1.706012 1.040 Inf   1.635  0.3588
##  XXF - XYF  2.355304 0.955 Inf   2.467  0.0651
##  XXF - XYM  2.166361 1.090 Inf   1.990  0.1916
##  XXM - XYF  0.649292 1.170 Inf   0.553  0.9458
##  XXM - XYM  0.460350 1.060 Inf   0.435  0.9725
##  XYF - XYM -0.188942 1.210 Inf  -0.157  0.9986
## 
## treatment = PBS, week = 29:
##  contrast   estimate    SE  df z.ratio p.value
##  XXF - XXM -2.853770 1.090 Inf  -2.612  0.0446
##  XXF - XYF  3.489177 0.918 Inf   3.801  0.0008
##  XXF - XYM  3.465564 1.030 Inf   3.349  0.0045
##  XXM - XYF  6.342947 1.170 Inf   5.431  <.0001
##  XXM - XYM  6.319334 1.050 Inf   6.034  <.0001
##  XYF - XYM -0.023613 1.100 Inf  -0.021  1.0000
## 
## treatment = Urethane, week = 29:
##  contrast   estimate    SE  df z.ratio p.value
##  XXF - XXM  2.109345 1.040 Inf   2.022  0.1800
##  XXF - XYF  3.156970 0.955 Inf   3.307  0.0052
##  XXF - XYM  2.415028 1.090 Inf   2.219  0.1181
##  XXM - XYF  1.047625 1.170 Inf   0.892  0.8089
##  XXM - XYM  0.305683 1.060 Inf   0.289  0.9916
##  XYF - XYM -0.741942 1.210 Inf  -0.615  0.9274
## 
## treatment = PBS, week = 30:
##  contrast   estimate    SE  df z.ratio p.value
##  XXF - XXM -2.655510 1.090 Inf  -2.430  0.0715
##  XXF - XYF  3.780770 0.918 Inf   4.119  0.0002
##  XXF - XYM  3.191107 1.030 Inf   3.083  0.0110
##  XXM - XYF  6.436280 1.170 Inf   5.511  <.0001
##  XXM - XYM  5.846617 1.050 Inf   5.583  <.0001
##  XYF - XYM -0.589663 1.100 Inf  -0.536  0.9502
## 
## treatment = Urethane, week = 30:
##  contrast   estimate    SE  df z.ratio p.value
##  XXF - XXM  2.513843 1.040 Inf   2.409  0.0753
##  XXF - XYF  3.122896 0.955 Inf   3.271  0.0059
##  XXF - XYM  2.834287 1.090 Inf   2.604  0.0455
##  XXM - XYF  0.609054 1.170 Inf   0.519  0.9546
##  XXM - XYM  0.320445 1.060 Inf   0.302  0.9904
##  XYF - XYM -0.288609 1.210 Inf  -0.239  0.9952
## 
## Degrees-of-freedom method: asymptotic 
## P value adjustment: tukey method for comparing a family of 4 estimates
```

Below is a plot of the above table of estimated weight values.

Because there are too many significant differences at each week,
instead I am just marking a week with a star (\*) if there is at least
one pairwise significant difference that week. As you can see, most
weeks have at least one significant difference in the PBS group, but
there are fewer in the Urethane group (just at weeks 29 and 30).

```
# Compute emmeans with pairwise comparisons
emm <- emmeans(model, pairwise ~ genotype | treatment * week)

# Extract the estimated marginal means (EMMs)
emm_df <- as.data.frame(emm$emmeans) 

# Extract the pairwise comparisons
pwc <- as.data.frame(emm$contrasts)

# Add y-position for p-value annotations
pwc <- pwc %>%
    group_by(treatment, week) %>%
    mutate(y.position = max(emm_df$asymp.UCL[emm_df$treatment == treatment &
                                              emm_df$week == week]) + 
               seq(0.3, 0.3, length.out = n())) %>%
    ungroup() %>%
    mutate(p.value = ifelse(p.value > 0.05, "", format.pval(p.value, eps = 0.001, digits = 1)))

# Create the plot
p <- ggplot(emm_df, aes(x = week, y = emmean, color = genotype)) +
    geom_point(position = position_dodge(width = 0.1), size = 3) +
    geom_errorbar(aes(ymin = asymp.LCL, ymax = asymp.UCL), 
                  width = 0.2, position = position_dodge(width = 0.1)) +
    geom_line(aes(group = genotype)) +  # make a trend line across the weeks
    facet_wrap(~ treatment) + # facet the plots by one grouping variable
    theme_bw() + # use a simple BW theme
    labs(title = paste("Effect plot of expected weight, by genotype, treatment across time"), y = paste("Predicted value of weight"), x = "Week", color = "Genotype")

# format data for stat_pvalue
pwc <- pwc %>%
    mutate(
        treat1 = sub(" - .*", "", contrast),
        treat2 = sub(".* - ", "", contrast),
        y.position = max(emm_df$asymp.UCL) + seq(0.3, 0.3, length.out = n()),
        group1 = paste(treatment, treat1, sep = "-"),
        group2 = paste(treatment, treat2, sep = "-")
    ) %>%
  group_by(treatment, week) %>%
  mutate(p_val_star = ifelse(p.value != "", "*", "")) %>% # too many significant weeks, just adding a star instead if there are any pairwise significant groups (of the four)
  ungroup()
    # mutate(p.value = ifelse(p.value != "",
    #                         paste(group1, "vs.", group2, " p = ", p.value),
    #                         ""))


# Add p-values and display the plot
final_plot <- p + stat_pvalue_manual(pwc, 
                       x = "week",
                       label = "p_val_star", 
                       y.position = "y.position",
                       tip.length = 0.01,
                       step.increase = 0.5,
                       label.size = 4)


print(final_plot)
```

#### Comparing treatments within genotypes

```
emmeans(model, pairwise ~ treatment | genotype * week)
```

```
## $emmeans
## genotype = XXF, week =  1:
##  treatment emmean    SE  df asymp.LCL asymp.UCL
##  PBS         20.8 0.643 Inf      19.5      22.0
##  Urethane    20.8 0.632 Inf      19.5      22.0
## 
## genotype = XXM, week =  1:
##  treatment emmean    SE  df asymp.LCL asymp.UCL
##  PBS         20.8 0.790 Inf      19.2      22.3
##  Urethane    20.8 0.752 Inf      19.3      22.3
## 
## genotype = XYF, week =  1:
##  treatment emmean    SE  df asymp.LCL asymp.UCL
##  PBS         20.8 0.729 Inf      19.4      22.2
##  Urethane    20.8 0.792 Inf      19.2      22.3
## 
## genotype = XYM, week =  1:
##  treatment emmean    SE  df asymp.LCL asymp.UCL
##  PBS         20.8 0.773 Inf      19.3      22.3
##  Urethane    20.8 0.836 Inf      19.2      22.4
## 
## genotype = XXF, week =  2:
##  treatment emmean    SE  df asymp.LCL asymp.UCL
##  PBS         21.6 0.643 Inf      20.3      22.8
##  Urethane    20.4 0.632 Inf      19.2      21.7
## 
## genotype = XXM, week =  2:
##  treatment emmean    SE  df asymp.LCL asymp.UCL
##  PBS         22.1 0.790 Inf      20.6      23.7
##  Urethane    20.7 0.752 Inf      19.3      22.2
## 
## genotype = XYF, week =  2:
##  treatment emmean    SE  df asymp.LCL asymp.UCL
##  PBS         21.3 0.729 Inf      19.8      22.7
##  Urethane    20.6 0.792 Inf      19.1      22.2
## 
## genotype = XYM, week =  2:
##  treatment emmean    SE  df asymp.LCL asymp.UCL
##  PBS         21.9 0.773 Inf      20.4      23.5
##  Urethane    20.9 0.836 Inf      19.2      22.5
## 
## genotype = XXF, week =  3:
##  treatment emmean    SE  df asymp.LCL asymp.UCL
##  PBS         22.1 0.643 Inf      20.8      23.3
##  Urethane    20.9 0.632 Inf      19.7      22.1
## 
## genotype = XXM, week =  3:
##  treatment emmean    SE  df asymp.LCL asymp.UCL
##  PBS         23.2 0.790 Inf      21.7      24.8
##  Urethane    21.1 0.752 Inf      19.6      22.6
## 
## genotype = XYF, week =  3:
##  treatment emmean    SE  df asymp.LCL asymp.UCL
##  PBS         22.1 0.729 Inf      20.7      23.6
##  Urethane    21.0 0.792 Inf      19.5      22.6
## 
## genotype = XYM, week =  3:
##  treatment emmean    SE  df asymp.LCL asymp.UCL
##  PBS         22.6 0.773 Inf      21.1      24.1
##  Urethane    21.4 0.836 Inf      19.7      23.0
## 
## genotype = XXF, week =  4:
##  treatment emmean    SE  df asymp.LCL asymp.UCL
##  PBS         22.8 0.643 Inf      21.6      24.1
##  Urethane    21.0 0.632 Inf      19.8      22.2
## 
## genotype = XXM, week =  4:
##  treatment emmean    SE  df asymp.LCL asymp.UCL
##  PBS         24.1 0.790 Inf      22.6      25.7
##  Urethane    21.2 0.752 Inf      19.7      22.6
## 
## genotype = XYF, week =  4:
##  treatment emmean    SE  df asymp.LCL asymp.UCL
##  PBS         22.7 0.729 Inf      21.2      24.1
##  Urethane    21.3 0.792 Inf      19.8      22.9
## 
## genotype = XYM, week =  4:
##  treatment emmean    SE  df asymp.LCL asymp.UCL
##  PBS         23.3 0.773 Inf      21.8      24.8
##  Urethane    21.9 0.836 Inf      20.3      23.6
## 
## genotype = XXF, week =  5:
##  treatment emmean    SE  df asymp.LCL asymp.UCL
##  PBS         23.6 0.643 Inf      22.3      24.8
##  Urethane    21.3 0.632 Inf      20.0      22.5
## 
## genotype = XXM, week =  5:
##  treatment emmean    SE  df asymp.LCL asymp.UCL
##  PBS         25.0 0.790 Inf      23.4      26.5
##  Urethane    21.4 0.752 Inf      19.9      22.8
## 
## genotype = XYF, week =  5:
##  treatment emmean    SE  df asymp.LCL asymp.UCL
##  PBS         23.0 0.729 Inf      21.6      24.5
##  Urethane    21.7 0.792 Inf      20.1      23.3
## 
## genotype = XYM, week =  5:
##  treatment emmean    SE  df asymp.LCL asymp.UCL
##  PBS         24.0 0.773 Inf      22.5      25.5
##  Urethane    22.3 0.836 Inf      20.6      23.9
## 
## genotype = XXF, week =  6:
##  treatment emmean    SE  df asymp.LCL asymp.UCL
##  PBS         24.0 0.643 Inf      22.7      25.2
##  Urethane    21.3 0.632 Inf      20.1      22.5
## 
## genotype = XXM, week =  6:
##  treatment emmean    SE  df asymp.LCL asymp.UCL
##  PBS         26.0 0.790 Inf      24.5      27.6
##  Urethane    21.6 0.752 Inf      20.2      23.1
## 
## genotype = XYF, week =  6:
##  treatment emmean    SE  df asymp.LCL asymp.UCL
##  PBS         23.7 0.729 Inf      22.3      25.2
##  Urethane    21.9 0.792 Inf      20.4      23.5
## 
## genotype = XYM, week =  6:
##  treatment emmean    SE  df asymp.LCL asymp.UCL
##  PBS         24.6 0.773 Inf      23.1      26.2
##  Urethane    22.3 0.836 Inf      20.7      24.0
## 
## genotype = XXF, week =  7:
##  treatment emmean    SE  df asymp.LCL asymp.UCL
##  PBS         24.5 0.643 Inf      23.2      25.7
##  Urethane    21.6 0.632 Inf      20.3      22.8
## 
## genotype = XXM, week =  7:
##  treatment emmean    SE  df asymp.LCL asymp.UCL
##  PBS         27.0 0.790 Inf      25.4      28.5
##  Urethane    21.7 0.752 Inf      20.3      23.2
## 
## genotype = XYF, week =  7:
##  treatment emmean    SE  df asymp.LCL asymp.UCL
##  PBS         24.2 0.729 Inf      22.8      25.6
##  Urethane    22.3 0.792 Inf      20.8      23.9
## 
## genotype = XYM, week =  7:
##  treatment emmean    SE  df asymp.LCL asymp.UCL
##  PBS         25.1 0.773 Inf      23.6      26.6
##  Urethane    22.4 0.836 Inf      20.7      24.0
## 
## genotype = XXF, week =  8:
##  treatment emmean    SE  df asymp.LCL asymp.UCL
##  PBS         25.1 0.643 Inf      23.8      26.4
##  Urethane    21.6 0.632 Inf      20.4      22.9
## 
## genotype = XXM, week =  8:
##  treatment emmean    SE  df asymp.LCL asymp.UCL
##  PBS         27.8 0.790 Inf      26.2      29.3
##  Urethane    21.8 0.752 Inf      20.3      23.3
## 
## genotype = XYF, week =  8:
##  treatment emmean    SE  df asymp.LCL asymp.UCL
##  PBS         24.4 0.729 Inf      23.0      25.8
##  Urethane    22.4 0.792 Inf      20.8      23.9
## 
## genotype = XYM, week =  8:
##  treatment emmean    SE  df asymp.LCL asymp.UCL
##  PBS         25.4 0.773 Inf      23.9      26.9
##  Urethane    22.4 0.836 Inf      20.8      24.1
## 
## genotype = XXF, week =  9:
##  treatment emmean    SE  df asymp.LCL asymp.UCL
##  PBS         25.4 0.643 Inf      24.2      26.7
##  Urethane    21.7 0.632 Inf      20.5      23.0
## 
## genotype = XXM, week =  9:
##  treatment emmean    SE  df asymp.LCL asymp.UCL
##  PBS         28.3 0.790 Inf      26.8      29.9
##  Urethane    21.9 0.752 Inf      20.5      23.4
## 
## genotype = XYF, week =  9:
##  treatment emmean    SE  df asymp.LCL asymp.UCL
##  PBS         24.8 0.729 Inf      23.3      26.2
##  Urethane    22.6 0.792 Inf      21.1      24.2
## 
## genotype = XYM, week =  9:
##  treatment emmean    SE  df asymp.LCL asymp.UCL
##  PBS         25.7 0.773 Inf      24.2      27.2
##  Urethane    22.9 0.836 Inf      21.2      24.5
## 
## genotype = XXF, week = 10:
##  treatment emmean    SE  df asymp.LCL asymp.UCL
##  PBS         25.8 0.643 Inf      24.6      27.1
##  Urethane    21.9 0.632 Inf      20.7      23.2
## 
## genotype = XXM, week = 10:
##  treatment emmean    SE  df asymp.LCL asymp.UCL
##  PBS         28.8 0.790 Inf      27.2      30.3
##  Urethane    22.0 0.752 Inf      20.6      23.5
## 
## genotype = XYF, week = 10:
##  treatment emmean    SE  df asymp.LCL asymp.UCL
##  PBS         25.2 0.729 Inf      23.8      26.6
##  Urethane    22.5 0.792 Inf      21.0      24.1
## 
## genotype = XYM, week = 10:
##  treatment emmean    SE  df asymp.LCL asymp.UCL
##  PBS         26.1 0.773 Inf      24.6      27.6
##  Urethane    22.7 0.836 Inf      21.1      24.3
## 
## genotype = XXF, week = 11:
##  treatment emmean    SE  df asymp.LCL asymp.UCL
##  PBS         26.9 0.643 Inf      25.6      28.2
##  Urethane    22.7 0.632 Inf      21.4      23.9
## 
## genotype = XXM, week = 11:
##  treatment emmean    SE  df asymp.LCL asymp.UCL
##  PBS         29.7 0.790 Inf      28.2      31.3
##  Urethane    22.7 0.752 Inf      21.3      24.2
## 
## genotype = XYF, week = 11:
##  treatment emmean    SE  df asymp.LCL asymp.UCL
##  PBS         25.6 0.729 Inf      24.2      27.0
##  Urethane    23.1 0.792 Inf      21.6      24.7
## 
## genotype = XYM, week = 11:
##  treatment emmean    SE  df asymp.LCL asymp.UCL
##  PBS         26.6 0.773 Inf      25.1      28.1
##  Urethane    23.2 0.836 Inf      21.5      24.8
## 
## genotype = XXF, week = 12:
##  treatment emmean    SE  df asymp.LCL asymp.UCL
##  PBS         27.4 0.643 Inf      26.1      28.7
##  Urethane    23.6 0.632 Inf      22.4      24.9
## 
## genotype = XXM, week = 12:
##  treatment emmean    SE  df asymp.LCL asymp.UCL
##  PBS         30.7 0.790 Inf      29.1      32.2
##  Urethane    23.5 0.752 Inf      22.0      24.9
## 
## genotype = XYF, week = 12:
##  treatment emmean    SE  df asymp.LCL asymp.UCL
##  PBS         26.1 0.729 Inf      24.7      27.5
##  Urethane    23.6 0.792 Inf      22.1      25.2
## 
## genotype = XYM, week = 12:
##  treatment emmean    SE  df asymp.LCL asymp.UCL
##  PBS         27.2 0.773 Inf      25.6      28.7
##  Urethane    24.0 0.836 Inf      22.4      25.6
## 
## genotype = XXF, week = 13:
##  treatment emmean    SE  df asymp.LCL asymp.UCL
##  PBS         28.1 0.643 Inf      26.8      29.3
##  Urethane    23.9 0.632 Inf      22.7      25.2
## 
## genotype = XXM, week = 13:
##  treatment emmean    SE  df asymp.LCL asymp.UCL
##  PBS         31.5 0.790 Inf      30.0      33.1
##  Urethane    24.1 0.752 Inf      22.6      25.5
## 
## genotype = XYF, week = 13:
##  treatment emmean    SE  df asymp.LCL asymp.UCL
##  PBS         26.5 0.729 Inf      25.1      28.0
##  Urethane    24.1 0.792 Inf      22.5      25.6
## 
## genotype = XYM, week = 13:
##  treatment emmean    SE  df asymp.LCL asymp.UCL
##  PBS         27.6 0.773 Inf      26.1      29.1
##  Urethane    24.3 0.836 Inf      22.7      26.0
## 
## genotype = XXF, week = 14:
##  treatment emmean    SE  df asymp.LCL asymp.UCL
##  PBS         28.6 0.643 Inf      27.3      29.8
##  Urethane    23.9 0.632 Inf      22.7      25.1
## 
## genotype = XXM, week = 14:
##  treatment emmean    SE  df asymp.LCL asymp.UCL
##  PBS         32.1 0.790 Inf      30.6      33.7
##  Urethane    24.4 0.752 Inf      23.0      25.9
## 
## genotype = XYF, week = 14:
##  treatment emmean    SE  df asymp.LCL asymp.UCL
##  PBS         26.8 0.729 Inf      25.4      28.2
##  Urethane    24.5 0.792 Inf      22.9      26.0
## 
## genotype = XYM, week = 14:
##  treatment emmean    SE  df asymp.LCL asymp.UCL
##  PBS         28.1 0.773 Inf      26.6      29.6
##  Urethane    24.8 0.836 Inf      23.2      26.5
## 
## genotype = XXF, week = 15:
##  treatment emmean    SE  df asymp.LCL asymp.UCL
##  PBS         29.1 0.643 Inf      27.8      30.3
##  Urethane    24.4 0.632 Inf      23.2      25.7
## 
## genotype = XXM, week = 15:
##  treatment emmean    SE  df asymp.LCL asymp.UCL
##  PBS         32.8 0.790 Inf      31.2      34.3
##  Urethane    24.8 0.752 Inf      23.3      26.3
## 
## genotype = XYF, week = 15:
##  treatment emmean    SE  df asymp.LCL asymp.UCL
##  PBS         27.5 0.729 Inf      26.1      29.0
##  Urethane    24.9 0.792 Inf      23.4      26.5
## 
## genotype = XYM, week = 15:
##  treatment emmean    SE  df asymp.LCL asymp.UCL
##  PBS         28.7 0.773 Inf      27.2      30.2
##  Urethane    25.4 0.836 Inf      23.7      27.0
## 
## genotype = XXF, week = 16:
##  treatment emmean    SE  df asymp.LCL asymp.UCL
##  PBS         29.8 0.643 Inf      28.6      31.1
##  Urethane    24.7 0.632 Inf      23.4      25.9
## 
## genotype = XXM, week = 16:
##  treatment emmean    SE  df asymp.LCL asymp.UCL
##  PBS         33.5 0.790 Inf      31.9      35.0
##  Urethane    25.0 0.752 Inf      23.5      26.5
## 
## genotype = XYF, week = 16:
##  treatment emmean    SE  df asymp.LCL asymp.UCL
##  PBS         28.0 0.729 Inf      26.5      29.4
##  Urethane    25.4 0.792 Inf      23.9      27.0
## 
## genotype = XYM, week = 16:
##  treatment emmean    SE  df asymp.LCL asymp.UCL
##  PBS         28.7 0.773 Inf      27.2      30.2
##  Urethane    25.8 0.836 Inf      24.2      27.5
## 
## genotype = XXF, week = 17:
##  treatment emmean    SE  df asymp.LCL asymp.UCL
##  PBS         30.0 0.643 Inf      28.8      31.3
##  Urethane    25.4 0.632 Inf      24.2      26.7
## 
## genotype = XXM, week = 17:
##  treatment emmean    SE  df asymp.LCL asymp.UCL
##  PBS         33.9 0.790 Inf      32.3      35.4
##  Urethane    25.7 0.752 Inf      24.3      27.2
## 
## genotype = XYF, week = 17:
##  treatment emmean    SE  df asymp.LCL asymp.UCL
##  PBS         28.5 0.729 Inf      27.1      30.0
##  Urethane    26.1 0.792 Inf      24.5      27.6
## 
## genotype = XYM, week = 17:
##  treatment emmean    SE  df asymp.LCL asymp.UCL
##  PBS         28.9 0.773 Inf      27.4      30.4
##  Urethane    26.2 0.836 Inf      24.5      27.8
## 
## genotype = XXF, week = 18:
##  treatment emmean    SE  df asymp.LCL asymp.UCL
##  PBS         30.4 0.643 Inf      29.2      31.7
##  Urethane    25.8 0.632 Inf      24.5      27.0
## 
## genotype = XXM, week = 18:
##  treatment emmean    SE  df asymp.LCL asymp.UCL
##  PBS         34.5 0.790 Inf      32.9      36.0
##  Urethane    26.0 0.752 Inf      24.5      27.4
## 
## genotype = XYF, week = 18:
##  treatment emmean    SE  df asymp.LCL asymp.UCL
##  PBS         29.0 0.729 Inf      27.6      30.5
##  Urethane    26.5 0.792 Inf      24.9      28.0
## 
## genotype = XYM, week = 18:
##  treatment emmean    SE  df asymp.LCL asymp.UCL
##  PBS         29.3 0.773 Inf      27.8      30.8
##  Urethane    26.4 0.836 Inf      24.8      28.1
## 
## genotype = XXF, week = 19:
##  treatment emmean    SE  df asymp.LCL asymp.UCL
##  PBS         31.0 0.643 Inf      29.8      32.3
##  Urethane    26.1 0.632 Inf      24.9      27.4
## 
## genotype = XXM, week = 19:
##  treatment emmean    SE  df asymp.LCL asymp.UCL
##  PBS         35.2 0.790 Inf      33.7      36.8
##  Urethane    26.4 0.752 Inf      25.0      27.9
## 
## genotype = XYF, week = 19:
##  treatment emmean    SE  df asymp.LCL asymp.UCL
##  PBS         29.3 0.729 Inf      27.8      30.7
##  Urethane    26.7 0.792 Inf      25.1      28.3
## 
## genotype = XYM, week = 19:
##  treatment emmean    SE  df asymp.LCL asymp.UCL
##  PBS         29.4 0.773 Inf      27.9      30.9
##  Urethane    27.1 0.836 Inf      25.4      28.7
## 
## genotype = XXF, week = 20:
##  treatment emmean    SE  df asymp.LCL asymp.UCL
##  PBS         31.5 0.643 Inf      30.2      32.7
##  Urethane    26.6 0.632 Inf      25.4      27.8
## 
## genotype = XXM, week = 20:
##  treatment emmean    SE  df asymp.LCL asymp.UCL
##  PBS         35.9 0.790 Inf      34.4      37.5
##  Urethane    26.9 0.752 Inf      25.4      28.3
## 
## genotype = XYF, week = 20:
##  treatment emmean    SE  df asymp.LCL asymp.UCL
##  PBS         29.6 0.729 Inf      28.2      31.0
##  Urethane    27.0 0.792 Inf      25.4      28.5
## 
## genotype = XYM, week = 20:
##  treatment emmean    SE  df asymp.LCL asymp.UCL
##  PBS         29.8 0.773 Inf      28.3      31.3
##  Urethane    27.2 0.836 Inf      25.6      28.9
## 
## genotype = XXF, week = 21:
##  treatment emmean    SE  df asymp.LCL asymp.UCL
##  PBS         31.6 0.643 Inf      30.4      32.9
##  Urethane    27.1 0.632 Inf      25.9      28.3
## 
## genotype = XXM, week = 21:
##  treatment emmean    SE  df asymp.LCL asymp.UCL
##  PBS         36.3 0.790 Inf      34.8      37.9
##  Urethane    27.2 0.752 Inf      25.8      28.7
## 
## genotype = XYF, week = 21:
##  treatment emmean    SE  df asymp.LCL asymp.UCL
##  PBS         30.0 0.729 Inf      28.6      31.5
##  Urethane    27.1 0.792 Inf      25.5      28.6
## 
## genotype = XYM, week = 21:
##  treatment emmean    SE  df asymp.LCL asymp.UCL
##  PBS         30.3 0.773 Inf      28.8      31.8
##  Urethane    27.9 0.836 Inf      26.3      29.5
## 
## genotype = XXF, week = 22:
##  treatment emmean    SE  df asymp.LCL asymp.UCL
##  PBS         32.2 0.643 Inf      30.9      33.5
##  Urethane    28.0 0.632 Inf      26.7      29.2
## 
## genotype = XXM, week = 22:
##  treatment emmean    SE  df asymp.LCL asymp.UCL
##  PBS         36.7 0.790 Inf      35.2      38.3
##  Urethane    27.7 0.752 Inf      26.2      29.2
## 
## genotype = XYF, week = 22:
##  treatment emmean    SE  df asymp.LCL asymp.UCL
##  PBS         30.3 0.729 Inf      28.9      31.7
##  Urethane    27.5 0.792 Inf      25.9      29.0
## 
## genotype = XYM, week = 22:
##  treatment emmean    SE  df asymp.LCL asymp.UCL
##  PBS         30.8 0.773 Inf      29.3      32.3
##  Urethane    28.2 0.836 Inf      26.6      29.8
## 
## genotype = XXF, week = 23:
##  treatment emmean    SE  df asymp.LCL asymp.UCL
##  PBS         32.6 0.643 Inf      31.3      33.8
##  Urethane    28.2 0.632 Inf      26.9      29.4
## 
## genotype = XXM, week = 23:
##  treatment emmean    SE  df asymp.LCL asymp.UCL
##  PBS         37.0 0.790 Inf      35.5      38.6
##  Urethane    28.1 0.752 Inf      26.7      29.6
## 
## genotype = XYF, week = 23:
##  treatment emmean    SE  df asymp.LCL asymp.UCL
##  PBS         30.6 0.729 Inf      29.2      32.0
##  Urethane    27.7 0.792 Inf      26.2      29.3
## 
## genotype = XYM, week = 23:
##  treatment emmean    SE  df asymp.LCL asymp.UCL
##  PBS         30.9 0.773 Inf      29.4      32.4
##  Urethane    28.1 0.836 Inf      26.5      29.7
## 
## genotype = XXF, week = 24:
##  treatment emmean    SE  df asymp.LCL asymp.UCL
##  PBS         33.3 0.643 Inf      32.1      34.6
##  Urethane    29.0 0.632 Inf      27.8      30.3
## 
## genotype = XXM, week = 24:
##  treatment emmean    SE  df asymp.LCL asymp.UCL
##  PBS         37.3 0.790 Inf      35.7      38.8
##  Urethane    28.5 0.752 Inf      27.0      30.0
## 
## genotype = XYF, week = 24:
##  treatment emmean    SE  df asymp.LCL asymp.UCL
##  PBS         30.8 0.729 Inf      29.4      32.3
##  Urethane    28.1 0.792 Inf      26.6      29.7
## 
## genotype = XYM, week = 24:
##  treatment emmean    SE  df asymp.LCL asymp.UCL
##  PBS         31.3 0.773 Inf      29.8      32.8
##  Urethane    28.6 0.836 Inf      27.0      30.2
## 
## genotype = XXF, week = 25:
##  treatment emmean    SE  df asymp.LCL asymp.UCL
##  PBS         33.8 0.643 Inf      32.6      35.1
##  Urethane    29.8 0.632 Inf      28.6      31.1
## 
## genotype = XXM, week = 25:
##  treatment emmean    SE  df asymp.LCL asymp.UCL
##  PBS         37.7 0.790 Inf      36.1      39.2
##  Urethane    28.9 0.752 Inf      27.4      30.4
## 
## genotype = XYF, week = 25:
##  treatment emmean    SE  df asymp.LCL asymp.UCL
##  PBS         31.2 0.729 Inf      29.7      32.6
##  Urethane    28.4 0.792 Inf      26.8      29.9
## 
## genotype = XYM, week = 25:
##  treatment emmean    SE  df asymp.LCL asymp.UCL
##  PBS         31.6 0.773 Inf      30.1      33.1
##  Urethane    29.2 0.836 Inf      27.6      30.9
## 
## genotype = XXF, week = 26:
##  treatment emmean    SE  df asymp.LCL asymp.UCL
##  PBS         34.2 0.643 Inf      32.9      35.5
##  Urethane    30.2 0.632 Inf      29.0      31.4
## 
## genotype = XXM, week = 26:
##  treatment emmean    SE  df asymp.LCL asymp.UCL
##  PBS         38.1 0.790 Inf      36.5      39.6
##  Urethane    29.1 0.752 Inf      27.6      30.5
## 
## genotype = XYF, week = 26:
##  treatment emmean    SE  df asymp.LCL asymp.UCL
##  PBS         31.3 0.729 Inf      29.8      32.7
##  Urethane    28.7 0.792 Inf      27.2      30.3
## 
## genotype = XYM, week = 26:
##  treatment emmean    SE  df asymp.LCL asymp.UCL
##  PBS         31.8 0.773 Inf      30.3      33.3
##  Urethane    29.0 0.836 Inf      27.3      30.6
## 
## genotype = XXF, week = 27:
##  treatment emmean    SE  df asymp.LCL asymp.UCL
##  PBS         35.0 0.643 Inf      33.8      36.3
##  Urethane    31.1 0.632 Inf      29.9      32.4
## 
## genotype = XXM, week = 27:
##  treatment emmean    SE  df asymp.LCL asymp.UCL
##  PBS         38.2 0.790 Inf      36.7      39.8
##  Urethane    29.4 0.752 Inf      28.0      30.9
## 
## genotype = XYF, week = 27:
##  treatment emmean    SE  df asymp.LCL asymp.UCL
##  PBS         31.6 0.729 Inf      30.2      33.1
##  Urethane    28.8 0.792 Inf      27.3      30.4
## 
## genotype = XYM, week = 27:
##  treatment emmean    SE  df asymp.LCL asymp.UCL
##  PBS         31.7 0.773 Inf      30.2      33.2
##  Urethane    28.9 0.836 Inf      27.2      30.5
## 
## genotype = XXF, week = 28:
##  treatment emmean    SE  df asymp.LCL asymp.UCL
##  PBS         35.3 0.643 Inf      34.1      36.6
##  Urethane    31.6 0.632 Inf      30.4      32.9
## 
## genotype = XXM, week = 28:
##  treatment emmean    SE  df asymp.LCL asymp.UCL
##  PBS         38.7 0.790 Inf      37.2      40.3
##  Urethane    29.9 0.752 Inf      28.5      31.4
## 
## genotype = XYF, week = 28:
##  treatment emmean    SE  df asymp.LCL asymp.UCL
##  PBS         32.1 0.729 Inf      30.7      33.6
##  Urethane    29.3 0.792 Inf      27.7      30.8
## 
## genotype = XYM, week = 28:
##  treatment emmean    SE  df asymp.LCL asymp.UCL
##  PBS         32.0 0.773 Inf      30.5      33.6
##  Urethane    29.5 0.836 Inf      27.8      31.1
## 
## genotype = XXF, week = 29:
##  treatment emmean    SE  df asymp.LCL asymp.UCL
##  PBS         35.9 0.643 Inf      34.6      37.2
##  Urethane    32.4 0.632 Inf      31.2      33.6
## 
## genotype = XXM, week = 29:
##  treatment emmean    SE  df asymp.LCL asymp.UCL
##  PBS         38.8 0.790 Inf      37.2      40.3
##  Urethane    30.3 0.752 Inf      28.8      31.8
## 
## genotype = XYF, week = 29:
##  treatment emmean    SE  df asymp.LCL asymp.UCL
##  PBS         32.4 0.729 Inf      31.0      33.8
##  Urethane    29.3 0.792 Inf      27.7      30.8
## 
## genotype = XYM, week = 29:
##  treatment emmean    SE  df asymp.LCL asymp.UCL
##  PBS         32.4 0.773 Inf      30.9      34.0
##  Urethane    30.0 0.836 Inf      28.4      31.6
## 
## genotype = XXF, week = 30:
##  treatment emmean    SE  df asymp.LCL asymp.UCL
##  PBS         35.9 0.643 Inf      34.6      37.2
##  Urethane    32.3 0.632 Inf      31.1      33.6
## 
## genotype = XXM, week = 30:
##  treatment emmean    SE  df asymp.LCL asymp.UCL
##  PBS         38.6 0.790 Inf      37.0      40.1
##  Urethane    29.8 0.752 Inf      28.3      31.3
## 
## genotype = XYF, week = 30:
##  treatment emmean    SE  df asymp.LCL asymp.UCL
##  PBS         32.1 0.729 Inf      30.7      33.6
##  Urethane    29.2 0.792 Inf      27.6      30.7
## 
## genotype = XYM, week = 30:
##  treatment emmean    SE  df asymp.LCL asymp.UCL
##  PBS         32.7 0.773 Inf      31.2      34.2
##  Urethane    29.5 0.836 Inf      27.8      31.1
## 
## Degrees-of-freedom method: asymptotic 
## Confidence level used: 0.95 
## 
## $contrasts
## genotype = XXF, week =  1:
##  contrast        estimate    SE  df z.ratio p.value
##  PBS - Urethane  0.000001 0.857 Inf   0.000  1.0000
## 
## genotype = XXM, week =  1:
##  contrast        estimate    SE  df z.ratio p.value
##  PBS - Urethane  0.001199 0.967 Inf   0.001  0.9990
## 
## genotype = XYF, week =  1:
##  contrast        estimate    SE  df z.ratio p.value
##  PBS - Urethane  0.000480 1.000 Inf   0.000  0.9996
## 
## genotype = XYM, week =  1:
##  contrast        estimate    SE  df z.ratio p.value
##  PBS - Urethane -0.000968 1.110 Inf  -0.001  0.9993
## 
## genotype = XXF, week =  2:
##  contrast        estimate    SE  df z.ratio p.value
##  PBS - Urethane  1.150955 0.857 Inf   1.342  0.1795
## 
## genotype = XXM, week =  2:
##  contrast        estimate    SE  df z.ratio p.value
##  PBS - Urethane  1.400723 0.967 Inf   1.449  0.1473
## 
## genotype = XYF, week =  2:
##  contrast        estimate    SE  df z.ratio p.value
##  PBS - Urethane  0.642147 1.000 Inf   0.640  0.5220
## 
## genotype = XYM, week =  2:
##  contrast        estimate    SE  df z.ratio p.value
##  PBS - Urethane  1.061424 1.110 Inf   0.958  0.3379
## 
## genotype = XXF, week =  3:
##  contrast        estimate    SE  df z.ratio p.value
##  PBS - Urethane  1.180271 0.857 Inf   1.376  0.1687
## 
## genotype = XXM, week =  3:
##  contrast        estimate    SE  df z.ratio p.value
##  PBS - Urethane  2.097866 0.967 Inf   2.170  0.0300
## 
## genotype = XYF, week =  3:
##  contrast        estimate    SE  df z.ratio p.value
##  PBS - Urethane  1.113496 1.000 Inf   1.110  0.2669
## 
## genotype = XYM, week =  3:
##  contrast        estimate    SE  df z.ratio p.value
##  PBS - Urethane  1.272562 1.110 Inf   1.149  0.2505
## 
## genotype = XXF, week =  4:
##  contrast        estimate    SE  df z.ratio p.value
##  PBS - Urethane  1.844388 0.857 Inf   2.151  0.0315
## 
## genotype = XXM, week =  4:
##  contrast        estimate    SE  df z.ratio p.value
##  PBS - Urethane  2.971199 0.967 Inf   3.074  0.0021
## 
## genotype = XYF, week =  4:
##  contrast        estimate    SE  df z.ratio p.value
##  PBS - Urethane  1.312544 1.000 Inf   1.309  0.1906
## 
## genotype = XYM, week =  4:
##  contrast        estimate    SE  df z.ratio p.value
##  PBS - Urethane  1.379032 1.110 Inf   1.245  0.2131
## 
## genotype = XXF, week =  5:
##  contrast        estimate    SE  df z.ratio p.value
##  PBS - Urethane  2.297978 0.857 Inf   2.680  0.0074
## 
## genotype = XXM, week =  5:
##  contrast        estimate    SE  df z.ratio p.value
##  PBS - Urethane  3.595961 0.967 Inf   3.720  0.0002
## 
## genotype = XYF, week =  5:
##  contrast        estimate    SE  df z.ratio p.value
##  PBS - Urethane  1.337068 1.000 Inf   1.333  0.1825
## 
## genotype = XYM, week =  5:
##  contrast        estimate    SE  df z.ratio p.value
##  PBS - Urethane  1.708640 1.110 Inf   1.543  0.1229
## 
## genotype = XXF, week =  6:
##  contrast        estimate    SE  df z.ratio p.value
##  PBS - Urethane  2.663533 0.857 Inf   3.106  0.0019
## 
## genotype = XXM, week =  6:
##  contrast        estimate    SE  df z.ratio p.value
##  PBS - Urethane  4.361199 0.967 Inf   4.512  <.0001
## 
## genotype = XYF, week =  6:
##  contrast        estimate    SE  df z.ratio p.value
##  PBS - Urethane  1.785639 1.000 Inf   1.780  0.0750
## 
## genotype = XYM, week =  6:
##  contrast        estimate    SE  df z.ratio p.value
##  PBS - Urethane  2.304601 1.110 Inf   2.081  0.0374
## 
## genotype = XXF, week =  7:
##  contrast        estimate    SE  df z.ratio p.value
##  PBS - Urethane  2.898776 0.857 Inf   3.381  0.0007
## 
## genotype = XXM, week =  7:
##  contrast        estimate    SE  df z.ratio p.value
##  PBS - Urethane  5.244056 0.967 Inf   5.425  <.0001
## 
## genotype = XYF, week =  7:
##  contrast        estimate    SE  df z.ratio p.value
##  PBS - Urethane  1.870322 1.000 Inf   1.865  0.0622
## 
## genotype = XYM, week =  7:
##  contrast        estimate    SE  df z.ratio p.value
##  PBS - Urethane  2.779189 1.110 Inf   2.509  0.0121
## 
## genotype = XXF, week =  8:
##  contrast        estimate    SE  df z.ratio p.value
##  PBS - Urethane  3.452494 0.857 Inf   4.026  0.0001
## 
## genotype = XXM, week =  8:
##  contrast        estimate    SE  df z.ratio p.value
##  PBS - Urethane  6.011675 0.967 Inf   6.219  <.0001
## 
## genotype = XYF, week =  8:
##  contrast        estimate    SE  df z.ratio p.value
##  PBS - Urethane  2.038576 1.000 Inf   2.033  0.0421
## 
## genotype = XYM, week =  8:
##  contrast        estimate    SE  df z.ratio p.value
##  PBS - Urethane  2.981111 1.110 Inf   2.692  0.0071
## 
## genotype = XXF, week =  9:
##  contrast        estimate    SE  df z.ratio p.value
##  PBS - Urethane  3.681468 0.857 Inf   4.293  <.0001
## 
## genotype = XXM, week =  9:
##  contrast        estimate    SE  df z.ratio p.value
##  PBS - Urethane  6.409771 0.967 Inf   6.631  <.0001
## 
## genotype = XYF, week =  9:
##  contrast        estimate    SE  df z.ratio p.value
##  PBS - Urethane  2.143576 1.000 Inf   2.137  0.0326
## 
## genotype = XYM, week =  9:
##  contrast        estimate    SE  df z.ratio p.value
##  PBS - Urethane  2.859777 1.110 Inf   2.582  0.0098
## 
## genotype = XXF, week = 10:
##  contrast        estimate    SE  df z.ratio p.value
##  PBS - Urethane  3.873348 0.857 Inf   4.517  <.0001
## 
## genotype = XXM, week = 10:
##  contrast        estimate    SE  df z.ratio p.value
##  PBS - Urethane  6.725009 0.967 Inf   6.957  <.0001
## 
## genotype = XYF, week = 10:
##  contrast        estimate    SE  df z.ratio p.value
##  PBS - Urethane  2.660163 1.000 Inf   2.652  0.0080
## 
## genotype = XYM, week = 10:
##  contrast        estimate    SE  df z.ratio p.value
##  PBS - Urethane  3.402915 1.110 Inf   3.073  0.0021
## 
## genotype = XXF, week = 11:
##  contrast        estimate    SE  df z.ratio p.value
##  PBS - Urethane  4.222579 0.857 Inf   4.924  <.0001
## 
## genotype = XXM, week = 11:
##  contrast        estimate    SE  df z.ratio p.value
##  PBS - Urethane  7.010247 0.967 Inf   7.252  <.0001
## 
## genotype = XYF, week = 11:
##  contrast        estimate    SE  df z.ratio p.value
##  PBS - Urethane  2.465798 1.000 Inf   2.459  0.0140
## 
## genotype = XYM, week = 11:
##  contrast        estimate    SE  df z.ratio p.value
##  PBS - Urethane  3.466091 1.110 Inf   3.130  0.0018
## 
## genotype = XXF, week = 12:
##  contrast        estimate    SE  df z.ratio p.value
##  PBS - Urethane  3.779986 0.857 Inf   4.408  <.0001
## 
## genotype = XXM, week = 12:
##  contrast        estimate    SE  df z.ratio p.value
##  PBS - Urethane  7.184056 0.967 Inf   7.432  <.0001
## 
## genotype = XYF, week = 12:
##  contrast        estimate    SE  df z.ratio p.value
##  PBS - Urethane  2.471861 1.000 Inf   2.465  0.0137
## 
## genotype = XYM, week = 12:
##  contrast        estimate    SE  df z.ratio p.value
##  PBS - Urethane  3.155856 1.110 Inf   2.849  0.0044
## 
## genotype = XXF, week = 13:
##  contrast        estimate    SE  df z.ratio p.value
##  PBS - Urethane  4.137679 0.857 Inf   4.825  <.0001
## 
## genotype = XXM, week = 13:
##  contrast        estimate    SE  df z.ratio p.value
##  PBS - Urethane  7.452628 0.967 Inf   7.710  <.0001
## 
## genotype = XYF, week = 13:
##  contrast        estimate    SE  df z.ratio p.value
##  PBS - Urethane  2.469369 1.000 Inf   2.462  0.0138
## 
## genotype = XYM, week = 13:
##  contrast        estimate    SE  df z.ratio p.value
##  PBS - Urethane  3.313150 1.110 Inf   2.992  0.0028
## 
## genotype = XXF, week = 14:
##  contrast        estimate    SE  df z.ratio p.value
##  PBS - Urethane  4.677472 0.857 Inf   5.455  <.0001
## 
## genotype = XXM, week = 14:
##  contrast        estimate    SE  df z.ratio p.value
##  PBS - Urethane  7.683580 0.967 Inf   7.949  <.0001
## 
## genotype = XYF, week = 14:
##  contrast        estimate    SE  df z.ratio p.value
##  PBS - Urethane  2.340877 1.000 Inf   2.334  0.0196
## 
## genotype = XYM, week = 14:
##  contrast        estimate    SE  df z.ratio p.value
##  PBS - Urethane  3.287620 1.110 Inf   2.968  0.0030
## 
## genotype = XXF, week = 15:
##  contrast        estimate    SE  df z.ratio p.value
##  PBS - Urethane  4.642707 0.857 Inf   5.414  <.0001
## 
## genotype = XXM, week = 15:
##  contrast        estimate    SE  df z.ratio p.value
##  PBS - Urethane  7.973580 0.967 Inf   8.249  <.0001
## 
## genotype = XYF, week = 15:
##  contrast        estimate    SE  df z.ratio p.value
##  PBS - Urethane  2.603734 1.000 Inf   2.596  0.0094
## 
## genotype = XYM, week = 15:
##  contrast        estimate    SE  df z.ratio p.value
##  PBS - Urethane  3.337071 1.110 Inf   3.013  0.0026
## 
## genotype = XXF, week = 16:
##  contrast        estimate    SE  df z.ratio p.value
##  PBS - Urethane  5.131824 0.857 Inf   5.985  <.0001
## 
## genotype = XXM, week = 16:
##  contrast        estimate    SE  df z.ratio p.value
##  PBS - Urethane  8.473580 0.967 Inf   8.766  <.0001
## 
## genotype = XYF, week = 16:
##  contrast        estimate    SE  df z.ratio p.value
##  PBS - Urethane  2.530798 1.000 Inf   2.523  0.0116
## 
## genotype = XYM, week = 16:
##  contrast        estimate    SE  df z.ratio p.value
##  PBS - Urethane  2.890483 1.110 Inf   2.610  0.0091
## 
## genotype = XXF, week = 17:
##  contrast        estimate    SE  df z.ratio p.value
##  PBS - Urethane  4.620029 0.857 Inf   5.388  <.0001
## 
## genotype = XXM, week = 17:
##  contrast        estimate    SE  df z.ratio p.value
##  PBS - Urethane  8.136437 0.967 Inf   8.418  <.0001
## 
## genotype = XYF, week = 17:
##  contrast        estimate    SE  df z.ratio p.value
##  PBS - Urethane  2.441274 1.000 Inf   2.434  0.0149
## 
## genotype = XYM, week = 17:
##  contrast        estimate    SE  df z.ratio p.value
##  PBS - Urethane  2.739189 1.110 Inf   2.473  0.0134
## 
## genotype = XXF, week = 18:
##  contrast        estimate    SE  df z.ratio p.value
##  PBS - Urethane  4.676340 0.857 Inf   5.454  <.0001
## 
## genotype = XXM, week = 18:
##  contrast        estimate    SE  df z.ratio p.value
##  PBS - Urethane  8.502151 0.967 Inf   8.796  <.0001
## 
## genotype = XYF, week = 18:
##  contrast        estimate    SE  df z.ratio p.value
##  PBS - Urethane  2.559052 1.000 Inf   2.552  0.0107
## 
## genotype = XYM, week = 18:
##  contrast        estimate    SE  df z.ratio p.value
##  PBS - Urethane  2.854150 1.110 Inf   2.577  0.0100
## 
## genotype = XXF, week = 19:
##  contrast        estimate    SE  df z.ratio p.value
##  PBS - Urethane  4.880092 0.857 Inf   5.691  <.0001
## 
## genotype = XXM, week = 19:
##  contrast        estimate    SE  df z.ratio p.value
##  PBS - Urethane  8.784532 0.967 Inf   9.088  <.0001
## 
## genotype = XYF, week = 19:
##  contrast        estimate    SE  df z.ratio p.value
##  PBS - Urethane  2.553417 1.000 Inf   2.546  0.0109
## 
## genotype = XYM, week = 19:
##  contrast        estimate    SE  df z.ratio p.value
##  PBS - Urethane  2.341581 1.110 Inf   2.114  0.0345
## 
## genotype = XXF, week = 20:
##  contrast        estimate    SE  df z.ratio p.value
##  PBS - Urethane  4.856211 0.857 Inf   5.663  <.0001
## 
## genotype = XXM, week = 20:
##  contrast        estimate    SE  df z.ratio p.value
##  PBS - Urethane  9.040247 0.967 Inf   9.353  <.0001
## 
## genotype = XYF, week = 20:
##  contrast        estimate    SE  df z.ratio p.value
##  PBS - Urethane  2.646830 1.000 Inf   2.639  0.0083
## 
## genotype = XYM, week = 20:
##  contrast        estimate    SE  df z.ratio p.value
##  PBS - Urethane  2.581464 1.110 Inf   2.331  0.0198
## 
## genotype = XXF, week = 21:
##  contrast        estimate    SE  df z.ratio p.value
##  PBS - Urethane  4.528135 0.857 Inf   5.281  <.0001
## 
## genotype = XXM, week = 21:
##  contrast        estimate    SE  df z.ratio p.value
##  PBS - Urethane  9.095485 0.967 Inf   9.410  <.0001
## 
## genotype = XYF, week = 21:
##  contrast        estimate    SE  df z.ratio p.value
##  PBS - Urethane  2.974846 1.000 Inf   2.966  0.0030
## 
## genotype = XYM, week = 21:
##  contrast        estimate    SE  df z.ratio p.value
##  PBS - Urethane  2.434099 1.110 Inf   2.198  0.0280
## 
## genotype = XXF, week = 22:
##  contrast        estimate    SE  df z.ratio p.value
##  PBS - Urethane  4.226667 0.857 Inf   4.929  <.0001
## 
## genotype = XXM, week = 22:
##  contrast        estimate    SE  df z.ratio p.value
##  PBS - Urethane  9.038342 0.967 Inf   9.351  <.0001
## 
## genotype = XYF, week = 22:
##  contrast        estimate    SE  df z.ratio p.value
##  PBS - Urethane  2.831750 1.000 Inf   2.823  0.0048
## 
## genotype = XYM, week = 22:
##  contrast        estimate    SE  df z.ratio p.value
##  PBS - Urethane  2.609620 1.110 Inf   2.356  0.0185
## 
## genotype = XXF, week = 23:
##  contrast        estimate    SE  df z.ratio p.value
##  PBS - Urethane  4.408263 0.857 Inf   5.141  <.0001
## 
## genotype = XXM, week = 23:
##  contrast        estimate    SE  df z.ratio p.value
##  PBS - Urethane  8.917866 0.967 Inf   9.226  <.0001
## 
## genotype = XYF, week = 23:
##  contrast        estimate    SE  df z.ratio p.value
##  PBS - Urethane  2.860560 1.000 Inf   2.852  0.0043
## 
## genotype = XYM, week = 23:
##  contrast        estimate    SE  df z.ratio p.value
##  PBS - Urethane  2.814287 1.110 Inf   2.541  0.0111
## 
## genotype = XXF, week = 24:
##  contrast        estimate    SE  df z.ratio p.value
##  PBS - Urethane  4.273305 0.857 Inf   4.984  <.0001
## 
## genotype = XXM, week = 24:
##  contrast        estimate    SE  df z.ratio p.value
##  PBS - Urethane  8.784532 0.967 Inf   9.088  <.0001
## 
## genotype = XYF, week = 24:
##  contrast        estimate    SE  df z.ratio p.value
##  PBS - Urethane  2.721203 1.000 Inf   2.713  0.0067
## 
## genotype = XYM, week = 24:
##  contrast        estimate    SE  df z.ratio p.value
##  PBS - Urethane  2.735424 1.110 Inf   2.470  0.0135
## 
## genotype = XXF, week = 25:
##  contrast        estimate    SE  df z.ratio p.value
##  PBS - Urethane  4.021596 0.857 Inf   4.690  <.0001
## 
## genotype = XXM, week = 25:
##  contrast        estimate    SE  df z.ratio p.value
##  PBS - Urethane  8.812628 0.967 Inf   9.117  <.0001
## 
## genotype = XYF, week = 25:
##  contrast        estimate    SE  df z.ratio p.value
##  PBS - Urethane  2.793338 1.000 Inf   2.785  0.0054
## 
## genotype = XYM, week = 25:
##  contrast        estimate    SE  df z.ratio p.value
##  PBS - Urethane  2.350091 1.110 Inf   2.122  0.0338
## 
## genotype = XXF, week = 26:
##  contrast        estimate    SE  df z.ratio p.value
##  PBS - Urethane  4.014773 0.857 Inf   4.682  <.0001
## 
## genotype = XXM, week = 26:
##  contrast        estimate    SE  df z.ratio p.value
##  PBS - Urethane  9.008342 0.967 Inf   9.320  <.0001
## 
## genotype = XYF, week = 26:
##  contrast        estimate    SE  df z.ratio p.value
##  PBS - Urethane  2.536671 1.000 Inf   2.529  0.0114
## 
## genotype = XYM, week = 26:
##  contrast        estimate    SE  df z.ratio p.value
##  PBS - Urethane  2.830483 1.110 Inf   2.556  0.0106
## 
## genotype = XXF, week = 27:
##  contrast        estimate    SE  df z.ratio p.value
##  PBS - Urethane  3.907437 0.857 Inf   4.557  <.0001
## 
## genotype = XXM, week = 27:
##  contrast        estimate    SE  df z.ratio p.value
##  PBS - Urethane  8.808342 0.967 Inf   9.113  <.0001
## 
## genotype = XYF, week = 27:
##  contrast        estimate    SE  df z.ratio p.value
##  PBS - Urethane  2.815512 1.000 Inf   2.807  0.0050
## 
## genotype = XYM, week = 27:
##  contrast        estimate    SE  df z.ratio p.value
##  PBS - Urethane  2.796091 1.110 Inf   2.525  0.0116
## 
## genotype = XXF, week = 28:
##  contrast        estimate    SE  df z.ratio p.value
##  PBS - Urethane  3.709531 0.857 Inf   4.326  <.0001
## 
## genotype = XXM, week = 28:
##  contrast        estimate    SE  df z.ratio p.value
##  PBS - Urethane  8.788818 0.967 Inf   9.092  <.0001
## 
## genotype = XYF, week = 28:
##  contrast        estimate    SE  df z.ratio p.value
##  PBS - Urethane  2.844211 1.000 Inf   2.836  0.0046
## 
## genotype = XYM, week = 28:
##  contrast        estimate    SE  df z.ratio p.value
##  PBS - Urethane  2.580170 1.110 Inf   2.330  0.0198
## 
## genotype = XXF, week = 29:
##  contrast        estimate    SE  df z.ratio p.value
##  PBS - Urethane  3.494274 0.857 Inf   4.075  <.0001
## 
## genotype = XXM, week = 29:
##  contrast        estimate    SE  df z.ratio p.value
##  PBS - Urethane  8.457390 0.967 Inf   8.750  <.0001
## 
## genotype = XYF, week = 29:
##  contrast        estimate    SE  df z.ratio p.value
##  PBS - Urethane  3.162068 1.000 Inf   3.153  0.0016
## 
## genotype = XYM, week = 29:
##  contrast        estimate    SE  df z.ratio p.value
##  PBS - Urethane  2.443738 1.110 Inf   2.207  0.0273
## 
## genotype = XXF, week = 30:
##  contrast        estimate    SE  df z.ratio p.value
##  PBS - Urethane  3.588989 0.857 Inf   4.185  <.0001
## 
## genotype = XXM, week = 30:
##  contrast        estimate    SE  df z.ratio p.value
##  PBS - Urethane  8.758342 0.967 Inf   9.061  <.0001
## 
## genotype = XYF, week = 30:
##  contrast        estimate    SE  df z.ratio p.value
##  PBS - Urethane  2.931115 1.000 Inf   2.922  0.0035
## 
## genotype = XYM, week = 30:
##  contrast        estimate    SE  df z.ratio p.value
##  PBS - Urethane  3.232170 1.110 Inf   2.918  0.0035
## 
## Degrees-of-freedom method: asymptotic
```

Below is a plot of the above table of estimated weight values.

Because there are too many significant differences at each week, I am
again using significance stars to make differences between treatments at
each week. This time, because there are only two groups being compared,
I am using usual p-value star cutoffs: \* = p < 0.05, \*\* = p <
0.01, \*\*\* = p < 0.001.

PBS generally shows greater weight than Urethane in all genetic
backgrounds, but the difference is more or less significant in the four
genotypes across time.

```
# Compute emmeans with pairwise comparisons
emm <- emmeans(model, pairwise ~ treatment | genotype * week)

# Extract the estimated marginal means (EMMs)
emm_df <- as.data.frame(emm$emmeans) 

# Extract the pairwise comparisons
pwc <- as.data.frame(emm$contrasts)

# Add y-position for p-value annotations
pwc <- pwc %>%
    group_by(genotype, week) %>%
    mutate(y.position = max(emm_df$asymp.UCL[emm_df$genotype == genotype &
                                              emm_df$week == week]) + 
               seq(0.3, 0.3, length.out = n())) %>%
    ungroup()
    # mutate(p.value = ifelse(p.value > 0.05, "", format.pval(p.value, eps = 0.001, digits = 1)))

# Create the plot
p <- ggplot(emm_df, aes(x = week, y = emmean, color = treatment)) +
    geom_point(position = position_dodge(width = 0.1), size = 3) +
    geom_errorbar(aes(ymin = asymp.LCL, ymax = asymp.UCL), 
                  width = 0.2, position = position_dodge(width = 0.1)) +
    geom_line(aes(group = treatment)) +  # make a trend line across the weeks
    facet_wrap(~ genotype) + # facet the plots by one grouping variable
    theme_bw() + # use a simple BW theme
    labs(title = paste("Effect plot of expected weight, by genotype, treatment across time"), y = paste("Predicted value of weight"), x = "Week", color = "Treatment")

# format data for stat_pvalue
pwc <- pwc %>%
    mutate(
        treat1 = sub(" - .*", "", contrast),
        treat2 = sub(".* - ", "", contrast),
        y.position = max(emm_df$asymp.UCL) + seq(0.3, 0.3, length.out = n()),
        group1 = paste(genotype, treat1, sep = "-"),
        group2 = paste(genotype, treat2, sep = "-")
    ) %>%
  group_by(genotype, week) %>%
  mutate(p_val_star = ifelse(as.numeric(p.value) < 0.05, "*", ""),
         p_val_star = ifelse(as.numeric(p.value) < 0.01, "**", p_val_star),
         p_val_star = ifelse(as.numeric(p.value) < 0.001, "***", p_val_star)) %>% # too many significant weeks, just adding a star instead if there are any pairwise significant groups.
  ungroup()
    # mutate(p.value = ifelse(p.value != "",
    #                         paste(group1, "vs.", group2, " p = ", p.value),
    #                         ""))


# Add p-values and display the plot
final_plot <- p + stat_pvalue_manual(pwc, 
                       x = "week",
                       label = "p_val_star", 
                       y.position = "y.position",
                       tip.length = 0.01,
                       step.increase = 0.5,
                       label.size = 4)


print(final_plot)
```

# Lung weights

## Descriptive plots

Boxplots showing lung weight (g) and normalized lung weight (mg /g)
for each genotype and colored by treatment

```
lung_weight_data %>%
    ggplot(aes(x = genotype, y = lung_weight_g, color = treatment)) +
    geom_boxplot(outliers = FALSE) +
    geom_point(alpha = 0.3, 
               position = position_dodge(width = .8)) +
    theme_minimal() +
    labs(x = "Genotype", y = "Lung weight (g)", color = "Treatment")
```

```
lung_weight_data %>%
    ggplot(aes(x = genotype, y = mg_g, color = treatment)) +
    geom_boxplot(outliers = FALSE) +
    geom_point(alpha = 0.3, 
               position = position_dodge(width = .8)) +
    theme_minimal() +
    labs(x = "Genotype", y = "Normalized lung weight (mg lungs/g bodyweight)", color = "Treatment")
```

## Linear model analysis (ANOVA)

Below we are fitting linear models examining differences in lung
weight (g) and normalized lung weight (mg / g) between different
genotypes and treatments. This analysis approach is analogous to the
two-way ANOVA that was used previously. With this approach we will first
examine the type 3 anova from each model (as before) to determine if the
main effects or their interaction is significant. And then we will also
view the estimated marginal means for each model to see if there are any
significant pairwise differences between the treatments or
genotypes.

### Lung weight (g)

First we are looking at the un-normalized, lung weight (in grams)
variable as our outcome.

This model shows that the interaction between genotype and treatment
is *not* significant.

#### Model significance (anova table)

```
model_g <- lung_weight_data %>%
  lm(lung_weight_g ~ genotype * treatment, data = .)

print(car::Anova(model_g, type = 2))
```

```
## Anova Table (Type II tests)
## 
## Response: lung_weight_g
##                     Sum Sq Df F value   Pr(>F)    
## genotype           0.02881  3  1.1399 0.339377    
## treatment          0.11625  1 13.7976 0.000417 ***
## genotype:treatment 0.02298  3  0.9093 0.441319    
## Residuals          0.56449 67                     
## ---
## Signif. codes:  0 '***' 0.001 '**' 0.01 '*' 0.05 '.' 0.1 ' ' 1
```

#### Estimated marginal means

##### Genotype within treatment

No significant differences between genotypes within either
treatment.

```
summary(emmeans(model_g, pairwise ~ genotype | treatment))
```

```
## $emmeans
## treatment = PBS:
##  genotype emmean     SE df lower.CL upper.CL
##  XXF       0.388 0.0306 67    0.327    0.449
##  XXM       0.402 0.0306 67    0.341    0.463
##  XYF       0.347 0.0306 67    0.286    0.408
##  XYM       0.364 0.0290 67    0.306    0.422
## 
## treatment = Urethane:
##  genotype emmean     SE df lower.CL upper.CL
##  XXF       0.410 0.0277 67    0.354    0.465
##  XXM       0.493 0.0325 67    0.428    0.558
##  XYF       0.452 0.0265 67    0.399    0.505
##  XYM       0.469 0.0347 67    0.399    0.538
## 
## Confidence level used: 0.95 
## 
## $contrasts
## treatment = PBS:
##  contrast  estimate     SE df t.ratio p.value
##  XXF - XXM  -0.0146 0.0433 67  -0.337  0.9867
##  XXF - XYF   0.0409 0.0433 67   0.944  0.7812
##  XXF - XYM   0.0239 0.0422 67   0.566  0.9418
##  XXM - XYF   0.0554 0.0433 67   1.281  0.5779
##  XXM - XYM   0.0385 0.0422 67   0.912  0.7986
##  XYF - XYM  -0.0170 0.0422 67  -0.403  0.9777
## 
## treatment = Urethane:
##  contrast  estimate     SE df t.ratio p.value
##  XXF - XXM  -0.0835 0.0427 67  -1.959  0.2140
##  XXF - XYF  -0.0423 0.0383 67  -1.104  0.6882
##  XXF - XYM  -0.0591 0.0444 67  -1.332  0.5460
##  XXM - XYF   0.0412 0.0419 67   0.984  0.7589
##  XXM - XYM   0.0244 0.0475 67   0.514  0.9555
##  XYF - XYM  -0.0168 0.0437 67  -0.385  0.9804
## 
## P value adjustment: tukey method for comparing a family of 4 estimates
```

Below is a plot of the above table of estimated lung weight
values.

This time there are no significant differences between the genotypes
within the treatments.

```
# Compute emmeans with pairwise comparisons
emm <- emmeans(model_g, pairwise ~ genotype | treatment)

# Extract the estimated marginal means (EMMs)
emm_df <- as.data.frame(emm$emmeans) 

# Extract the pairwise comparisons
pwc <- as.data.frame(emm$contrasts)

# Add y-position for p-value annotations
pwc <- pwc %>%
    group_by(treatment) %>%
    mutate(y.position = max(emm_df$upper.CL[emm_df$treatment == treatment]) + 
               seq(0.3, 0.3, length.out = n())) %>%
    ungroup() %>%
    mutate(p.value = ifelse(p.value > 0.05, "", format.pval(p.value, eps = 0.001, digits = 1)))

# Create the plot
p <- ggplot(emm_df, aes(x = treatment, y = emmean, color = genotype)) +
    geom_point(position = position_dodge(width = 0.1), size = 3) +
    geom_errorbar(aes(ymin = lower.CL, ymax = upper.CL), 
                  width = 0.2, position = position_dodge(width = 0.1)) +
    theme_bw() + # use a simple BW theme
    labs(title = paste("Effect plot of expected lung weight, by genotype, and treatment"), y = paste("Predicted value of lung weight (g)"), x = "Treatment", color = "Genotype")

# format data for stat_pvalue
pwc <- pwc %>%
    mutate(
        treat1 = sub(" - .*", "", contrast),
        treat2 = sub(".* - ", "", contrast),
        y.position = max(emm_df$upper.CL) + seq(0.3, 0.3, length.out = n()),
        group1 = paste(treatment, treat1, sep = "-"),
        group2 = paste(treatment, treat2, sep = "-")
    ) %>%
    mutate(p.value = ifelse(p.value != "",
                            paste(group1, "vs.", group2, " p = ", p.value),
                            ""))


# Add p-values and display the plot
# none significant here, so this time not doing this
# final_plot <- p + stat_pvalue_manual(pwc, 
#                        x = "treatment",
#                        label = "p.value", 
#                        y.position = "y.position",
#                        tip.length = 0.01,
#                        step.increase = 0.5,
#                        label.size = 4)


# print(final_plot)

print(p)
```

##### Treatment within genotype

We see that there are significant differences between treatments
within 3 of the 4 genotypes.

```
emmeans(model_g, pairwise ~ treatment | genotype)
```

```
## $emmeans
## genotype = XXF:
##  treatment emmean     SE df lower.CL upper.CL
##  PBS        0.388 0.0306 67    0.327    0.449
##  Urethane   0.410 0.0277 67    0.354    0.465
## 
## genotype = XXM:
##  treatment emmean     SE df lower.CL upper.CL
##  PBS        0.402 0.0306 67    0.341    0.463
##  Urethane   0.493 0.0325 67    0.428    0.558
## 
## genotype = XYF:
##  treatment emmean     SE df lower.CL upper.CL
##  PBS        0.347 0.0306 67    0.286    0.408
##  Urethane   0.452 0.0265 67    0.399    0.505
## 
## genotype = XYM:
##  treatment emmean     SE df lower.CL upper.CL
##  PBS        0.364 0.0290 67    0.306    0.422
##  Urethane   0.469 0.0347 67    0.399    0.538
## 
## Confidence level used: 0.95 
## 
## $contrasts
## genotype = XXF:
##  contrast       estimate     SE df t.ratio p.value
##  PBS - Urethane  -0.0218 0.0413 67  -0.529  0.5983
## 
## genotype = XXM:
##  contrast       estimate     SE df t.ratio p.value
##  PBS - Urethane  -0.0908 0.0446 67  -2.036  0.0457
## 
## genotype = XYF:
##  contrast       estimate     SE df t.ratio p.value
##  PBS - Urethane  -0.1050 0.0405 67  -2.594  0.0116
## 
## genotype = XYM:
##  contrast       estimate     SE df t.ratio p.value
##  PBS - Urethane  -0.1048 0.0452 67  -2.317  0.0235
```

Below is a plot of the above table of estimated weight values.

We see that animals treated with Urethane have higher average,
expected, lung weights (in g) in the XXM, XYF, and XYM genotypes.

```
# Compute emmeans with pairwise comparisons
emm <- emmeans(model_g, pairwise ~ treatment | genotype)

# Extract the estimated marginal means (EMMs)
emm_df <- as.data.frame(emm$emmeans) 

# Extract the pairwise comparisons
pwc <- as.data.frame(emm$contrasts)

# Add y-position for p-value annotations
pwc <- pwc %>%
    group_by(genotype) %>%
    mutate(y.position = max(emm_df$upper.CL[emm_df$genotype == genotype]) + 
               seq(0.01, 0.1, length.out = n())) %>%
    ungroup() %>%
    mutate(p.value = ifelse(p.value > 0.05, "", format.pval(p.value, eps = 0.001, digits = 1)))

# Create the plot
p <- ggplot(emm_df, aes(x = genotype, y = emmean, color = treatment)) +
    geom_point(position = position_dodge(width = 0.1), size = 3) +
    geom_errorbar(aes(ymin = lower.CL, ymax = upper.CL), 
                  width = 0.2, position = position_dodge(width = 0.1)) +
    theme_bw() + # use a simple BW theme
    labs(title = paste("Effect plot of expected lung weight, by genotype, and treatment"), y = paste("Predicted value of lung weight (g)"), x = "Genotype", color = "Treatment")

# format data for stat_pvalue
pwc <- pwc %>%
    mutate(
        treat1 = sub(" - .*", "", contrast),
        treat2 = sub(".* - ", "", contrast),
        group1 = paste(genotype, treat1, sep = "-"),
        group2 = paste(genotype, treat2, sep = "-")
    )
    # mutate(p.value = ifelse(p.value != "",
    #                         paste(group1, "vs.", group2, " p = ", p.value),
    #                         ""))


# Add p-values and display the plot
final_plot <- p + stat_pvalue_manual(pwc, 
                       x = "genotype",
                       label = "p.value", 
                       y.position = "y.position",
                       tip.length = 0.01,
                       step.increase = 0.5,
                       label.size = 4)


print(final_plot)
```

### Normalized lung weight (mg / g)

Next we are looking at the normalized, lung weight (in milligrams of
lung per gram of body weight) variable as our outcome.

Again we see that the interaction between genotype and treatment is
*not* significant.

#### Model significance (anova table)

```
model_mg <- lung_weight_data %>%
  lm(mg_g ~ genotype * treatment, data = .)

print(car::Anova(model_mg, type = 2))
```

```
## Anova Table (Type II tests)
## 
## Response: mg_g
##                    Sum Sq Df F value    Pr(>F)    
## genotype            68.30  3  1.8335 0.1494725    
## treatment          190.27  1 15.3222 0.0002147 ***
## genotype:treatment  73.42  3  1.9708 0.1267278    
## Residuals          831.99 67                      
## ---
## Signif. codes:  0 '***' 0.001 '**' 0.01 '*' 0.05 '.' 0.1 ' ' 1
```

#### Estimated marginal means

##### Genotype within treatment

No significant differences between genotypes within either
treatment.

```
summary(emmeans(model_mg, pairwise ~ genotype | treatment))
```

```
## $emmeans
## treatment = PBS:
##  genotype emmean   SE df lower.CL upper.CL
##  XXF        13.0 1.17 67    10.64     15.3
##  XXM        10.4 1.17 67     8.08     12.8
##  XYF        13.5 1.17 67    11.13     15.8
##  XYM        11.3 1.11 67     9.08     13.5
## 
## treatment = Urethane:
##  genotype emmean   SE df lower.CL upper.CL
##  XXF        13.1 1.06 67    10.97     15.2
##  XXM        15.7 1.25 67    13.20     18.2
##  XYF        16.9 1.02 67    14.83     18.9
##  XYM        15.9 1.33 67    13.26     18.6
## 
## Confidence level used: 0.95 
## 
## $contrasts
## treatment = PBS:
##  contrast  estimate   SE df t.ratio p.value
##  XXF - XXM    2.551 1.66 67   1.535  0.4224
##  XXF - XYF   -0.492 1.66 67  -0.296  0.9909
##  XXF - XYM    1.671 1.62 67   1.032  0.7313
##  XXM - XYF   -3.042 1.66 67  -1.831  0.2679
##  XXM - XYM   -0.880 1.62 67  -0.543  0.9480
##  XYF - XYM    2.163 1.62 67   1.336  0.5438
## 
## treatment = Urethane:
##  contrast  estimate   SE df t.ratio p.value
##  XXF - XXM   -2.594 1.64 67  -1.584  0.3943
##  XXF - XYF   -3.770 1.47 67  -2.563  0.0595
##  XXF - XYM   -2.820 1.70 67  -1.655  0.3553
##  XXM - XYF   -1.176 1.61 67  -0.731  0.8844
##  XXM - XYM   -0.226 1.82 67  -0.124  0.9993
##  XYF - XYM    0.950 1.68 67   0.567  0.9416
## 
## P value adjustment: tukey method for comparing a family of 4 estimates
```

Below is a plot of the above table of estimated lung weight
values.

This time there are no significant differences between the genotypes
within the treatments.

```
# Compute emmeans with pairwise comparisons
emm <- emmeans(model_mg, pairwise ~ genotype | treatment)

# Extract the estimated marginal means (EMMs)
emm_df <- as.data.frame(emm$emmeans) 

# Extract the pairwise comparisons
pwc <- as.data.frame(emm$contrasts)

# Add y-position for p-value annotations
pwc <- pwc %>%
    group_by(treatment) %>%
    mutate(y.position = max(emm_df$upper.CL[emm_df$treatment == treatment]) + 
               seq(0.3, 0.3, length.out = n())) %>%
    ungroup() %>%
    mutate(p.value = ifelse(p.value > 0.05, "", format.pval(p.value, eps = 0.001, digits = 1)))

# Create the plot
p <- ggplot(emm_df, aes(x = treatment, y = emmean, color = genotype)) +
    geom_point(position = position_dodge(width = 0.1), size = 3) +
    geom_errorbar(aes(ymin = lower.CL, ymax = upper.CL), 
                  width = 0.2, position = position_dodge(width = 0.1)) +
    theme_bw() + # use a simple BW theme
    labs(title = paste("Effect plot of expected normalized lung weight, by genotype, and treatment"), y = paste("Predicted value of normalized lung weight (mg / g"), x = "Treatment", color = "Genotype")

# format data for stat_pvalue
pwc <- pwc %>%
    mutate(
        treat1 = sub(" - .*", "", contrast),
        treat2 = sub(".* - ", "", contrast),
        y.position = max(emm_df$upper.CL) + seq(0.3, 0.3, length.out = n()),
        group1 = paste(treatment, treat1, sep = "-"),
        group2 = paste(treatment, treat2, sep = "-")
    ) %>%
    mutate(p.value = ifelse(p.value != "",
                            paste(group1, "vs.", group2, " p = ", p.value),
                            ""))


# Add p-values and display the plot
# none significant here, so this time not doing this
# final_plot <- p + stat_pvalue_manual(pwc, 
#                        x = "treatment",
#                        label = "p.value", 
#                        y.position = "y.position",
#                        tip.length = 0.01,
#                        step.increase = 0.5,
#                        label.size = 4)


# print(final_plot)

print(p)
```

##### Treatment within genotype

We see that there are significant differences between treatments
within 3 of the 4 genotypes.

```
emmeans(model_mg, pairwise ~ treatment | genotype)
```

```
## $emmeans
## genotype = XXF:
##  treatment emmean   SE df lower.CL upper.CL
##  PBS         13.0 1.17 67    10.64     15.3
##  Urethane    13.1 1.06 67    10.97     15.2
## 
## genotype = XXM:
##  treatment emmean   SE df lower.CL upper.CL
##  PBS         10.4 1.17 67     8.08     12.8
##  Urethane    15.7 1.25 67    13.20     18.2
## 
## genotype = XYF:
##  treatment emmean   SE df lower.CL upper.CL
##  PBS         13.5 1.17 67    11.13     15.8
##  Urethane    16.9 1.02 67    14.83     18.9
## 
## genotype = XYM:
##  treatment emmean   SE df lower.CL upper.CL
##  PBS         11.3 1.11 67     9.08     13.5
##  Urethane    15.9 1.33 67    13.26     18.6
## 
## Confidence level used: 0.95 
## 
## $contrasts
## genotype = XXF:
##  contrast       estimate   SE df t.ratio p.value
##  PBS - Urethane   -0.114 1.58 67  -0.072  0.9430
## 
## genotype = XXM:
##  contrast       estimate   SE df t.ratio p.value
##  PBS - Urethane   -5.259 1.71 67  -3.071  0.0031
## 
## genotype = XYF:
##  contrast       estimate   SE df t.ratio p.value
##  PBS - Urethane   -3.392 1.55 67  -2.183  0.0325
## 
## genotype = XYM:
##  contrast       estimate   SE df t.ratio p.value
##  PBS - Urethane   -4.605 1.74 67  -2.652  0.0100
```

Below is a plot of the above table of estimated weight values.

We see that animals treated with Urethane have higher average,
expected, lung weights (in g) in the XXM, XYF, and XYM genotypes.

```
# Compute emmeans with pairwise comparisons
emm <- emmeans(model_mg, pairwise ~ treatment | genotype)

# Extract the estimated marginal means (EMMs)
emm_df <- as.data.frame(emm$emmeans) 

# Extract the pairwise comparisons
pwc <- as.data.frame(emm$contrasts)

# Add y-position for p-value annotations
pwc <- pwc %>%
    group_by(genotype) %>%
    mutate(y.position = max(emm_df$upper.CL[emm_df$genotype == genotype]) + 
               seq(0.1, 0.2, length.out = n())) %>%
    ungroup() %>%
    mutate(p.value = ifelse(p.value > 0.05, "", format.pval(p.value, eps = 0.001, digits = 1)))

# Create the plot
p <- ggplot(emm_df, aes(x = genotype, y = emmean, color = treatment)) +
    geom_point(position = position_dodge(width = 0.1), size = 3) +
    geom_errorbar(aes(ymin = lower.CL, ymax = upper.CL), 
                  width = 0.2, position = position_dodge(width = 0.1)) +
    theme_bw() + # use a simple BW theme
    labs(title = paste("Effect plot of expected normalized lung weight, by genotype, and treatment"), y = paste("Predicted value of normalized lung weight (mg / g)"), x = "Genotype", color = "Treatment")

# format data for stat_pvalue
pwc <- pwc %>%
    mutate(
        treat1 = sub(" - .*", "", contrast),
        treat2 = sub(".* - ", "", contrast),
        group1 = paste(genotype, treat1, sep = "-"),
        group2 = paste(genotype, treat2, sep = "-")
    )
    # mutate(p.value = ifelse(p.value != "",
    #                         paste(group1, "vs.", group2, " p = ", p.value),
    #                         ""))


# Add p-values and display the plot
final_plot <- p + stat_pvalue_manual(pwc, 
                       x = "genotype",
                       label = "p.value", 
                       y.position = "y.position",
                       tip.length = 0.01,
                       step.increase = 0.5,
                       label.size = 4)


print(final_plot)
```

# Tumor data

Because there are many more variables in this dataset, we will first
look at a table of all of them, and look for differences between
genotype groups. Based on the datasets description document, we will
then look at models for tumor counts (total\_foci), and tumor areas (area
and ratio).

## Descriptive table

No apparent differences between genotypes in any of the variables

```
tumor_data %>%
  select(genotype:ki_67_cells_per_mm2) %>%
  tbl_summary(by = genotype, # stratify table by genotype group
              missing_text = "Missing", 
                type = c(all_dichotomous() ~ "categorical",
                         all_continuous() ~ "continuous2"),
              statistic = list(all_continuous() ~ c("{mean} ({sd})", "{median} ({p25}, {p75})", "{min}, {max}"))) %>%
  bold_labels() %>%
  add_overall(last = T) %>% # add overall column
  add_p(pvalue_fun = label_style_pvalue(digits = 3)) # add p-value
```

| **Characteristic** | **XXF**  N = 81 | **XXM**  N = 101 | **XYF**  N = 91 | **XYM**  N = 101 | **Overall**  N = 371 | **p-value**2 |
| --- | --- | --- | --- | --- | --- | --- |
| sections |  |  |  |  |  | 0.536 |
| 3 | 0 (0%) | 1 (10%) | 0 (0%) | 0 (0%) | 1 (2.7%) |  |
| 4 | 0 (0%) | 1 (10%) | 3 (33%) | 1 (10%) | 5 (14%) |  |
| 5 | 3 (38%) | 5 (50%) | 4 (44%) | 6 (60%) | 18 (49%) |  |
| 6 | 5 (63%) | 3 (30%) | 2 (22%) | 3 (30%) | 13 (35%) |  |
| total\_foci |  |  |  |  |  | 0.268 |
| Mean (SD) | 6.3 (2.0) | 6.5 (3.0) | 8.7 (3.9) | 5.6 (2.7) | 6.7 (3.1) |  |
| Median (Q1, Q3) | 6.5 (5.0, 7.5) | 5.5 (4.0, 8.0) | 7.0 (6.0, 12.0) | 4.5 (4.0, 7.0) | 6.0 (4.0, 8.0) |  |
| Min, Max | 3.0, 9.0 | 3.0, 13.0 | 3.0, 15.0 | 3.0, 11.0 | 3.0, 15.0 |  |
| ad |  |  |  |  |  | 0.254 |
| Mean (SD) | 6.13 (1.96) | 6.40 (2.99) | 8.33 (3.84) | 5.40 (2.76) | 6.54 (3.06) |  |
| Median (Q1, Q3) | 6.00 (5.00, 7.50) | 5.50 (4.00, 8.00) | 7.00 (6.00, 12.00) | 4.50 (3.00, 7.00) | 6.00 (4.00, 8.00) |  |
| Min, Max | 3.00, 9.00 | 3.00, 13.00 | 3.00, 15.00 | 3.00, 11.00 | 3.00, 15.00 |  |
| ac |  |  |  |  |  |  |
| 1 | 1 (100%) | 1 (100%) | 3 (100%) | 2 (100%) | 7 (100%) |  |
| Missing | 7 | 9 | 6 | 8 | 30 |  |
| lung\_area\_mm2 |  |  |  |  |  | 0.157 |
| Mean (SD) | 476 (80) | 396 (79) | 432 (122) | 392 (84) | 421 (95) |  |
| Median (Q1, Q3) | 473 (422, 548) | 391 (369, 441) | 423 (403, 509) | 374 (349, 488) | 408 (356, 491) |  |
| Min, Max | 346, 578 | 245, 544 | 235, 612 | 248, 516 | 235, 612 |  |
| tumor\_area\_mm2 |  |  |  |  |  | 0.340 |
| Mean (SD) | 2.5 (1.7) | 7.2 (12.3) | 6.2 (13.4) | 2.0 (1.2) | 4.5 (9.2) |  |
| Median (Q1, Q3) | 2.9 (0.8, 3.5) | 3.2 (1.7, 3.5) | 1.3 (1.0, 2.6) | 1.8 (1.0, 3.0) | 2.3 (1.1, 3.4) |  |
| Min, Max | 0.5, 5.1 | 1.3, 41.3 | 0.5, 41.8 | 0.6, 4.1 | 0.5, 41.8 |  |
| ratio |  |  |  |  |  | 0.230 |
| Mean (SD) | 0.005 (0.004) | 0.018 (0.030) | 0.015 (0.033) | 0.006 (0.004) | 0.011 (0.023) |  |
| Median (Q1, Q3) | 0.006 (0.002, 0.008) | 0.007 (0.005, 0.009) | 0.003 (0.002, 0.005) | 0.005 (0.003, 0.007) | 0.005 (0.003, 0.008) |  |
| Min, Max | 0.001, 0.010 | 0.003, 0.101 | 0.001, 0.103 | 0.002, 0.016 | 0.001, 0.103 |  |
| ki\_67\_percent |  |  |  |  |  | 0.370 |
| Mean (SD) | 10.1 (9.9) | 8.3 (2.3) | 7.8 (4.6) | 5.6 (1.1) | 7.9 (5.3) |  |
| Median (Q1, Q3) | 5.7 (5.0, 7.6) | 8.6 (6.7, 10.6) | 6.2 (5.4, 7.7) | 5.5 (4.6, 6.4) | 6.4 (5.0, 8.3) |  |
| Min, Max | 4.5, 27.7 | 4.6, 10.6 | 4.3, 17.0 | 4.5, 7.1 | 4.3, 27.7 |  |
| Missing | 3 | 4 | 3 | 5 | 15 |  |
| ki\_67\_cells\_per\_mm2 |  |  |  |  |  | 0.524 |
| Mean (SD) | 985 (1,043) | 748 (222) | 718 (317) | 555 (127) | 750 (518) |  |
| Median (Q1, Q3) | 542 (417, 750) | 780 (553, 966) | 622 (575, 693) | 507 (451, 661) | 622 (451, 750) |  |
| Min, Max | 384, 2,832 | 429, 981 | 451, 1,343 | 438, 718 | 384, 2,832 |  |
| Missing | 3 | 4 | 3 | 5 | 15 |  |
|  |  |  |  |  |  |  |
| --- | --- | --- | --- | --- | --- | --- |
| 1 n (%) | | | | | | |
| 2 Fisher’s exact test; Kruskal-Wallis rank sum test | | | | | | |

## Tumor counts

### Model significance

Because the outcome is a count of tumor foci, we are using a poisson
regression model to look for differences in counts between genotypes. We
see that there is no significant difference overall in tumor counts
between the genotype groups.

```
model <- tumor_data %>%
  glm(total_foci ~ genotype, family = poisson, data = .)

print(car::Anova(model, type = 2))
```

```
## Analysis of Deviance Table (Type II tests)
## 
## Response: total_foci
##          LR Chisq Df Pr(>Chisq)  
## genotype   6.9674  3    0.07294 .
## ---
## Signif. codes:  0 '***' 0.001 '**' 0.01 '*' 0.05 '.' 0.1 ' ' 1
```

### Estimated marginal means

No significant pairwise differences in expected counts between
genotypes.

```
emmeans(model, pairwise ~ genotype, type = "response")
```

```
## $emmeans
##  genotype rate    SE  df asymp.LCL asymp.UCL
##  XXF      6.25 0.884 Inf      4.74      8.25
##  XXM      6.50 0.806 Inf      5.10      8.29
##  XYF      8.67 0.981 Inf      6.94     10.82
##  XYM      5.60 0.748 Inf      4.31      7.28
## 
## Confidence level used: 0.95 
## Intervals are back-transformed from the log scale 
## 
## $contrasts
##  contrast  ratio    SE  df null z.ratio p.value
##  XXF / XXM 0.962 0.181 Inf    1  -0.209  0.9968
##  XXF / XYF 0.721 0.131 Inf    1  -1.804  0.2711
##  XXF / XYM 1.116 0.217 Inf    1   0.564  0.9426
##  XXM / XYF 0.750 0.126 Inf    1  -1.713  0.3168
##  XXM / XYM 1.161 0.212 Inf    1   0.817  0.8463
##  XYF / XYM 1.548 0.271 Inf    1   2.493  0.0609
## 
## P value adjustment: tukey method for comparing a family of 4 estimates 
## Tests are performed on the log scale
```

```
# Compute emmeans with pairwise comparisons
emm <- emmeans(model, pairwise ~ genotype, type = "response")

# Extract the estimated marginal means (EMMs)
emm_df <- as.data.frame(emm$emmeans) 

# Extract the pairwise comparisons
pwc <- as.data.frame(emm$contrasts)

# Add y-position for p-value annotations
pwc <- pwc %>%
    mutate(y.position = max(emm_df$asymp.UCL) + 
               seq(0.1, 0.2, length.out = n())) %>%
    mutate(p.value = ifelse(p.value > 0.05, "", format.pval(p.value, eps = 0.001, digits = 1)))

# Create the plot
p <- ggplot(emm_df, aes(x = genotype, y = rate)) +
    geom_point(position = position_dodge(width = 0.1), size = 3) +
    geom_errorbar(aes(ymin = asymp.LCL, ymax = asymp.UCL), 
                  width = 0.2, position = position_dodge(width = 0.1)) +
    theme_bw() + # use a simple BW theme
    labs(title = paste("Effect plot of expected tumor counts, by genotype"), y = paste("Predicted value of tumor count"), x = "Genotype")

# format data for stat_pvalue
pwc <- pwc %>%
    mutate(
        treat1 = sub(" / .*", "", contrast),
        treat2 = sub(".* / ", "", contrast),
        group1 = treat1,
        group2 = treat2
    )
    # mutate(p.value = ifelse(p.value != "",
    #                         paste(group1, "vs.", group2, " p = ", p.value),
    #                         ""))


# Add p-values and display the plot
# final_plot <- p + stat_pvalue_manual(pwc, 
#                        x = "genotype",
#                        label = "p.value", 
#                        y.position = "y.position",
#                        tip.length = 0.01,
#                        step.increase = 0.5,
#                        label.size = 4)
# 
# 
# print(final_plot)

p
```

## Tumor area

Below we have standard linear models looking for differences in tumor
area by genotype. Sensitivity analyses removing the suspected outliers
do not change the results.

### Model significance

Because the outcome is now just a continuous measure of area, we will
use a standard linear model to model differences in area between
genotype groups.

```
model <- tumor_data %>%
  lm(tumor_area_mm2 ~ genotype, data = .)

print(car::Anova(model, type = 2))
```

```
## Anova Table (Type II tests)
## 
## Response: tumor_area_mm2
##            Sum Sq Df F value Pr(>F)
## genotype   194.15  3  0.7499 0.5302
## Residuals 2847.89 33
```

### Estimated marginal means

No significant pairwise differences in expected tumor area between
genotypes.

```
emmeans(model, pairwise ~ genotype)
```

```
## $emmeans
##  genotype emmean   SE df lower.CL upper.CL
##  XXF        2.49 3.28 33  -4.1949     9.17
##  XXM        7.22 2.94 33   1.2453    13.20
##  XYF        6.22 3.10 33  -0.0838    12.52
##  XYM        2.03 2.94 33  -3.9511     8.00
## 
## Confidence level used: 0.95 
## 
## $contrasts
##  contrast  estimate   SE df t.ratio p.value
##  XXF - XXM   -4.735 4.41 33  -1.074  0.7072
##  XXF - XYF   -3.729 4.51 33  -0.826  0.8417
##  XXF - XYM    0.462 4.41 33   0.105  0.9996
##  XXM - XYF    1.006 4.27 33   0.236  0.9953
##  XXM - XYM    5.196 4.15 33   1.251  0.5998
##  XYF - XYM    4.191 4.27 33   0.982  0.7607
## 
## P value adjustment: tukey method for comparing a family of 4 estimates
```

```
# Compute emmeans with pairwise comparisons
emm <- emmeans(model, pairwise ~ genotype)

# Extract the estimated marginal means (EMMs)
emm_df <- as.data.frame(emm$emmeans) 

# Extract the pairwise comparisons
pwc <- as.data.frame(emm$contrasts)

# Add y-position for p-value annotations
pwc <- pwc %>%
    mutate(y.position = max(emm_df$upper.CL) + 
               seq(0.1, 0.2, length.out = n())) %>%
    mutate(p.value = ifelse(p.value > 0.05, "", format.pval(p.value, eps = 0.001, digits = 1)))

# Create the plot
p <- ggplot(emm_df, aes(x = genotype, y = emmean)) +
    geom_point(position = position_dodge(width = 0.1), size = 3) +
    geom_errorbar(aes(ymin = lower.CL, ymax = upper.CL), 
                  width = 0.2, position = position_dodge(width = 0.1)) +
    theme_bw() + # use a simple BW theme
    labs(title = paste("Effect plot of expected tumor area, by genotype"), y = paste("Predicted value of tumor area"), x = "Genotype")

# format data for stat_pvalue
pwc <- pwc %>%
    mutate(
        treat1 = sub(" / .*", "", contrast),
        treat2 = sub(".* / ", "", contrast),
        group1 = treat1,
        group2 = treat2
    )
    # mutate(p.value = ifelse(p.value != "",
    #                         paste(group1, "vs.", group2, " p = ", p.value),
    #                         ""))


# Add p-values and display the plot
# final_plot <- p + stat_pvalue_manual(pwc, 
#                        x = "genotype",
#                        label = "p.value", 
#                        y.position = "y.position",
#                        tip.length = 0.01,
#                        step.increase = 0.5,
#                        label.size = 4)
# 
# 
# print(final_plot)

p
```

### Model significance (outliers 15 and 21 removed)

Removing the two suspected outliers (with large values) does not
change the significance of the model

```
model <- tumor_data[-c(15, 21),] %>%
  lm(tumor_area_mm2 ~ genotype, data = .)

print(car::Anova(model, type = 2))
```

```
## Anova Table (Type II tests)
## 
## Response: tumor_area_mm2
##            Sum Sq Df F value Pr(>F)
## genotype   14.257  3  1.1535 0.3432
## Residuals 127.718 31
```

### Estimated marginal means (outliers 15 and 21 removed)

No significant pairwise differences in expected tumor area between
genotypes.

```
emmeans(model, pairwise ~ genotype)
```

```
## $emmeans
##  genotype emmean    SE df lower.CL upper.CL
##  XXF        2.49 0.718 31    1.024     3.95
##  XXM        3.43 0.677 31    2.052     4.81
##  XYF        1.76 0.718 31    0.300     3.23
##  XYM        2.03 0.642 31    0.717     3.33
## 
## Confidence level used: 0.95 
## 
## $contrasts
##  contrast  estimate    SE df t.ratio p.value
##  XXF - XXM   -0.945 0.986 31  -0.958  0.7738
##  XXF - XYF    0.724 1.010 31   0.713  0.8911
##  XXF - XYM    0.462 0.963 31   0.480  0.9631
##  XXM - XYF    1.669 0.986 31   1.692  0.3449
##  XXM - XYM    1.407 0.933 31   1.508  0.4449
##  XYF - XYM   -0.262 0.963 31  -0.272  0.9928
## 
## P value adjustment: tukey method for comparing a family of 4 estimates
```

```
# Compute emmeans with pairwise comparisons
emm <- emmeans(model, pairwise ~ genotype)

# Extract the estimated marginal means (EMMs)
emm_df <- as.data.frame(emm$emmeans) 

# Extract the pairwise comparisons
pwc <- as.data.frame(emm$contrasts)

# Add y-position for p-value annotations
pwc <- pwc %>%
    mutate(y.position = max(emm_df$upper.CL) + 
               seq(0.1, 0.2, length.out = n())) %>%
    mutate(p.value = ifelse(p.value > 0.05, "", format.pval(p.value, eps = 0.001, digits = 1)))

# Create the plot
p <- ggplot(emm_df, aes(x = genotype, y = emmean)) +
    geom_point(position = position_dodge(width = 0.1), size = 3) +
    geom_errorbar(aes(ymin = lower.CL, ymax = upper.CL), 
                  width = 0.2, position = position_dodge(width = 0.1)) +
    theme_bw() + # use a simple BW theme
    labs(title = paste("Effect plot of expected tumor area, by genotype"), y = paste("Predicted value of tumor area"), x = "Genotype")

# format data for stat_pvalue
pwc <- pwc %>%
    mutate(
        treat1 = sub(" / .*", "", contrast),
        treat2 = sub(".* / ", "", contrast),
        group1 = treat1,
        group2 = treat2
    )
    # mutate(p.value = ifelse(p.value != "",
    #                         paste(group1, "vs.", group2, " p = ", p.value),
    #                         ""))


# Add p-values and display the plot
# final_plot <- p + stat_pvalue_manual(pwc, 
#                        x = "genotype",
#                        label = "p.value", 
#                        y.position = "y.position",
#                        tip.length = 0.01,
#                        step.increase = 0.5,
#                        label.size = 4)
# 
# 
# print(final_plot)

p
```

## Normalized tumor area

Below we have standard linear models looking for differences in
normalized tumor area (ratio of tumor area over lung area) by genotype.
Sensitivity analyses removing the suspected outliers do not change the
results.

### Model significance

When we model tumor area normalized to lung area (again using a
linear model) we again see no significant influence of genotype.

```
model <- tumor_data %>%
  lm(ratio ~ genotype, data = .)

print(car::Anova(model, type = 2))
```

```
## Anova Table (Type II tests)
## 
## Response: ratio
##              Sum Sq Df F value Pr(>F)
## genotype  0.0011698  3  0.7475 0.5316
## Residuals 0.0172129 33
```

### Estimated marginal means

No significant pairwise differences in expected tumor area between
genotypes.

```
emmeans(model, pairwise ~ genotype)
```

```
## $emmeans
##  genotype  emmean      SE df  lower.CL upper.CL
##  XXF      0.00537 0.00807 33 -0.011053   0.0218
##  XXM      0.01801 0.00722 33  0.003318   0.0327
##  XYF      0.01478 0.00761 33 -0.000707   0.0303
##  XYM      0.00555 0.00722 33 -0.009142   0.0202
## 
## Confidence level used: 0.95 
## 
## $contrasts
##  contrast   estimate     SE df t.ratio p.value
##  XXF - XXM -0.012637 0.0108 33  -1.167  0.6517
##  XXF - XYF -0.009407 0.0111 33  -0.848  0.8313
##  XXF - XYM -0.000177 0.0108 33  -0.016  1.0000
##  XXM - XYF  0.003230 0.0105 33   0.308  0.9897
##  XXM - XYM  0.012460 0.0102 33   1.220  0.6189
##  XYF - XYM  0.009230 0.0105 33   0.880  0.8154
## 
## P value adjustment: tukey method for comparing a family of 4 estimates
```

```
# Compute emmeans with pairwise comparisons
emm <- emmeans(model, pairwise ~ genotype)

# Extract the estimated marginal means (EMMs)
emm_df <- as.data.frame(emm$emmeans) 

# Extract the pairwise comparisons
pwc <- as.data.frame(emm$contrasts)

# Add y-position for p-value annotations
pwc <- pwc %>%
    mutate(y.position = max(emm_df$upper.CL) + 
               seq(0.1, 0.2, length.out = n())) %>%
    mutate(p.value = ifelse(p.value > 0.05, "", format.pval(p.value, eps = 0.001, digits = 1)))

# Create the plot
p <- ggplot(emm_df, aes(x = genotype, y = emmean)) +
    geom_point(position = position_dodge(width = 0.1), size = 3) +
    geom_errorbar(aes(ymin = lower.CL, ymax = upper.CL), 
                  width = 0.2, position = position_dodge(width = 0.1)) +
    theme_bw() + # use a simple BW theme
    labs(title = paste("Effect plot of expected tumor area, by genotype"), y = paste("Predicted value of tumor area"), x = "Genotype")

# format data for stat_pvalue
pwc <- pwc %>%
    mutate(
        treat1 = sub(" / .*", "", contrast),
        treat2 = sub(".* / ", "", contrast),
        group1 = treat1,
        group2 = treat2
    )
    # mutate(p.value = ifelse(p.value != "",
    #                         paste(group1, "vs.", group2, " p = ", p.value),
    #                         ""))


# Add p-values and display the plot
# final_plot <- p + stat_pvalue_manual(pwc, 
#                        x = "genotype",
#                        label = "p.value", 
#                        y.position = "y.position",
#                        tip.length = 0.01,
#                        step.increase = 0.5,
#                        label.size = 4)
# 
# 
# print(final_plot)

p
```

### Model significance (outliers 15 and 21 removed)

Removing the two suspected outliers (with large values) does not
change the significance of the model

```
model <- tumor_data[-c(15, 21),] %>%
  lm(ratio ~ genotype, data = .)

print(car::Anova(model, type = 2))
```

```
## Anova Table (Type II tests)
## 
## Response: ratio
##               Sum Sq Df F value Pr(>F)
## genotype  0.00011271  3  1.4723 0.2412
## Residuals 0.00079106 31
```

### Estimated marginal means (outliers 15 and 21 removed)

No significant pairwise differences in expected tumor area between
genotypes.

```
emmeans(model, pairwise ~ genotype)
```

```
## $emmeans
##  genotype  emmean      SE df lower.CL upper.CL
##  XXF      0.00537 0.00179 31 0.001732  0.00902
##  XXM      0.00876 0.00168 31 0.005322  0.01219
##  XYF      0.00378 0.00179 31 0.000139  0.00742
##  XYM      0.00555 0.00160 31 0.002294  0.00881
## 
## Confidence level used: 0.95 
## 
## $contrasts
##  contrast   estimate      SE df t.ratio p.value
##  XXF - XXM -0.003382 0.00245 31  -1.378  0.5224
##  XXF - XYF  0.001593 0.00253 31   0.631  0.9214
##  XXF - XYM -0.000177 0.00240 31  -0.074  0.9999
##  XXM - XYF  0.004975 0.00245 31   2.027  0.2003
##  XXM - XYM  0.003205 0.00232 31   1.381  0.5205
##  XYF - XYM -0.001770 0.00240 31  -0.739  0.8807
## 
## P value adjustment: tukey method for comparing a family of 4 estimates
```

```
# Compute emmeans with pairwise comparisons
emm <- emmeans(model, pairwise ~ genotype)

# Extract the estimated marginal means (EMMs)
emm_df <- as.data.frame(emm$emmeans) 

# Extract the pairwise comparisons
pwc <- as.data.frame(emm$contrasts)

# Add y-position for p-value annotations
pwc <- pwc %>%
    mutate(y.position = max(emm_df$upper.CL) + 
               seq(0.1, 0.2, length.out = n())) %>%
    mutate(p.value = ifelse(p.value > 0.05, "", format.pval(p.value, eps = 0.001, digits = 1)))

# Create the plot
p <- ggplot(emm_df, aes(x = genotype, y = emmean)) +
    geom_point(position = position_dodge(width = 0.1), size = 3) +
    geom_errorbar(aes(ymin = lower.CL, ymax = upper.CL), 
                  width = 0.2, position = position_dodge(width = 0.1)) +
    theme_bw() + # use a simple BW theme
    labs(title = paste("Effect plot of expected tumor area, by genotype"), y = paste("Predicted value of tumor area"), x = "Genotype")

# format data for stat_pvalue
pwc <- pwc %>%
    mutate(
        treat1 = sub(" / .*", "", contrast),
        treat2 = sub(".* / ", "", contrast),
        group1 = treat1,
        group2 = treat2
    )
    # mutate(p.value = ifelse(p.value != "",
    #                         paste(group1, "vs.", group2, " p = ", p.value),
    #                         ""))


# Add p-values and display the plot
# final_plot <- p + stat_pvalue_manual(pwc, 
#                        x = "genotype",
#                        label = "p.value", 
#                        y.position = "y.position",
#                        tip.length = 0.01,
#                        step.increase = 0.5,
#                        label.size = 4)
# 
# 
# print(final_plot)

p
```

## Ki-67 per tumor area (#Ki-67+/mm2)

Below we are using a standard linear model to look for differences in
normalized Ki-67 per tumor area based on genotypes.

### Model significance

We see no significant influence of genotype.

```
model <- tumor_data %>%
  lm(ki_67_cells_per_mm2 ~ genotype, data = .)

print(car::Anova(model, type = 2))
```

```
## Anova Table (Type II tests)
## 
## Response: ki_67_cells_per_mm2
##            Sum Sq Df F value Pr(>F)
## genotype   472446  3  0.5492 0.6551
## Residuals 5161782 18
```

### Estimated marginal means

No significant pairwise differences in expected normalized Ki-67
counts between genotypes.

```
emmeans(model, pairwise ~ genotype)
```

```
## $emmeans
##  genotype emmean  SE df lower.CL upper.CL
##  XXF         985 239 18      482     1488
##  XXM         748 219 18      289     1207
##  XYF         718 219 18      258     1177
##  XYM         555 239 18       52     1058
## 
## Confidence level used: 0.95 
## 
## $contrasts
##  contrast  estimate  SE df t.ratio p.value
##  XXF - XXM    237.0 324 18   0.731  0.8834
##  XXF - XYF    267.4 324 18   0.825  0.8421
##  XXF - XYM    429.9 339 18   1.269  0.5930
##  XXM - XYF     30.4 309 18   0.098  0.9996
##  XXM - XYM    192.9 324 18   0.595  0.9323
##  XYF - XYM    162.5 324 18   0.501  0.9577
## 
## P value adjustment: tukey method for comparing a family of 4 estimates
```

# BALF data

## Descriptive tables

From the unadjusted tables comparing counts within and between
genotype and treatment groups, we see that there are few significant
differences. We’ll use linear models adjusted for total cell counts per
mL next to see if there are any differences after we adjust for the
volume of cells.

```
# first table comparing genotypes within treatments
balf_data %>%
  select(genotype:total_macrophages) %>%
  tbl_strata(strata = treatment, # stratify table by treatment
             ~ .x %>%
  tbl_summary(by = genotype, # group table by genotype group
              missing_text = "Missing", 
                type = c(all_dichotomous() ~ "categorical",
                         all_continuous() ~ "continuous2"),
              statistic = list(all_continuous() ~ c("{mean} ({sd})", "{median} ({p25}, {p75})", "{min}, {max}"))) %>%
  bold_labels() %>%
  add_overall(last = T) %>% # add overall column
  add_p(pvalue_fun = label_style_pvalue(digits = 3)), # add p-value 
  .header = "**{strata}**, N = {n}" # add n and strata name to labels
  )
```

| **Characteristic** | **PBS**, N = 28 | | | | | | **Urethane**, N = 37 | | | | | |
| --- | --- | --- | --- | --- | --- | --- | --- | --- | --- | --- | --- | --- |
|  | **XXF**  N = 8 | **XXM**  N = 5 | **XYF**  N = 6 | **XYM**  N = 9 | **Overall**  N = 28 | **p-value**1 | **XXF**  N = 10 | **XXM**  N = 9 | **XYF**  N = 11 | **XYM**  N = 7 | **Overall**  N = 37 | **p-value**1 |
| cells\_m\_l |  |  |  |  |  | 0.097 |  |  |  |  |  | 0.516 |
| Mean (SD) | 268,750 (102,081) | 414,400 (192,633) | 577,167 (310,119) | 407,444 (241,654) | 405,429 (235,409) |  | 631,500 (338,422) | 847,667 (449,120) | 813,273 (352,879) | 939,571 (597,477) | 796,405 (424,059) |  |
| Median (Q1, Q3) | 259,000 (188,500, 329,500) | 434,000 (220,000, 586,000) | 494,000 (356,000, 670,000) | 309,000 (267,000, 455,000) | 329,500 (238,000, 520,500) |  | 599,500 (343,000, 675,000) | 795,000 (534,000, 963,000) | 821,000 (455,000, 1,070,000) | 905,000 (487,000, 1,290,000) | 685,000 (487,000, 999,000) |  |
| Min, Max | 141,000, 455,000 | 215,000, 617,000 | 309,000, 1,140,000 | 188,000, 947,000 | 141,000, 1,140,000 |  | 246,000, 1,420,000 | 345,000, 1,670,000 | 371,000, 1,410,000 | 246,000, 2,010,000 | 246,000, 2,010,000 |  |
| lymphocyte |  |  |  |  |  | 0.001 |  |  |  |  |  | 0.381 |
| Mean (SD) | 9,906 (6,370) | 3,919 (2,493) | 17,858 (9,604) | 2,458 (2,258) | 8,147 (8,124) |  | 52,609 (37,976) | 51,814 (52,480) | 46,351 (24,853) | 32,970 (45,581) | 46,840 (39,285) |  |
| Median (Q1, Q3) | 9,958 (5,440, 13,990) | 4,340 (3,225, 5,860) | 14,348 (10,680, 23,520) | 2,275 (940, 3,035) | 5,750 (2,473, 10,748) |  | 42,698 (19,440, 81,340) | 31,800 (15,525, 81,855) | 30,960 (28,200, 69,550) | 9,050 (6,450, 44,000) | 30,960 (15,700, 75,690) |  |
| Min, Max | 0, 20,475 | 0, 6,170 | 10,050, 34,200 | 0, 6,925 | 0, 34,200 |  | 8,610, 114,330 | 5,340, 164,450 | 14,840, 83,850 | 3,690, 130,650 | 3,690, 164,450 |  |
| macrophages |  |  |  |  |  | 0.075 |  |  |  |  |  | 0.329 |
| Mean (SD) | 248,663 (91,331) | 403,117 (186,877) | 546,549 (297,604) | 395,937 (235,284) | 387,415 (227,029) |  | 539,164 (299,954) | 761,319 (413,278) | 729,872 (340,235) | 838,294 (497,583) | 706,491 (382,024) |  |
| Median (Q1, Q3) | 239,665 (182,026, 296,605) | 410,130 (218,900, 574,280) | 459,930 (345,320, 636,500) | 299,730 (253,455, 441,350) | 302,290 (224,010, 499,305) |  | 493,805 (284,690, 617,625) | 701,220 (525,990, 780,030) | 735,875 (354,305, 920,200) | 841,650 (462,650, 1,115,850) | 617,625 (458,990, 919,080) |  |
| Min, Max | 131,835, 420,875 | 210,700, 601,575 | 288,915, 1,088,700 | 183,300, 913,855 | 131,835, 1,088,700 |  | 225,090, 1,263,800 | 302,175, 1,578,150 | 327,600, 1,374,750 | 242,310, 1,708,500 | 225,090, 1,708,500 |  |
| dividing\_macrophages |  |  |  |  |  | 0.962 |  |  |  |  |  | 0.030 |
| Mean (SD) | 8,429 (6,970) | 6,532 (7,773) | 7,644 (7,858) | 8,895 (8,665) | 8,072 (7,481) |  | 30,424 (20,301) | 9,211 (10,508) | 32,217 (25,222) | 57,846 (57,763) | 30,985 (33,463) |  |
| Median (Q1, Q3) | 5,550 (3,399, 15,858) | 5,860 (1,100, 6,170) | 6,803 (0, 11,400) | 6,120 (3,760, 11,375) | 6,000 (2,681, 13,238) |  | 27,518 (21,630, 40,670) | 3,950 (2,670, 12,090) | 29,435 (7,740, 49,950) | 44,000 (14,610, 109,650) | 24,185 (7,740, 40,820) |  |
| Min, Max | 0, 17,820 | 0, 19,530 | 0, 20,860 | 0, 28,410 | 0, 28,410 |  | 5,145, 78,100 | 0, 28,600 | 0, 80,250 | 0, 160,800 | 0, 160,800 |  |
| pm\_ns |  |  |  |  |  | 0.308 |  |  |  |  |  | 0.827 |
| Mean (SD) | 1,752 (3,197) | 832 (1,343) | 5,116 (9,249) | 154 (462) | 1,795 (4,727) |  | 9,304 (20,702) | 25,322 (39,799) | 4,832 (8,530) | 10,461 (21,283) | 12,090 (24,847) |  |
| Median (Q1, Q3) | 0 (0, 2,458) | 0 (0, 1,075) | 773 (0, 5,700) | 0 (0, 0) | 0 (0, 1,465) |  | 3,258 (0, 5,145) | 0 (0, 44,330) | 0 (0, 4,105) | 2,435 (0, 10,050) | 2,435 (0, 5,145) |  |
| Min, Max | 0, 9,100 | 0, 3,085 | 0, 23,450 | 0, 1,385 | 0, 23,450 |  | 0, 67,875 | 0, 101,115 | 0, 23,975 | 0, 58,050 | 0, 101,115 |  |
| total\_macrophages |  |  |  |  |  | 0.078 |  |  |  |  |  | 0.377 |
| Mean (SD) | 257,092 (93,628) | 409,649 (189,906) | 554,193 (299,937) | 404,832 (240,916) | 395,487 (229,806) |  | 569,588 (317,386) | 770,530 (415,251) | 762,089 (345,414) | 896,140 (553,131) | 737,476 (401,023) |  |
| Median (Q1, Q3) | 251,260 (183,663, 313,053) | 429,660 (220,000, 580,140) | 473,300 (345,320, 636,500) | 307,455 (264,330, 452,725) | 313,690 (232,880, 515,423) |  | 518,643 (299,700, 648,000) | 713,310 (528,660, 780,030) | 765,310 (386,750, 1,000,450) | 895,950 (477,260, 1,225,500) | 648,000 (477,260, 969,030) |  |
| Min, Max | 135,360, 425,425 | 210,700, 607,745 | 296,640, 1,100,100 | 187,060, 942,265 | 135,360, 1,100,100 |  | 234,930, 1,341,900 | 306,125, 1,578,150 | 352,170, 1,381,800 | 242,310, 1,869,300 | 234,930, 1,869,300 |  |
|  |  |  |  |  |  |  |  |  |  |  |  |  |
| --- | --- | --- | --- | --- | --- | --- | --- | --- | --- | --- | --- | --- |
| 1 Kruskal-Wallis rank sum test | | | | | | | | | | | | |

```
# second table comparing treatments within genotypes
balf_data %>%
  select(genotype:total_macrophages) %>%
  tbl_strata(strata = genotype, # stratify table by genotype
             ~ .x %>%
  tbl_summary(by = treatment, # group table by treatment group
              missing_text = "Missing", 
                type = c(all_dichotomous() ~ "categorical",
                         all_continuous() ~ "continuous2"),
              statistic = list(all_continuous() ~ c("{mean} ({sd})", "{median} ({p25}, {p75})", "{min}, {max}"))) %>%
  bold_labels() %>%
  add_overall(last = T) %>% # add overall column
  add_p(pvalue_fun = label_style_pvalue(digits = 3)), # add p-value 
  .header = "**{strata}**, N = {n}" # add n and strata name to labels
  )
```

| **Characteristic** | **XXF**, N = 18 | | | | **XXM**, N = 14 | | | | **XYF**, N = 17 | | | | **XYM**, N = 16 | | | |
| --- | --- | --- | --- | --- | --- | --- | --- | --- | --- | --- | --- | --- | --- | --- | --- | --- |
|  | **PBS**  N = 8 | **Urethane**  N = 10 | **Overall**  N = 18 | **p-value**1 | **PBS**  N = 52 | **Urethane**  N = 92 | **Overall**  N = 142 | **p-value**3 | **PBS**  N = 62 | **Urethane**  N = 112 | **Overall**  N = 172 | **p-value**3 | **PBS**  N = 92 | **Urethane**  N = 72 | **Overall**  N = 162 | **p-value**3 |
| cells\_m\_l |  |  |  | 0.006 |  |  |  | 0.060 |  |  |  | 0.149 |  |  |  | 0.042 |
| Mean (SD) | 268,750 (102,081) | 631,500 (338,422) | 470,278 (315,160) |  | 414,400 (192,633) | 847,667 (449,120) | 692,929 (426,568) |  | 577,167 (310,119) | 813,273 (352,879) | 729,941 (348,437) |  | 407,444 (241,654) | 939,571 (597,477) | 640,250 (498,262) |  |
| Median (Q1, Q3) | 259,000 (188,500, 329,500) | 599,500 (343,000, 675,000) | 339,000 (256,000, 618,000) |  | 434,000 (220,000, 586,000) | 795,000 (534,000, 963,000) | 601,500 (395,000, 806,000) |  | 494,000 (356,000, 670,000) | 821,000 (455,000, 1,070,000) | 670,000 (392,000, 999,000) |  | 309,000 (267,000, 455,000) | 905,000 (487,000, 1,290,000) | 471,000 (272,000, 926,000) |  |
| Min, Max | 141,000, 455,000 | 246,000, 1,420,000 | 141,000, 1,420,000 |  | 215,000, 617,000 | 345,000, 1,670,000 | 215,000, 1,670,000 |  | 309,000, 1,140,000 | 371,000, 1,410,000 | 309,000, 1,410,000 |  | 188,000, 947,000 | 246,000, 2,010,000 | 188,000, 2,010,000 |  |
| lymphocyte |  |  |  | 0.002 |  |  |  | 0.004 |  |  |  | 0.015 |  |  |  | 0.003 |
| Mean (SD) | 9,906 (6,370) | 52,609 (37,976) | 33,630 (35,454) |  | 3,919 (2,493) | 51,814 (52,480) | 34,709 (47,581) |  | 17,858 (9,604) | 46,351 (24,853) | 36,295 (24,736) |  | 2,458 (2,258) | 32,970 (45,581) | 15,807 (32,835) |  |
| Median (Q1, Q3) | 9,958 (5,440, 13,990) | 42,698 (19,440, 81,340) | 17,570 (9,675, 48,020) |  | 4,340 (3,225, 5,860) | 31,800 (15,525, 81,855) | 12,700 (5,340, 48,360) |  | 14,348 (10,680, 23,520) | 30,960 (28,200, 69,550) | 28,735 (17,880, 58,225) |  | 2,275 (940, 3,035) | 9,050 (6,450, 44,000) | 4,213 (1,910, 8,178) |  |
| Min, Max | 0, 20,475 | 8,610, 114,330 | 0, 114,330 |  | 0, 6,170 | 5,340, 164,450 | 0, 164,450 |  | 10,050, 34,200 | 14,840, 83,850 | 10,050, 83,850 |  | 0, 6,925 | 3,690, 130,650 | 0, 130,650 |  |
| macrophages |  |  |  | 0.009 |  |  |  | 0.060 |  |  |  | 0.301 |  |  |  | 0.042 |
| Mean (SD) | 248,663 (91,331) | 539,164 (299,954) | 410,052 (270,426) |  | 403,117 (186,877) | 761,319 (413,278) | 633,390 (384,158) |  | 546,549 (297,604) | 729,872 (340,235) | 665,170 (328,910) |  | 395,937 (235,284) | 838,294 (497,583) | 589,468 (424,177) |  |
| Median (Q1, Q3) | 239,665 (182,026, 296,605) | 493,805 (284,690, 617,625) | 296,605 (229,120, 511,750) |  | 410,130 (218,900, 574,280) | 701,220 (525,990, 780,030) | 587,928 (326,025, 755,250) |  | 459,930 (345,320, 636,500) | 735,875 (354,305, 920,200) | 586,150 (354,305, 919,080) |  | 299,730 (253,455, 441,350) | 841,650 (462,650, 1,115,850) | 452,000 (256,223, 877,753) |  |
| Min, Max | 131,835, 420,875 | 225,090, 1,263,800 | 131,835, 1,263,800 |  | 210,700, 601,575 | 302,175, 1,578,150 | 210,700, 1,578,150 |  | 288,915, 1,088,700 | 327,600, 1,374,750 | 288,915, 1,374,750 |  | 183,300, 913,855 | 242,310, 1,708,500 | 183,300, 1,708,500 |  |
| dividing\_macrophages |  |  |  | 0.002 |  |  |  | 0.789 |  |  |  | 0.039 |  |  |  | 0.039 |
| Mean (SD) | 8,429 (6,970) | 30,424 (20,301) | 20,648 (19,096) |  | 6,532 (7,773) | 9,211 (10,508) | 8,254 (9,398) |  | 7,644 (7,858) | 32,217 (25,222) | 23,544 (23,736) |  | 8,895 (8,665) | 57,846 (57,763) | 30,311 (44,763) |  |
| Median (Q1, Q3) | 5,550 (3,399, 15,858) | 27,518 (21,630, 40,670) | 17,230 (5,145, 29,160) |  | 5,860 (1,100, 6,170) | 3,950 (2,670, 12,090) | 4,905 (1,100, 12,090) |  | 6,803 (0, 11,400) | 29,435 (7,740, 49,950) | 11,400 (7,050, 37,675) |  | 6,120 (3,760, 11,375) | 44,000 (14,610, 109,650) | 12,993 (4,550, 36,205) |  |
| Min, Max | 0, 17,820 | 5,145, 78,100 | 0, 78,100 |  | 0, 19,530 | 0, 28,600 | 0, 28,600 |  | 0, 20,860 | 0, 80,250 | 0, 80,250 |  | 0, 28,410 | 0, 160,800 | 0, 160,800 |  |
| pm\_ns |  |  |  | 0.163 |  |  |  | 0.790 |  |  |  | 0.633 |  |  |  | 0.077 |
| Mean (SD) | 1,752 (3,197) | 9,304 (20,702) | 5,947 (15,685) |  |  |  |  |  |  |  |  |  |  |  |  |  |
| Median (Q1, Q3) | 0 (0, 2,458) | 3,258 (0, 5,145) | 2,068 (0, 4,860) |  |  |  |  |  |  |  |  |  |  |  |  |  |
| Min, Max | 0, 9,100 | 0, 67,875 | 0, 67,875 |  |  |  |  |  |  |  |  |  |  |  |  |  |
| total\_macrophages |  |  |  | 0.009 |  |  |  | 0.060 |  |  |  | 0.180 |  |  |  | 0.042 |
| Mean (SD) | 257,092 (93,628) | 569,588 (317,386) | 430,701 (287,175) |  | 409,649 (189,906) | 770,530 (415,251) | 641,644 (386,537) |  | 554,193 (299,937) | 762,089 (345,414) | 688,714 (336,407) |  | 404,832 (240,916) | 896,140 (553,131) | 619,779 (465,510) |  |
| Median (Q1, Q3) | 251,260 (183,663, 313,053) | 518,643 (299,700, 648,000) | 313,053 (245,760, 537,625) |  | 429,660 (220,000, 580,140) | 713,310 (528,660, 780,030) | 593,943 (329,475, 763,200) |  | 473,300 (345,320, 636,500) | 765,310 (386,750, 1,000,450) | 602,800 (368,480, 969,030) |  | 307,455 (264,330, 452,725) | 895,950 (477,260, 1,225,500) | 464,993 (266,510, 919,108) |  |
| Min, Max | 135,360, 425,425 | 234,930, 1,341,900 | 135,360, 1,341,900 |  | 210,700, 607,745 | 306,125, 1,578,150 | 210,700, 1,578,150 |  | 296,640, 1,100,100 | 352,170, 1,381,800 | 296,640, 1,381,800 |  | 187,060, 942,265 | 242,310, 1,869,300 | 187,060, 1,869,300 |  |
| 0 |  |  |  |  | 3 (60%) | 5 (56%) | 8 (57%) |  | 3 (50%) | 6 (55%) | 9 (53%) |  | 8 (89%) | 3 (43%) | 11 (69%) |  |
| 1075 |  |  |  |  | 1 (20%) | 0 (0%) | 1 (7.1%) |  |  |  |  |  |  |  |  |  |
| 3085 |  |  |  |  | 1 (20%) | 0 (0%) | 1 (7.1%) |  |  |  |  |  |  |  |  |  |
| 3455 |  |  |  |  | 0 (0%) | 1 (11%) | 1 (7.1%) |  |  |  |  |  |  |  |  |  |
| 44330 |  |  |  |  | 0 (0%) | 1 (11%) | 1 (7.1%) |  |  |  |  |  |  |  |  |  |
| 79000 |  |  |  |  | 0 (0%) | 1 (11%) | 1 (7.1%) |  |  |  |  |  |  |  |  |  |
| 101115 |  |  |  |  | 0 (0%) | 1 (11%) | 1 (7.1%) |  |  |  |  |  |  |  |  |  |
| 1545 |  |  |  |  |  |  |  |  | 1 (17%) | 0 (0%) | 1 (5.9%) |  |  |  |  |  |
| 1855 |  |  |  |  |  |  |  |  | 0 (0%) | 1 (9.1%) | 1 (5.9%) |  |  |  |  |  |
| 3870 |  |  |  |  |  |  |  |  | 0 (0%) | 1 (9.1%) | 1 (5.9%) |  |  |  |  |  |
| 4105 |  |  |  |  |  |  |  |  | 0 (0%) | 1 (9.1%) | 1 (5.9%) |  |  |  |  |  |
| 5700 |  |  |  |  |  |  |  |  | 1 (17%) | 0 (0%) | 1 (5.9%) |  |  |  |  |  |
| 19350 |  |  |  |  |  |  |  |  | 0 (0%) | 1 (9.1%) | 1 (5.9%) |  |  |  |  |  |
| 23450 |  |  |  |  |  |  |  |  | 1 (17%) | 0 (0%) | 1 (5.9%) |  |  |  |  |  |
| 23975 |  |  |  |  |  |  |  |  | 0 (0%) | 1 (9.1%) | 1 (5.9%) |  |  |  |  |  |
| 1385 |  |  |  |  |  |  |  |  |  |  |  |  | 1 (11%) | 0 (0%) | 1 (6.3%) |  |
| 2435 |  |  |  |  |  |  |  |  |  |  |  |  | 0 (0%) | 1 (14%) | 1 (6.3%) |  |
| 2695 |  |  |  |  |  |  |  |  |  |  |  |  | 0 (0%) | 1 (14%) | 1 (6.3%) |  |
| 10050 |  |  |  |  |  |  |  |  |  |  |  |  | 0 (0%) | 1 (14%) | 1 (6.3%) |  |
| 58050 |  |  |  |  |  |  |  |  |  |  |  |  | 0 (0%) | 1 (14%) | 1 (6.3%) |  |
|  |  |  |  |  |  |  |  |  |  |  |  |  |  |  |  |  |
| --- | --- | --- | --- | --- | --- | --- | --- | --- | --- | --- | --- | --- | --- | --- | --- | --- |
| 1 Wilcoxon rank sum test; Wilcoxon rank sum exact test | | | | | | | | | | | | | | | | |
| 2 n (%) | | | | | | | | | | | | | | | | |
| 3 Wilcoxon rank sum exact test; Wilcoxon rank sum test; Fisher’s exact test | | | | | | | | | | | | | | | | |

## Descriptive plots

Mean and 95% confidence intervals of cell counts for genotype and
treatment groups.

### All raw values

```
# first plot of all values, by treatment colored by genotype
balf_data %>%
  select(genotype:total_macrophages) %>%
  pivot_longer(cols = cells_m_l:total_macrophages, names_to = "measure", values_to = "value") %>%
    ggplot(aes(x = treatment, y = value, color = genotype)) +
    stat_summary(fun = mean,
                 geom = "point",
                 size = 3,
                 position = position_dodge(width = 0.3)) +
    stat_summary(fun.data = mean_cl_normal,
                 geom = "errorbar",
                 width = 0.1,
                 position = position_dodge(width = 0.3)) +
    geom_point(alpha = 0.2, 
               position = position_dodge(width = 0.3)) +
    facet_wrap(~ measure, scales = "free") + # facet the plot by the measure
    theme_minimal() +
    labs(x = "Treatment", y = "Counts (or cell volume)", color = "Genotype")
```

```
# second plot of all values, by genotype colored by treatment
balf_data %>%
  select(genotype:total_macrophages) %>%
  pivot_longer(cols = cells_m_l:total_macrophages, names_to = "measure", values_to = "value") %>%
    ggplot(aes(x = genotype, y = value, color = treatment)) +
    stat_summary(fun = mean,
                 geom = "point",
                 size = 3,
                 position = position_dodge(width = 0.3)) +
    stat_summary(fun.data = mean_cl_normal,
                 geom = "errorbar",
                 width = 0.1,
                 position = position_dodge(width = 0.3)) +
    geom_point(alpha = 0.2, 
               position = position_dodge(width = 0.3)) +
    facet_wrap(~ measure, scales = "free") + # facet the plot by the measure
    theme_minimal() +
    labs(x = "Genotype", y = "Counts (or cell volume)", color = "Treatment")
```

### Relative values (count / cell volume) \* 100

```
# next set of plots where we calculate the counts per volume (%)
balf_data %>%
  mutate(across(lymphocyte:total_macrophages, ~ .x / cells_m_l *100)) %>% # divide all the cell counts by volume
  select(genotype:treatment, lymphocyte:total_macrophages) %>%
  pivot_longer(cols = lymphocyte:total_macrophages, names_to = "measure", values_to = "value") %>%
    ggplot(aes(x = treatment, y = value, color = genotype)) +
    stat_summary(fun = mean,
                 geom = "point",
                 size = 3,
                 position = position_dodge(width = 0.3)) +
    stat_summary(fun.data = mean_cl_normal,
                 geom = "errorbar",
                 width = 0.1,
                 position = position_dodge(width = 0.3)) +
    geom_point(alpha = 0.2, 
               position = position_dodge(width = 0.3)) +
    facet_wrap(~ measure, scales = "free") + # facet the plot by the measure
    theme_minimal() +
    labs(x = "Treatment", y = "Counts per cell volume (%)", color = "Genotype")
```

```
balf_data %>%
  mutate(across(lymphocyte:total_macrophages, ~ .x / cells_m_l *100)) %>% # divide all the cell counts by volume
  select(genotype:treatment, lymphocyte:total_macrophages) %>%
  pivot_longer(cols = lymphocyte:total_macrophages, names_to = "measure", values_to = "value") %>%
    ggplot(aes(x = genotype, y = value, color = treatment)) +
    stat_summary(fun = mean,
                 geom = "point",
                 size = 3,
                 position = position_dodge(width = 0.3)) +
    stat_summary(fun.data = mean_cl_normal,
                 geom = "errorbar",
                 width = 0.1,
                 position = position_dodge(width = 0.3)) +
    geom_point(alpha = 0.2, 
               position = position_dodge(width = 0.3)) +
    facet_wrap(~ measure, scales = "free") + # facet the plot by the measure
    theme_minimal() +
    labs(x = "Genotype", y = "Counts per cell volume (%)", color = "Treatment")
```

## Linear models

Below we are fitting linear models for counts of each cell type,
where each model is adjusted for total cell volume which essentially
adjusts the analysis for the fact that different samples have different
amounts of total possible cells. But, like before with the weight data,
this method is more statistically powerful and accurate than modeling
percentages.

In each model we are first looking for a treatment by genotype
interaction, and if one is not present we are dropping the interaction
and just looking at the significance of the main effects.

### Lymphocytes

#### Model significance (anova table)

No significant interaction between genotype and treatment, but when
we drop the interaction we see that the main effects of treatment and
genotype are both significant.

```
model <- balf_data %>%
  lm(lymphocyte ~ genotype * treatment + cells_m_l, data = .)

print(car::Anova(model))
```

```
## Anova Table (Type II tests)
## 
## Response: lymphocyte
##                        Sum Sq Df F value    Pr(>F)    
## genotype           5.0195e+09  3  2.8599   0.04494 *  
## treatment          3.0765e+09  1  5.2586   0.02561 *  
## cells_m_l          2.1704e+10  1 37.0977 1.081e-07 ***
## genotype:treatment 1.2594e+09  3  0.7175   0.54567    
## Residuals          3.2762e+10 56                      
## ---
## Signif. codes:  0 '***' 0.001 '**' 0.01 '*' 0.05 '.' 0.1 ' ' 1
```

```
model <- balf_data %>%
  lm(lymphocyte ~ genotype + treatment + cells_m_l, data = .)


model %>%
  tbl_regression(pvalue_fun = ~ style_pvalue(.x, digits = 3)) %>%
  add_global_p() %>% # add type 3 pvalue
  bold_p(t = 0.05) %>% # bold type 3 anova, global, p-values below 0.05.
  bold_labels()
```

| **Characteristic** | **Beta** | **95% CI** | **p-value** |
| --- | --- | --- | --- |
| genotype |  |  | 0.042 |
| XXF | — | — |  |
| XXM | -12,326 | -29,804, 5,151 |  |
| XYF | -12,802 | -29,566, 3,962 |  |
| XYM | -25,068 | -42,075, -8,062 |  |
| treatment |  |  | 0.024 |
| PBS | — | — |  |
| Urethane | 16,178 | 2,163, 30,193 |  |
| cells\_m\_l | 0.05 | 0.04, 0.07 | <0.001 |
|  |  |  |  |
| --- | --- | --- | --- |
| Abbreviation: CI = Confidence Interval | | | |

#### Estimated marginal means

See significant differences in expected counts between XXF and XYM
genotypes, and between PBS and Urethane groups.

```
summary(emmeans(model, pairwise ~ genotype))
```

```
## $emmeans
##  genotype emmean   SE df lower.CL upper.CL
##  XXF       41226 5890 59    29445    53007
##  XXM       28900 6480 59    15939    41861
##  XYF       28424 5910 59    16606    40242
##  XYM       16158 6020 59     4103    28212
## 
## Results are averaged over the levels of: treatment 
## Confidence level used: 0.95 
## 
## $contrasts
##  contrast  estimate   SE df t.ratio p.value
##  XXF - XXM    12326 8730 59   1.411  0.4975
##  XXF - XYF    12802 8380 59   1.528  0.4275
##  XXF - XYM    25068 8500 59   2.950  0.0230
##  XXM - XYF      476 8670 59   0.055  0.9999
##  XXM - XYM    12742 8880 59   1.435  0.4831
##  XYF - XYM    12266 8460 59   1.450  0.4741
## 
## Results are averaged over the levels of: treatment 
## P value adjustment: tukey method for comparing a family of 4 estimates
```

```
summary(emmeans(model, pairwise ~ treatment))
```

```
## $emmeans
##  treatment emmean   SE df lower.CL upper.CL
##  PBS        20588 4970 59    10634    30542
##  Urethane   36766 4260 59    28246    45286
## 
## Results are averaged over the levels of: genotype 
## Confidence level used: 0.95 
## 
## $contrasts
##  contrast       estimate   SE df t.ratio p.value
##  PBS - Urethane   -16178 7000 59  -2.310  0.0244
## 
## Results are averaged over the levels of: genotype
```

### Macrophages

#### Model significance (anova table)

No significant interaction between genotype and treatment, but when
we drop the interaction we see that the main effect of treatment is
significant.

```
model <- balf_data %>%
  lm(macrophages ~ genotype * treatment + cells_m_l, data = .)

print(car::Anova(model))
```

```
## Anova Table (Type II tests)
## 
## Response: macrophages
##                        Sum Sq Df   F value    Pr(>F)    
## genotype           7.3450e+09  3    1.6647  0.184999    
## treatment          1.2790e+10  1    8.6960  0.004646 ** 
## cells_m_l          5.8207e+12  1 3957.6585 < 2.2e-16 ***
## genotype:treatment 1.3810e+08  3    0.0313  0.992472    
## Residuals          8.2362e+10 56                        
## ---
## Signif. codes:  0 '***' 0.001 '**' 0.01 '*' 0.05 '.' 0.1 ' ' 1
```

```
model <- balf_data %>%
  lm(macrophages ~ genotype + treatment + cells_m_l, data = .)


model %>%
  tbl_regression(pvalue_fun = ~ style_pvalue(.x, digits = 3)) %>%
  add_global_p() %>% # add type 3 pvalue
  bold_p(t = 0.05) %>% # bold type 3 anova, global, p-values below 0.05.
  bold_labels()
```

| **Characteristic** | **Beta** | **95% CI** | **p-value** |
| --- | --- | --- | --- |
| genotype |  |  | 0.166 |
| XXF | — | — |  |
| XXM | 26,147 | -1,069, 53,364 |  |
| XYF | 24,807 | -1,299, 50,912 |  |
| XYM | 22,788 | -3,695, 49,271 |  |
| treatment |  |  | 0.004 |
| PBS | — | — |  |
| Urethane | -32,986 | -54,811, -11,161 |  |
| cells\_m\_l | 0.90 | 0.87, 0.93 | <0.001 |
|  |  |  |  |
| --- | --- | --- | --- |
| Abbreviation: CI = Confidence Interval | | | |

#### Estimated marginal means

See significant differences in expected counts between PBS and
Urethane groups.

```
summary(emmeans(model, pairwise ~ treatment))
```

```
## $emmeans
##  treatment emmean   SE df lower.CL upper.CL
##  PBS       588526 7750 59   573026   604026
##  Urethane  555540 6630 59   542272   568808
## 
## Results are averaged over the levels of: genotype 
## Confidence level used: 0.95 
## 
## $contrasts
##  contrast       estimate    SE df t.ratio p.value
##  PBS - Urethane    32986 10900 59   3.024  0.0037
## 
## Results are averaged over the levels of: genotype
```

### Dividing Macrophages

#### Model significance (anova table)

There is a significant interaction between genotype and treatment
here.

```
model <- balf_data %>%
  lm(dividing_macrophages ~ genotype * treatment + cells_m_l, data = .)

print(car::Anova(model, type = 3))
```

```
## Anova Table (Type III tests)
## 
## Response: dividing_macrophages
##                        Sum Sq Df F value    Pr(>F)    
## (Intercept)        6.2122e+07  1  0.1768   0.67575    
## genotype           6.4659e+08  3  0.6134   0.60917    
## treatment          1.8498e+08  1  0.5264   0.47113    
## cells_m_l          1.2788e+10  1 36.3932 1.345e-07 ***
## genotype:treatment 3.2959e+09  3  3.1266   0.03283 *  
## Residuals          1.9677e+10 56                      
## ---
## Signif. codes:  0 '***' 0.001 '**' 0.01 '*' 0.05 '.' 0.1 ' ' 1
```

```
model %>%
  tbl_regression(pvalue_fun = ~ style_pvalue(.x, digits = 3)) %>%
  add_global_p() %>% # add type 3 pvalue
  bold_p(t = 0.05) %>% # bold type 3 anova, global, p-values below 0.05.
  bold_labels()
```

| **Characteristic** | **Beta** | **95% CI** | **p-value** |
| --- | --- | --- | --- |
| genotype |  |  | 0.609 |
| XXF | — | — |  |
| XXM | -8,035 | -29,539, 13,469 |  |
| XYF | -13,782 | -34,516, 6,952 |  |
| XYM | -5,379 | -23,728, 12,971 |  |
| treatment |  |  | 0.471 |
| PBS | — | — |  |
| Urethane | 6,708 | -11,813, 25,229 |  |
| cells\_m\_l | 0.04 | 0.03, 0.06 | <0.001 |
| genotype \* treatment |  |  | 0.033 |
| XXM \* Urethane | -22,288 | -49,800, 5,224 |  |
| XYF \* Urethane | 7,915 | -18,231, 34,061 |  |
| XYM \* Urethane | 19,818 | -6,278, 45,914 |  |
|  |  |  |  |
| --- | --- | --- | --- |
| Abbreviation: CI = Confidence Interval | | | |

#### Estimated marginal means

See a few significant differences between treatments or genotypes
within the other grouping. XYM animals have have significantly higher
counts when treated with urethane instead of PBS. Also, see significant
differences in counts between XXF/XXM, XXM/XYF, and XXM/XYM animals
treated with urethane.

```
summary(emmeans(model, pairwise ~ genotype | treatment))
```

```
## $emmeans
## treatment = PBS:
##  genotype emmean   SE df lower.CL upper.CL
##  XXF       23568 7090 56     9371    37764
##  XXM       15533 8510 56    -1525    32590
##  XYF        9786 7660 56    -5561    25132
##  XYM       18189 6440 56     5297    31081
## 
## treatment = Urethane:
##  genotype emmean   SE df lower.CL upper.CL
##  XXF       30276 5930 56    18401    42151
##  XXM         -47 6430 56   -12936    12842
##  XYF       24409 5800 56    12794    36024
##  XYM       44715 7410 56    29868    59563
## 
## Confidence level used: 0.95 
## 
## $contrasts
## treatment = PBS:
##  contrast  estimate    SE df t.ratio p.value
##  XXF - XXM     8035 10700 56   0.749  0.8769
##  XXF - XYF    13782 10400 56   1.332  0.5471
##  XXF - XYM     5379  9160 56   0.587  0.9355
##  XXM - XYF     5747 11400 56   0.504  0.9578
##  XXM - XYM    -2656 10500 56  -0.254  0.9942
##  XYF - XYM    -8403  9950 56  -0.844  0.8330
## 
## treatment = Urethane:
##  contrast  estimate    SE df t.ratio p.value
##  XXF - XXM    30323  8740 56   3.468  0.0055
##  XXF - XYF     5867  8290 56   0.708  0.8936
##  XXF - XYM   -14439  9490 56  -1.522  0.4312
##  XXM - XYF   -24456  8430 56  -2.902  0.0265
##  XXM - XYM   -44762  9470 56  -4.728  0.0001
##  XYF - XYM   -20306  9110 56  -2.230  0.1277
## 
## P value adjustment: tukey method for comparing a family of 4 estimates
```

```
summary(emmeans(model, pairwise ~ treatment | genotype))
```

```
## $emmeans
## genotype = XXF:
##  treatment emmean   SE df lower.CL upper.CL
##  PBS        23568 7090 56     9371    37764
##  Urethane   30276 5930 56    18401    42151
## 
## genotype = XXM:
##  treatment emmean   SE df lower.CL upper.CL
##  PBS        15533 8510 56    -1525    32590
##  Urethane     -47 6430 56   -12936    12842
## 
## genotype = XYF:
##  treatment emmean   SE df lower.CL upper.CL
##  PBS         9786 7660 56    -5561    25132
##  Urethane   24409 5800 56    12794    36024
## 
## genotype = XYM:
##  treatment emmean   SE df lower.CL upper.CL
##  PBS        18189 6440 56     5297    31081
##  Urethane   44715 7410 56    29868    59563
## 
## Confidence level used: 0.95 
## 
## $contrasts
## genotype = XXF:
##  contrast       estimate    SE df t.ratio p.value
##  PBS - Urethane    -6708  9250 56  -0.726  0.4711
## 
## genotype = XXM:
##  contrast       estimate    SE df t.ratio p.value
##  PBS - Urethane    15580 10900 56   1.431  0.1579
## 
## genotype = XYF:
##  contrast       estimate    SE df t.ratio p.value
##  PBS - Urethane   -14623  9660 56  -1.515  0.1355
## 
## genotype = XYM:
##  contrast       estimate    SE df t.ratio p.value
##  PBS - Urethane   -26526 10200 56  -2.613  0.0115
```

### PMNs

#### Model significance (anova table)

No significant interaction or main effects.

```
model <- balf_data %>%
  lm(pm_ns ~ genotype * treatment + cells_m_l, data = .)

print(car::Anova(model, type = 2))
```

```
## Anova Table (Type II tests)
## 
## Response: pm_ns
##                        Sum Sq Df F value Pr(>F)
## genotype           1.2244e+09  3  1.1218 0.3481
## treatment          7.2903e+08  1  2.0039 0.1624
## cells_m_l          1.0901e+08  1  0.2996 0.5863
## genotype:treatment 1.0390e+09  3  0.9520 0.4218
## Residuals          2.0373e+10 56
```

```
model <- balf_data %>%
  lm(pm_ns ~ genotype + treatment + cells_m_l, data = .)

print(car::Anova(model, type = 2))
```

```
## Anova Table (Type II tests)
## 
## Response: pm_ns
##               Sum Sq Df F value Pr(>F)
## genotype  1.2244e+09  3  1.1245 0.3465
## treatment 7.2903e+08  1  2.0088 0.1616
## cells_m_l 1.8188e+08  1  0.5012 0.4818
## Residuals 2.1412e+10 59
```

### Total Macrophages

#### Model significance (anova table)

No significant interaction, but the main effect for treatment is
significant.

```
model <- balf_data %>%
  lm(total_macrophages ~ genotype * treatment + cells_m_l, data = .)

print(car::Anova(model, type = 2))
```

```
## Anova Table (Type II tests)
## 
## Response: total_macrophages
##                        Sum Sq Df   F value  Pr(>F)    
## genotype           6.7377e+09  3    2.2652 0.09085 .  
## treatment          6.8008e+09  1    6.8592 0.01133 *  
## cells_m_l          6.3791e+12  1 6433.9007 < 2e-16 ***
## genotype:treatment 2.9824e+09  3    1.0027 0.39848    
## Residuals          5.5523e+10 56                      
## ---
## Signif. codes:  0 '***' 0.001 '**' 0.01 '*' 0.05 '.' 0.1 ' ' 1
```

```
model <- balf_data %>%
  lm(total_macrophages ~ genotype + treatment + cells_m_l, data = .)

model %>%
  tbl_regression(pvalue_fun = ~ style_pvalue(.x, digits = 3)) %>%
  add_global_p() %>% # add type 3 pvalue
  bold_p(t = 0.05) %>% # bold type 3 anova, global, p-values below 0.05.
  bold_labels()
```

| **Characteristic** | **Beta** | **95% CI** | **p-value** |
| --- | --- | --- | --- |
| genotype |  |  | 0.090 |
| XXF | — | — |  |
| XXM | 3,491 | -19,429, 26,410 |  |
| XYF | 15,827 | -6,157, 37,811 |  |
| XYM | 26,266 | 3,964, 48,568 |  |
| treatment |  |  | 0.011 |
| PBS | — | — |  |
| Urethane | -24,054 | -42,433, -5,675 |  |
| cells\_m\_l | 0.94 | 0.92, 0.96 | <0.001 |
|  |  |  |  |
| --- | --- | --- | --- |
| Abbreviation: CI = Confidence Interval | | | |

#### Estimated marginal means

Like in the model table, we see that animals treated with Urethane
have significantly lower total macrophage counts as compared to animals
treated with PBS.

```
summary(emmeans(model, pairwise ~ treatment))
```

```
## $emmeans
##  treatment emmean   SE df lower.CL upper.CL
##  PBS       603889 6520 59   590836   616942
##  Urethane  579835 5580 59   568662   591008
## 
## Results are averaged over the levels of: genotype 
## Confidence level used: 0.95 
## 
## $contrasts
##  contrast       estimate   SE df t.ratio p.value
##  PBS - Urethane    24054 9180 59   2.619  0.0112
## 
## Results are averaged over the levels of: genotype
```

# PCR data

Here we’re just going to look at the 2^-ddCt values descriptively and
run an ANOVA model on the dCt values as they are generally a bit better
suited to analysis because the data are distributed better.

## Descriptive plots of relative fold change

Mean and 95% confidence intervals of cell counts for genotype and
treatment groups.

```
# first plot of all values, by treatment colored by genotype
pcr_data %>%
  select(genotype, treatment, x2_dd_ct) %>%
    ggplot(aes(x = treatment, y = x2_dd_ct, color = genotype)) +
    stat_summary(fun = mean,
                 geom = "point",
                 size = 3,
                 position = position_dodge(width = 0.3)) +
    stat_summary(fun.data = mean_cl_normal,
                 geom = "errorbar",
                 width = 0.1,
                 position = position_dodge(width = 0.3)) +
    geom_point(alpha = 0.2, 
               position = position_dodge(width = 0.3)) +
    theme_minimal() +
    labs(x = "Treatment", y = "Relative fold change (2^-ddCt)", color = "Genotype")
```

```
pcr_data %>%
  select(genotype, treatment, x2_dd_ct) %>%
    ggplot(aes(x = genotype, y = x2_dd_ct, color = treatment)) +
    stat_summary(fun = mean,
                 geom = "point",
                 size = 3,
                 position = position_dodge(width = 0.3)) +
    stat_summary(fun.data = mean_cl_normal,
                 geom = "errorbar",
                 width = 0.1,
                 position = position_dodge(width = 0.3)) +
    geom_point(alpha = 0.2, 
               position = position_dodge(width = 0.3)) +
    theme_minimal() +
    labs(x = "Genotype", y = "Relative fold change (2^-ddCt)", color = "Treatment")
```

## Linear models of dCt

Below we are fitting a linear model using dCt as the outcome. Based
on prior experience, literature, and some checks with these data I know
that using ANOVA type models that assume normality of residuals on
2^-ddCt/fold change values does not work well. dCt values are better
suited for these types of models, so that’s what we’re using here (and
residual plots show that the dCt values behave very well in this case
too).

### Model significance (anova table)

No significant interactions or main effects in these data.

```
model <- pcr_data %>%
  lm(d_ct ~ genotype * treatment, data = .)

print(car::Anova(model))
```

```
## Anova Table (Type II tests)
## 
## Response: d_ct
##                     Sum Sq Df F value  Pr(>F)  
## genotype            4.5108  3  2.4793 0.07627 .
## treatment           0.3622  1  0.5972 0.44454  
## genotype:treatment  2.9305  3  1.6107 0.20339  
## Residuals          22.4391 37                  
## ---
## Signif. codes:  0 '***' 0.001 '**' 0.01 '*' 0.05 '.' 0.1 ' ' 1
```

```
model <- pcr_data %>%
  lm(d_ct ~ genotype + treatment, data = .)

print(car::Anova(model))
```

```
## Anova Table (Type II tests)
## 
## Response: d_ct
##            Sum Sq Df F value  Pr(>F)  
## genotype   4.5108  3  2.3707 0.08483 .
## treatment  0.3622  1  0.5711 0.45426  
## Residuals 25.3696 40                  
## ---
## Signif. codes:  0 '***' 0.001 '**' 0.01 '*' 0.05 '.' 0.1 ' ' 1
```

# Survival data

Below we are going to look at the survival data, to see if there are
any relationships between survival rates (of the 16 animals who died
before protocol end) and 1) lymphoma, and 2) treatment (PBS
vs. Urethane).

Keep in mind that the number of animals dying early (16 / 162) and
those with confirmed lymphoma (5 / 162) are **very** small
- so these analyses should be interpreted *very* cautiously.

For these analyses I am using both Cox models and Kaplan-Meier curves
with Log-rank tests to look for differences in survival times between
the groups. Note that in this case where we have a simple analysis (one
variable predicting the differences in survival times) the Cox model and
log-rank test are essentially equivalent.

## Survival vs. lymphoma status (confirmed lymphoma cases)

Below we see that both the Cox model and log-rank test are
significant, indicating that animals with confirmed lymphoma cases have
significantly reduced survival time as compared to those who did not.
Again though, the sample sizes are so different between the groups that
these tests should not be given too much weight and must be discussed
cautiously.

```
cox <- coxph(Surv(protocol_week, status) ~ lymphoma, data = survival_data)

survfit <- survfit(Surv(protocol_week, status) ~ lymphoma, data = survival_data)

summary(cox)
```

```
## Call:
## coxph(formula = Surv(protocol_week, status) ~ lymphoma, data = survival_data)
## 
##   n= 162, number of events= 16 
## 
##             coef exp(coef) se(coef)     z Pr(>|z|)    
## lymphoma  3.3711   29.1101   0.5687 5.928 3.07e-09 ***
## ---
## Signif. codes:  0 '***' 0.001 '**' 0.01 '*' 0.05 '.' 0.1 ' ' 1
## 
##          exp(coef) exp(-coef) lower .95 upper .95
## lymphoma     29.11    0.03435      9.55     88.74
## 
## Concordance= 0.648  (se = 0.055 )
## Likelihood ratio test= 21.56  on 1 df,   p=3e-06
## Wald test            = 35.14  on 1 df,   p=3e-09
## Score (logrank) test = 81.54  on 1 df,   p=<2e-16
```

```
# survfit %>%
#   ggsurvfit() +
#   add_risktable() + 
#   scale_ggsurvfit() +
#   annotate("text", x = 2, y = 0.05, label = glue::glue("{survfit2_p(survfit)}"))

ggsurvplot(fit = survfit,
                      pval = TRUE,
                      pval.method = TRUE,
                      risk.table = TRUE, 
                      conf.int = FALSE, 
                      xlab = "Time (Weeks)", 
                      palette = c("#E66100", "#5D3A9B"))
```

## Survival vs. lymphoma status (counting “found dead” as lymphoma cases)

Again, when we are more liberal and count the 5 animals found dead as
lymphoma cases we see that there are still significant differences in
survival time between the animals with lymphoma and those without.

```
survival_data <- survival_data %>%
    mutate(lymphoma_all = ifelse(death_type == "Found dead" & !is.na(death_type) | lymphoma == 1, 1, 0)) # make new lymphoma variable that assumes animals found dead had lymphoma

cox <- coxph(Surv(protocol_week, status) ~ lymphoma_all, data = survival_data)

survfit <- survfit(Surv(protocol_week, status) ~ lymphoma_all, data = survival_data)

summary(cox)
```

```
## Call:
## coxph(formula = Surv(protocol_week, status) ~ lymphoma_all, data = survival_data)
## 
##   n= 162, number of events= 16 
## 
##                 coef exp(coef) se(coef)     z Pr(>|z|)    
## lymphoma_all  4.1596   64.0459   0.5509 7.551 4.33e-14 ***
## ---
## Signif. codes:  0 '***' 0.001 '**' 0.01 '*' 0.05 '.' 0.1 ' ' 1
## 
##              exp(coef) exp(-coef) lower .95 upper .95
## lymphoma_all     64.05    0.01561     21.76     188.5
## 
## Concordance= 0.799  (se = 0.058 )
## Likelihood ratio test= 49.85  on 1 df,   p=2e-12
## Wald test            = 57.01  on 1 df,   p=4e-14
## Score (logrank) test = 178.6  on 1 df,   p=<2e-16
```

```
# survfit %>%
#   ggsurvfit() +
#   add_risktable() + 
#   scale_ggsurvfit() +
#   annotate("text", x = 2, y = 0.05, label = glue::glue("{survfit2_p(survfit)}"))

ggsurvplot(fit = survfit,
                      pval = TRUE,
                      pval.method = TRUE,
                      risk.table = TRUE, 
                      conf.int = FALSE, 
                      xlab = "Time (Weeks)", 
                      palette = c("#E66100", "#5D3A9B"))
```

## Survival vs. treatment group

Close to a significant difference in survival between animals treated
with PBS and Urethane. Far more animals treated with Urethane (14) died
as compared to animals treated with PBS (2), but the difference is not
quite statistically significant at the p < 0.05 level.

```
cox <- coxph(Surv(protocol_week, status) ~ treatment, data = survival_data)

survfit <- survfit(Surv(protocol_week, status) ~ treatment, data = survival_data)

summary(cox)
```

```
## Call:
## coxph(formula = Surv(protocol_week, status) ~ treatment, data = survival_data)
## 
##   n= 162, number of events= 16 
## 
##                    coef exp(coef) se(coef)     z Pr(>|z|)  
## treatmentUrethane 1.355     3.876    0.756 1.792   0.0731 .
## ---
## Signif. codes:  0 '***' 0.001 '**' 0.01 '*' 0.05 '.' 0.1 ' ' 1
## 
##                   exp(coef) exp(-coef) lower .95 upper .95
## treatmentUrethane     3.876      0.258     0.881     17.06
## 
## Concordance= 0.616  (se = 0.044 )
## Likelihood ratio test= 4.41  on 1 df,   p=0.04
## Wald test            = 3.21  on 1 df,   p=0.07
## Score (logrank) test = 3.73  on 1 df,   p=0.05
```

```
# survfit %>%
#   ggsurvfit() +
#   add_risktable() + 
#   scale_ggsurvfit() +
#   annotate("text", x = 2, y = 0.05, label = glue::glue("{survfit2_p(survfit)}"))

ggsurvplot(fit = survfit,
                      pval = TRUE,
                      pval.method = TRUE,
                      risk.table = TRUE, 
                      conf.int = FALSE, 
                      xlab = "Time (Weeks)", 
                      palette = c("#E66100", "#5D3A9B"))
```
