# Supplementary material for: Dissecting Sex Chromosome and Hormonal Contributions to Urethane-Induced Lung Tumorigenesis Using the Four Core Genotypes Mouse Model
Source: Cancers (Basel). 2026 Apr 5;18(7):1172. doi: 10.3390/cancers18071172 (PMC13072358; doi:10.3390/cancers18071172)
Supplement: Supplementary file 1 [file cancers-18-01172-s001.zip › Supplementary Figure S1. Lymphoid Mass Histology.pdf]

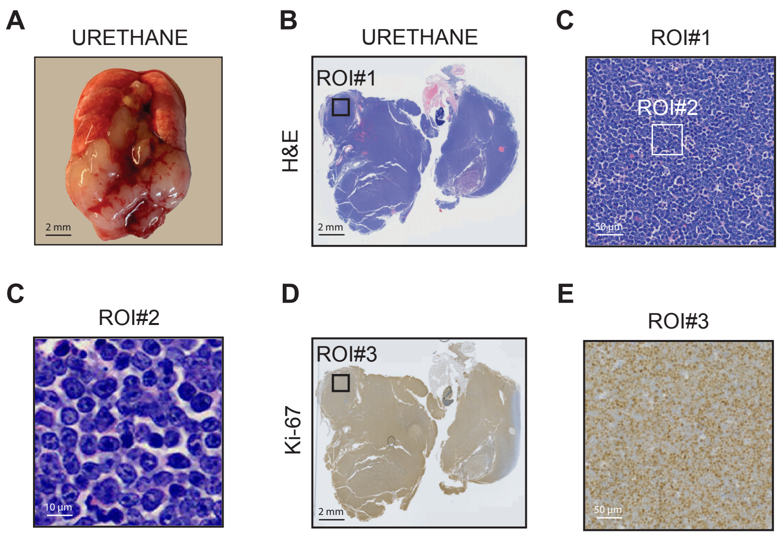

Supplementary Figure S1. Histologic and proliferative features of a lymphoid lesion identified in a urethane-treated mouse.

(A) Gross image of lungs from a urethane-treated mouse demonstrating a prominent mass located beneath the lung lobes (scale bar, 2 mm).
(B) Hematoxylin and eosin (H&E)–stained whole-lung section showing extensive expansion and effacement of normal pulmonary architecture by a dense lymphoid infiltrate (ROI#1 indicated; scale bar, 2 mm).
(C) Higher-magnification H&E images of ROI#1 and ROI#2 demonstrating sheets of relatively uniform lymphoid cells with high nuclear-to-cytoplasmic ratio and diffuse parenchymal involvement (scale bars, 50 µm and 10 µm, respectively).
(D) Ki-67 immunohistochemistry of the corresponding section (ROI#3 indicated; scale bar, 2 mm).
(E) Higher-magnification image of ROI#3 showing widespread nuclear Ki-67 positivity within the lymphoid population, consistent with a proliferative lymphoid process (scale bar, 50 µm).
