# Supplementary material for: Dissecting Sex Chromosome and Hormonal Contributions to Urethane-Induced Lung Tumorigenesis Using the Four Core Genotypes Mouse Model
Source: Cancers (Basel). 2026 Apr 5;18(7):1172. doi: 10.3390/cancers18071172 (PMC13072358; doi:10.3390/cancers18071172)
Supplement: Supplementary file 1 [file cancers-18-01172-s001.zip › Supplementary Methods S1. KRAS (Q61R) PCR Workflow.pdf]

WT and mutant (Q61R) Kras PCR Workflow

1. **PCR Reaction prep**

Prepare a 50 µL PCR reaction mix as follows:
- 10 µL of 5x Master Mix (Eazy PCR, RG-1000-01) (contains DNA polymerase, buffer, $MgCl_2$, dNTPs)
- 1 µL Forward primer (10 µM) final conc 0.2 microM
- 1 µL Reverse primer (10 µM) final conc 0.2 microM
- Needed volume of DNA template (100–300 ng) Start with 100 ng
- Add nuclease-free water to bring the final volume to 50 µL

2. **Thermal Cycling**

Run PCR with the following thermal profile:
- 95 °C for 2 minutes (initial DNA denaturation)
- 40 cycles of:
  • 95 °C for 15 seconds (denaturation)
  • Annealing at 59 °C for 15 seconds (Primer binding)
  • 72 °C for 30 seconds (DNA extension)
- 72 °C for 2 minutes (final extension)
- Hold at 4 °C

3. **Gel Electrophoresis**

Load 10 µL of PCR product (save the rest, 40 uL) on a 2% agarose gel with a DNA ladder. Run the gel and look for a band at the expected product size. Multiple or unexpected bands indicate nonspecific amplification.

4. Measure DNA concentration using NanoDrop. Aim for a concentration of 5–20 ng/µL for sequencing.

5. Prepare sample for Sanger sequencing

Prepare samples for submission:
- We are going to send the aliquot of the primers and give them the concentration of the primers.
- Provide both forward and reverse primers at 3.2 µM, 5–10 µL each
- Submit samples in separate, clearly labeled tubes (sample ID, primer name)
